# Multispot single-molecule FRET: High-throughput analysis of freely diffusing molecules

**DOI:** 10.1371/journal.pone.0175766

**Published:** 2017-04-18

**Authors:** Antonino Ingargiola, Eitan Lerner, SangYoon Chung, Francesco Panzeri, Angelo Gulinatti, Ivan Rech, Massimo Ghioni, Shimon Weiss, Xavier Michalet

**Affiliations:** 1 Department of Chemistry & Biochemistry, UCLA, Los Angeles, CA, United States of America; 2 Dipartimento di Elettronica, Informazione e Bioingeneria, Politecnico di Milano, Milan, Italy; University of Toronto, CANADA

## Abstract

We describe an 8-spot confocal setup for high-throughput smFRET assays and illustrate its performance with two characteristic experiments. First, measurements on a series of freely diffusing doubly-labeled dsDNA samples allow us to demonstrate that data acquired in multiple spots in parallel can be properly corrected and result in measured sample characteristics consistent with those obtained with a standard single-spot setup. We then take advantage of the higher throughput provided by parallel acquisition to address an outstanding question about the kinetics of the initial steps of bacterial RNA transcription. Our real-time kinetic analysis of promoter escape by bacterial RNA polymerase confirms results obtained by a more indirect route, shedding additional light on the initial steps of transcription. Finally, we discuss the advantages of our multispot setup, while pointing potential limitations of the current single laser excitation design, as well as analysis challenges and their solutions.

## 1 Introduction

### 1.1 Background

Freely-diffusing single-molecule FRET (smFRET) studies have yielded a wealth of new scientific results since their proof-of-principle demonstration almost two decades ago [1, 2]. While continuous improvements in fluorophores, labeling chemistry, excitation optics, theoretical analysis and combination with other techniques have increased its performance, there are still obstacles to the wide adoption of this powerful technology, chief among which is the issue of throughput.

Indeed, in order to ensure the separate detection of fast diffusing individual molecules, low concentrations, small excitation volumes and high excitation intensities are necessary. This is generally obtained with a so-called confocal geometry, where a laser is focused into a single diffraction-limited volume of the sample and a single-pixel detector collects light only from this volume. The low concentration needed to minimize the occurrence of simultaneous crossing of two or more molecules (<100 pM), lead to infrequent single-molecule transits so that several minutes, and in some extreme cases hours, are needed to accumulate a statistically significant number of transit events. These characteristics have thus far confined the technique to equilibrium measurements or very slow kinetics studies, with time scales on the order of several minutes [3].

Parallelizing measurements provides the necessary increase in throughput required for freely-diffusing fluorescence experiments. Rather than observing individual molecules sequentially in a single spot, different molecules are observed simultaneously at several independent locations within the sample. Camera-based approaches to smFRET [4] have a temporal resolution limited to a few ms, confining this approach to freely-diffusing molecules enclosed in small static volumes such as zero-mode waveguides [5], microscopically patterned PDMS wells [6], or tethered vesicles [7, 8]. Another, more complex approach for increased throughput involves extending the single-spot confocal geometry to a multispot confocal geometry. However, this requires parallel photon-counting capabilities, which have only become available recently with the development of single-photon avalanche diode (SPAD) arrays.

### 1.2 SPAD arrays

Recently, we described such a confocal multispot setup using a linear array of 8 SPADs for fluorescence correlation spectroscopy (FCS) analysis of single fluorescent dyes [9]. Our group and others have since demonstrated similar achievements with much larger SPAD arrays based on CMOS technology [10, 11]. These studies were however limited to working with labeled molecules at concentrations larger than the single-molecule regime (> 1 nM), mostly because of the limited detection efficiency and large dark count rates of some of these CMOS SPAD arrays.

The SPAD array used in our original FCS study was fabricated using a custom silicon technology developed by the Politecnico di Milano (POLIMI) group [12]. This dedicated technology results in better detection efficiency than CMOS SPAD arrays in the visible range and allowed us to demonstrate single-molecule sensitivity [9]. In follow-up experiments, we further demonstrated two-color smFRET measurements. We first used a single 8-SPAD array divided into two spectral detection channels each with 4 SPADs [13], and later extended this work to two arrays of 8 SPADs [14]. More recently developed SPAD arrays comprising even more SPADs open up the perspective of significantly higher throughput measurements [15, 16].

### 1.3 Multispot smFRET

Although the principle of multispot smFRET as described above is conceptually simple, its proper execution requires a number of careful checks. In particular, even though a single optical setup is used, each excitation spot and its corresponding detection volume, as well as associated SPADs, have unique characteristics, making this single experiment in effect a series of *N* parallel but slightly different experiments. The only true common denominator to all these parallel experiments is the sample itself. Any other parameter such as laser excitation power, excitation and detection point-spread functions (PSFs), photon detection efficiency (PDE), etc., cannot be assumed to be exactly identical from one spot to the next. Therefore, in order to be able to compare or pool data originating from different spots, a number of calibration and correction factors need to be determined and a robust data analysis approach needs to be designed and validated.

In other words, using a multispot setup to perform smFRET experiments forced us to look into a rigorous way to compare data about a given single-molecule system, acquired in potentially very different conditions. This effort should therefore hopefully be of general interest to the single-molecule fluorescence community, not only when dealing with multispot arrangements, but also when trying to compare results obtained from single-spot experiments performed on different setups.

This paper is organized as follows. We first briefly introduce the two experimental setups used in this work, a “standard” single-spot two-alternating lasers (μs-ALEX) setup used as reference, and a single-laser, two-color 8-spot setup whose detector characteristics are discussed (Section 2). Section 3 provides an overview of the smFRET analysis workflow used in this study, most of the details being presented in different appendices in the Supporting Information. Section 4 compares the smFRET results obtained for a series of dsDNA samples with both single-spot μs-ALEX setup (our gold standard) and multispot setup, thus validating our method. To showcase the advantage of the higher throughput of the multispot setup, Section 5 studies the escape kinetics of *Escherichia coli* (*E*. coli) RNA polymerase from a gene promoter region during DNA transcription initiation. We conclude with a brief overview of the main developments presented in this work, as well as an outlook on future improvements (Section 6). Samples and setup descriptions, extended discussion and analysis methods are available in Supporting Information.

### 1.4 Data and software availability

All datasets discussed in this paper (for both single and multispot experiments) have been uploaded on Figshare and are thus citable (references can be found in Appendix 1 in S1 File). Datasets are stored in the Photon-HDF5 file format, an open multi-platform file format for timestamp-based single-molecule fluorescence experiments [17].

Data analysis was performed with our open source FRETBursts Python software [18] and with our ALiX LabVIEW software available as a standalone executable, with some fits performed with Origin 9.1 (OriginLab). To ensure computational reproducibility, we did our best efforts to document and report all analysis details. Analysis performed with FRETBursts can be replicated by running the main Jupyter notebooks associated with this paper, linked to in Appendix 2 in S1 File. Analysis performed with ALiX can be reproduced using scripts linked to in Appendix 2 in S1 File.

## 2 Setup description

**Fig 1:**
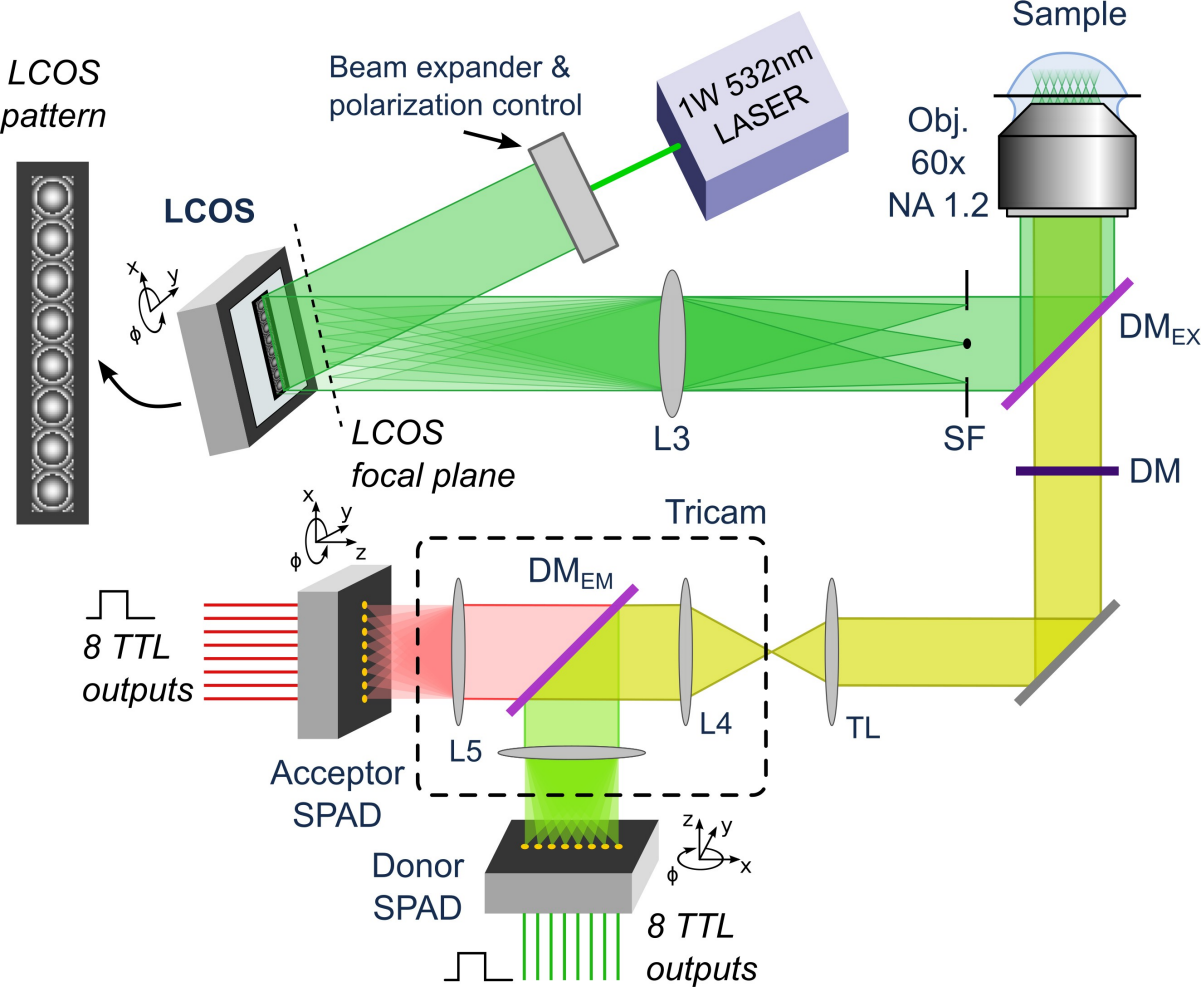
Schematic of the 8-spot single-molecule FRET setup. A freely-diffusing single molecule sample is probed via 8 independent excitation spots generated by a 532 nm CW laser and an LCOS spatial-light modulator. The fluorescent signal from each excitation spot is optically conjugated to a pair of pixels in the two detectors. The donor and acceptor emission are separated in two distinct spectral bands and detected by two different SPAD arrays.

Two setups were used in this work. A “classic” single-spot setup (described in ref. [19] and in Appendix 3 in S1 File) designed around an inverted microscope was used for single-spot μs-ALEX measurements (Fig SI-1 in S1 File). The setup used for multispot smFRET measurements was designed around an identical microscope but used different lasers, optics and detectors, (Fig 1, Appendix 3 in S1 File, and ref. [14]). A brief description of the multispot detector characteristics is provided below.

The two SPAD arrays used in this study were similar to the one described in ref. [9]. Four main characteristics are of interest for this work: detection efficiency, dark count rate, afterpulsing probability and crosstalk probability.

### 2.1 Detection efficiency

The SPAD arrays fabricated using the standard custom technology developed by POLIMI are characterized by a similar PDE as that of the single SPAD module commercialized by Micro Photon Devices (Fig SI-2 in S1 File) [12, 20, 21] and used for donor (D) detection in the single-spot μs-ALEX setup. In particular, their photon detection efficiency in the acceptor (A) detection range (red area in Fig SI-2 in S1 File) is about half of that of the red-enhanced SPAD used for acceptor detection in the single-spot μs-ALEX setup. These characteristics result in a noticeable difference in correction factors between the two types of measurements, as discussed later in the text.

### 2.2 Dark count rates

Dark counts are random and due to thermally excited charge carriers in the detection area, leading to an avalanche. Dark count rates (DCRs) measured for the two 8-SPAD arrays are reported in Table 1. The range of values is widespread, with more than two orders of magnitude separating the largest and smallest dark count rates. In particular, half the SPADs used for the acceptor channel have a DCR comparable to or larger than the typical sample background rate (from 1 to 3 kHz depending on sample and spot). The influence of these large DCR values for analysis will be addressed later in the text.

**Table 1:** Dark count rates. Dark count rates (DCR, in Hz) for individual SPADs of the two SPAD arrays used in this work. D-channel and A-channel indicates the 8-SPAD array used to collect, respectively, the donor and acceptor signal from each spot. The smallest (24 Hz) and largest (7 kHz) DCR are highlighted. The top row indicates the pixel number in each array. Under the assumption of Poisson statistics, the standard error is equal to the square root of the estimated rates.

	1	2	3	4	5	6	7	8
D-channel	36	117	3,775	567	29	443	27	24
A-channel	3,679	7,207	78	901	1,717	4,321	49	53

### 2.3 Afterpulsing probability

Afterpulses are the result of trapped carriers generated during an avalanche and released shortly after it. The integrated afterpulsing probability of each SPAD was measured under moderate constant illumination according to the simple relation [22]:

$$p_{a}=\frac{1}{2}Q+\lambda \tau_{d},$$ (1)

where *Q* = *Var(S)/<S>* - 1 is the Mandel parameter computed for the measured detector signal *S*, *λ* is the incident count rate and *τ*_*d*_ = 120 ns is the detector deadtime. *Q* depends in general on the time bin *T* used to record signal *S*, but for a moderate constant illumination (*λ τ*_*d*_ << 1), this dependence is negligible and the afterpulsing probability is simply half the Mandel parameter. The resulting afterpulsing probabilities are reported in Table 2 (see details in Appendix 4.2 in S1 File).

**Table 2:** Afterpulsing probabilities. Estimated afterpulsing probabilities (in percent) and their standard deviation for each SPADs of the two arrays. The top row indicates the pixel number in each array. The standard deviation is based on three independent measurements.

	1	2	3	4	5	6	7	8
D-Channel	1.81 ± 0.70	1.79 ± 0.06	2.21 ± 0.01	1.61 ± 0.46	1.84 ± 0.50	2.43 ± 0.41	2.10 ± 0.41	1.41 ± 0.38
A-Channel	5.11 ± 0.10	5.69 ± 0.02	12.03 ± 1.69	2.94 ± 0.60	4.93 ± 0.10	3.56 ± 0.43	4.54 ± 0.06	4.77 ± 0.07

They are larger than for single-pixel SPADs, which have typically *p*_*a*_ < 0.1%, but, when properly accounted for by the different correction factors discussed later in the text, do not affect the results.

### 2.4 Crosstalk

In the multispot setup, additional source of noise originates from electrical or optical crosstalk between pixels. The electrical crosstalk can be made negligible with a proper electrical design of the SPAD array module. The optical crosstalk, instead, can be internal to the SPAD array or external, originating from the optical system. In the first case, the optical crosstalk is due to secondary photons emitted by SPADs during the avalanche current [23]. In the second case, it is due to the emission point spread function (PSF) targeted to a given pixel spilling over the detection PSF of a nearby pixel. In the multispot system, the ratio between PSF pitch and width is 5, resulting in negligible external crosstalk. We measured the internal optical crosstalk in the two SPAD arrays used in the multispot setup and found it to be smaller than 0.5% for all pixel pairs (see Appendix 4.1 in S1 File), resulting in negligible effects on burst analysis.

## 3 smFRET data analysis

smFRET data analysis of freely-diffusing single-molecule involves a number of steps which have been described with some details in the literature [18, 24–28]. However, our experience is that data analysis reproducibility is oftentimes hampered not only by the lack of raw data files, but also by the absence of values for specific parameters and algorithms implementation details. Here, we strived to provide as much information as possible in the main text and Supporting Information, complemented by algorithmic implementation when feasible. We found that analysis of multispot data can be made relatively straightforward, but requires some care and cannot be oversimplified.

For the interest of legibility, the main text only introduces concepts and notations, details being provided in the Supporting Information.

### 3.1 Photon streams definition

In μs-ALEX measurements, 4 streams of photon timestamps (or photon streams) can be distinguished, functions of the excitation period during which the timestamps are recorded and their detection channel [29, 30]. We will denote them as D_ex_D_em_, D_ex_A_em_, A_ex_A_em_ and A_ex_D_em_. These streams identify photons detected during the D- or A-excitation period (D_ex_ or A_ex_) and by the D- or A-detection channels (D_em_ or A_em_).

By contrast, for single-laser smFRET measurements, such as done with the multispot setup, there is only one excitation period (the donor period) and therefore only two photon streams: D_ex_D_em_ and D_ex_A_em_. We will denote them as D_em_ and A_em_ for brevity.

Details on how photon streams are defined based on timestamp information can be found in Appendix 5 in S1 File.

### 3.2 Background rate estimation

Background rates, noted *b*_*stream*_, where the photon stream are defined in the previous section (e.g. *b*_*DexDem*_, *b*_*DexAem*_, etc.), were measured for each photon stream. As discussed in Appendix 6 in S1 File, different physical mechanisms contributing uncorrelated counts give rise to an exponential tail in the distribution of inter-photon delays [31]. At short delays, the inter-photon delay distribution departs from a pure exponential due to the contribution of single-molecule bursts [31]. Defining a cut-off time *τ*_*min*_ as the delay above which the distribution is considered exponential, the count rate corresponding to the exponential tail can be simply calculated by the maximum likelihood estimator (MLE):

$$b={\left({\langle \tau_{i}\rangle }_{\tau_{i}>\tau_{\min}}-\tau_{\min}\right)}^{-1},$$ (2)

where the average is taken over all delays *τ*_*i*_ *> τ*_*min*_ [18]. Choosing *τ*_*min*_ involves some trade-off, but can be automated (Appendix 6 in S1 File). In addition, since background rates can occasionally vary during the measurement, we estimated background rates over consecutive time windows of, typically, 30 s.

For the multispot measurements, background rates for each of the two available streams (D_em_ and A_em_) were estimated separately for each spot.

### 3.3 Burst search

The burst search algorithm used in this work is based on the sliding window algorithm introduced by the Seidel group [24, 32] (see also [18]). Burst search can be specified by three parameters: type of photon stream, number *m* of consecutive photons used to compute the local count rate (typically, *m* = 10), and count rate threshold *r*_*min*_. This threshold can be defined in different ways. When defined as a multiple of the background rate (*r*_*min*_ *= F b*_*stream*_), it will follow background variations during the measurement and ensure that a minimal burst signal-to-background ratio (SBR) is achieved (minimum SBR burst search [33, 34]). Alternatively, the threshold can be set to a fixed value (minimum count rate burst search). The choice between these two approaches is dictated by the specific aim of the analysis, as will be discussed later.

A detailed description of various burst searches and their influence on different observables can be found in Appendix 7 in S1 File.

### 3.4 Burst selection criterion

The outcome of a burst search as just described is in general a large set of bursts, the majority of which are comprised of very few photons (Fig SI-14A&B in S1 File). Because *m* photons are used for the search, the minimum burst size before background correction is *m*, which in general is a small number (*e*.*g*. *m* = 10). These small bursts contribute very little useful information. Moreover, they tend to increase the variance of observables such as the proximity ratio (*PR*) distribution, as discussed below and in Appendix 8 in S1 File. This makes the separation of populations characterized by similar proximity ratios, such as a low FRET population and D-only population, more difficult in the absence of laser alternation. For these reasons, rejecting bursts whose size is smaller than a preset minimum value *S*_*min*_ is a natural step in all analyses, although other criteria can be used and combined for specific purposes (e.g. minimum burst duration, minimum count rate, etc.). Further discussion of this burst selection step and the influence of parameter *S*_*min*_ on analysis results can be found in Appendix 8 in S1 File.

### 3.5 Proximity ratio and FRET efficiency analysis

smFRET studies only aiming to distinguish between different populations of molecules on the basis of their FRET efficiencies *E* do not necessarily require the precise calculation of *E*. The proximity ratio *PR* can be used instead, which involves only background corrected quantities:

$$\mathrm{PR}=\frac{n_{A_{\mathrm{em}}}}{n_{A_{\mathrm{em}}}+n_{D_{\mathrm{em}}}},$$ 3

where $n_{D_{\mathrm{em}}}$ and $n_{A_{\mathrm{em}}}$are the background-corrected donor and acceptor signals (upon donor excitation) respectively. These quantities depend on the different correction factors discussed next, and therefore, any two measurements of the same sample performed on different setups may results in different values of *PR*. For an exact comparison between these measurements, corrected quantities (*i*.*e*. the FRET efficiency *E*) need to be computed. The relation between proximity ratio, FRET efficiency and those correction factors has been described previously [30] and is reminded in Appendix 9 in S1 File.

Similarly, in a multispot setup, where correction factors may be spot-dependent, computation of correction factors first requires calculation of proximity ratio values, and comparison of results from different spots requires calculation of corrected FRET efficiency values.

Statistical analysis of the proximity ratio and FRET efficiency was performed by 3 different methods: Gaussian fit of their histogram, kernel density estimation (KDE) and shot noise analysis (SNA), as discussed in Appendix 10 & 11 in S1 File.

### 3.6 Correction factors

Several correction factors are necessary to convert raw photon count quantities into physical observables such as FRET efficiency. These parameters are:

- the *donor leakage* factor *l*, quantifying the donor signal detected in the acceptor channel as a fraction of the donor channel signal,
- the *direct acceptor excitation* factor *d*, quantifying the amount of acceptor signal due to direct excitation by the donor excitation laser,
- the fluorescence quantum yield and detection efficiency correction factor *γ*, accounting for the different brightness of the donor and acceptor dye due to their different fluorescence quantum yield and different detection efficiency in their respective channels.

Appendix 9 in S1 File provides details on how these correction factors were estimated in the single-spot μs-ALEX and multispot experiments respectively. Here, we briefly summarize the differences between the two types of experiments.

In single-spot μs-ALEX experiments, it is possible to estimate the donor leakage and direct acceptor excitation factors in each sample, as long as there are enough donor-only (DO) and acceptor-only (AO) bursts, respectively. Indeed, each population (DO, AO and donor and acceptor-labeled molecules, DA) can be readily isolated in the so-called two-dimensional ALEX histogram representing the stoichiometry ratio (*SR*) of each burst as a function of its proximity ratio (*PR*) (Appendix 9 in S1 File). Factor *l* can be computed from the DO *PR* histogram, while factor *d* can be computed from the AO *SR* histogram. These coefficients should not depend on the sample (provided the dyes are in similar chemical environments) or the specific measurement (as long as nothing has changed in the setup), but it is always useful to be able to confirm it. Average factors were used in the final analysis. In general, it is recommended to use dedicated DO and AO samples to measure these correction factors.

The single-spot μs-ALEX *γ* factor was estimated according to Lee *et al*. [30] with the series of 5 dsDNA samples as described in Appendix 9 in S1 File.

In multispot experiments, the absence of acceptor laser excitation prevents classification of single-molecule bursts based on the stoichiometry ratio, leaving the proximity ratio as the only observable to separate between DO and DA bursts (AO bursts are in general not detected). While *PR* is sufficient to distinguish between high FRET and DO bursts, the overlap between *PR* distribution of both types of molecules increases as the FRET value in the sample decreases, which makes it difficult if not impossible to separate DO and DA populations in low FRET samples. For this reason, a DO sample was used in addition to the highest FRET samples of the dsDNA series for extracting the *l* factor.

It is possible to correct for the acceptor direct-excitation contribution (*Dir*) in multispot measurements employing a single laser excitation (Eqs. (SI.53)-(SI.56) Appendix 9 in S1 File), if the direct excitation coefficient *d*_*T*_ *= σ*_*A*_ */ σ*_*D*_ (ratio of acceptor and donor absorption cross-sections at the donor excitation wavelength) is known. This ratio, being a molecular characteristics, is independent from the setup or experiment used to compute it. We therefore estimated *d*_*T*_ using a single-spot μs-ALEX measurement. Finally, the *γ* factor for the multispot system was estimated from the corrected *E* obtained from μs-ALEX measurements of one sample (12d) used as reference (see Appendix 9 in S1 File for details).

### 3.7 Fluorescence correlation spectroscopy

FCS analysis was used to characterize the excitation/detection volumes of the donor and acceptor channel for each spot of the multispot measurements. The analysis is relatively straightforward in this situation where a single sample, characterized by a unique diffusion coefficient, is observed at different locations. Any difference in diffusion time can be therefore interpreted as due to differences in excitation/detection volume. The results were compared to those obtained in the single-spot μs-ALEX measurements.

Details on the analysis, which introduces some new correction for μs alternation, and involves correcting for large afterpulsing in the multispot experiments, are presented in Appendix 12 in S1 File.

## 4 dsDNA smFRET measurements

To fully characterize the performance of the multispot setup, we performed smFRET measurement on a series of doubly-labeled dsDNA samples using a standard single-spot μs-ALEX setup and our 8-spot setup. All samples possess the same 40 base-pair (bp) sequence used in previous works [19, 30], one strand being labeled with the acceptor dye (ATTO647N) at its 5’-end, while the complementary strand was labeled internally with the donor dye (ATTO550). The donor position depends on the sample, and was chosen so that the D-A separation were 7, 12, 17, 22 and 27 base pairs. The sequence was designed such that the 3 base pairs surrounding each donor labeling sites were identical at each locus (see Appendix 13 in S1 File). We will refer to these six samples as the 7d, 12d, 17d, 22d, 27d and DO (D-only) samples. The results presented in this section are based on analysis of 6 single-spot μs-ALEX measurements and 6 multispot smFRET measurements, one measurement for each sample.

### 4.1 Single-spot results

Single-spot μs-ALEX measurements were performed on a dedicated setup and at a different time than the multispot experiments. Moreover, although the same sample stocks were used, sample preparation was distinct, and therefore the concentration and respective fraction of singly- and doubly-labeled molecules in the two types of experiments were most certainly different.

In addition to sample concentrations, many other acquisition parameters may have differed between the two sets of experiments (e.g. excitation intensity, background levels, correction factors, etc.), all of which could potentially affect the results in both types of measurements. In order to be able to compare data acquisition parameters in single-spot experiments with those of the multispot experiments, we characterized several independent observables: mean count rates and background count rates, peak burst count rates, burst number per unit time and FCS analysis. In the interest of space and in order to focus on the multispot results, characterization of the single-spot setup can be found in Appendix 14 in S1 File.

**Fig 2:**
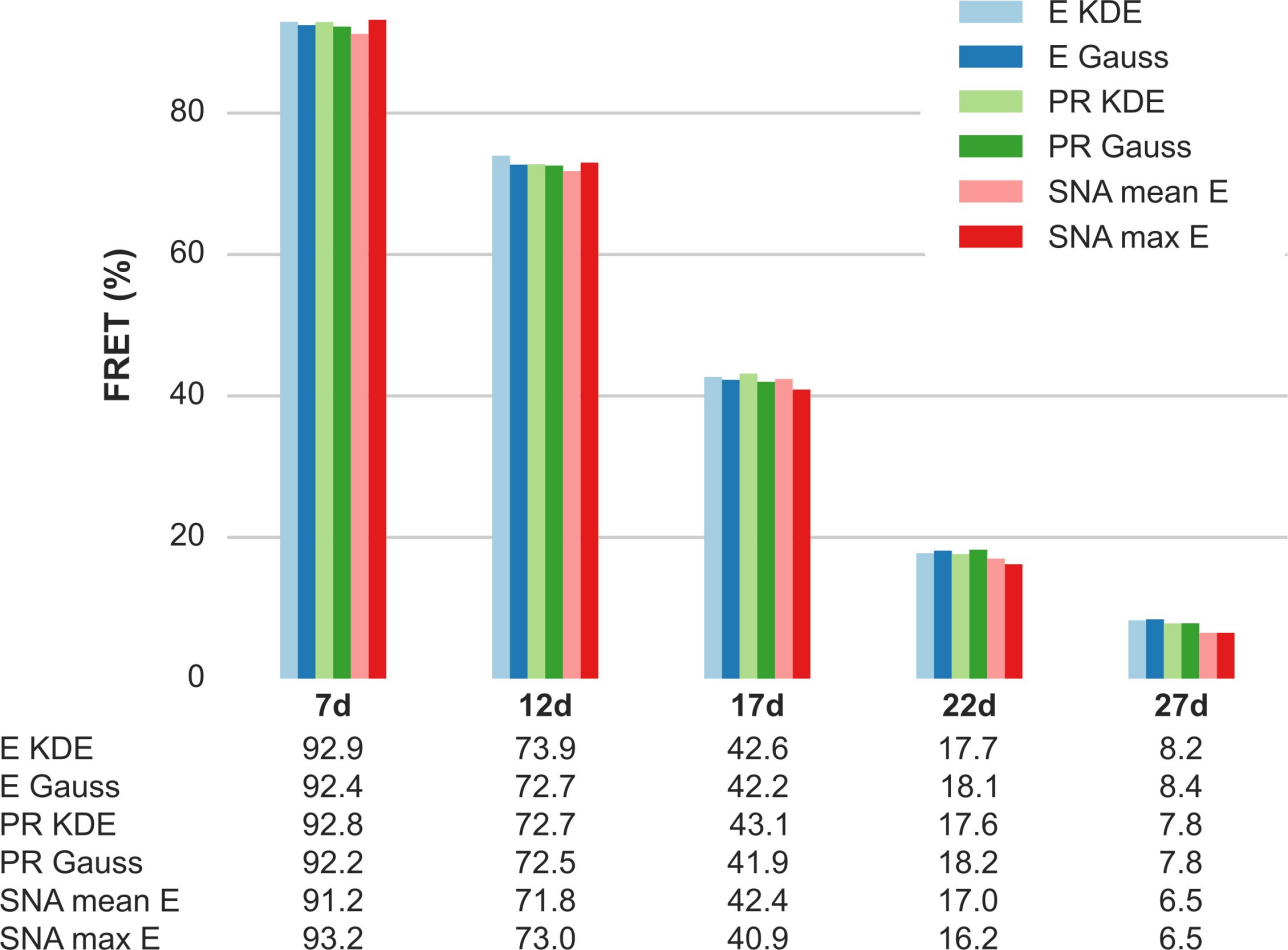
Single-spot μs-ALEX smFRET efficiency for 5 dsDNA samples estimated using different methods. Population-level FRET efficiency estimated for each of the 5 dsDNA samples: 7d, 12d, 17d, 22d and 27 (the number represents the separation in base-pair between D and A dyes). FRET is estimated using the following methods. *E KDE*–KDE maximum from the corrected E distribution. *E Gauss*–Gaussian fit of the corrected E histogram. *PR KDE*–KDE maximum of the *PR* distribution, *PR* value converted to *E*. *PR Gauss*–Gaussian fit of the *PR* histogram, *PR* value converted to *E* (all previous estimation are described in Appendix 9 in S1 File). *SNA mean E*–Mean of the FRET distribution returned by SNA analysis. *SNA max E*–Mode of the FRET distribution returned by SNA analysis (SNA analysis described in Appendix 11 in S1 File). For full details on the analysis (including number of bursts and fit errors) see section of *μs-ALEX*: *Corrected E figure* of the accompanying Jupyter notebook (view online). An overview of the computational notebooks can be found in Appendix 2 in S1 File.

smFRET analysis of each sample was performed as outlined in Section 3. Donor leakage was extracted from the D-only population proximity ratio (*PR*) peak and the acceptor direct excitation from the A-only population stoichiometry ratio (*SR*) peak, as described in details in Appendix 10 in S1 File. We then applied these two corrections to the PR and SR distribution, to obtain an estimate of the *γ* correction factor [30]. In Fig 2 we report a comparison of corrected FRET efficiencies (*E*) for each sample, estimated using different methods (see details in Appendix 10 & 11 in S1 File). In all cases, the FRET population was isolated by selecting bursts with *D*_*ex*_ counts ≥30 and within an appropriate region of interest in the ALEX histogram.

*PR KDE* are the results of kernel density estimation (KDE) of the PR distributions, and report the position of the KDE maximum, corrected as described in Appendix 9 in S1 File to obtain *E KDE*. *PR Gauss* are the peak positions of a Gaussian fit to the PR histograms, while *E Gauss* values are obtained by the same correction mentioned for *PR KDE*. Finally, the last two values report results of shot-noise analysis (SNA, discussed in Appendix 11 in S1 File). *SNA mean E* are the values of the mean of the fitted *E* probability distribution and *SNA max E*, the modal values of these distributions. Fig 2 reports FRET efficiencies estimated with all these methods on a set of 5 measurements (one for each D-A distance). Results of Fig 2 show that all methods are in very good agreement with each other.

### 4.2 Multispot results

As in the single-spot μs-ALEX experiments (Appendix 14 in S1 File), potential differences in sample concentration, excitation intensity, or other setup characteristics were investigated thoroughly. The multispot geometry introduces additional degrees of variability, due to possible differences between excitation spots geometry and SPAD alignment.

As described in Appendix 3 in S1 File, the excitation spots are generated by direct phase modulation of an expanded Gaussian beam. Because the Gaussian beam is centered in the middle of the linear pattern, lateral spots exhibit a lower intensity than the center ones. In addition, due to the size of the excitation pattern (> 30 μm in the sample plane, see Appendix 3 in S1 File), some geometric aberrations are expected towards the edge of the pattern. While these different imperfections can be characterized by direct imaging [9, 35], this is a time-consuming process, not suitable for routine characterization. Moreover, information obtained by this kind of measurement cannot be used straightforwardly for data analysis corrections. Instead, we examined different measures of the differences between spots extracted from the data itself, using several burst statistics as discussed next (details in Appendix 15 in S1 File).

Finally, smFRET analysis involves correction factors (Section 3.6) which could potentially depend on the spot under consideration. We therefore characterized their dispersion, as described in the following. However, we obtained highly uniform FRET histograms across the 8 spots (with peaks positions varying by at most 2%) without the need to apply channel-dependent corrections for γ-factor, donor-leakage and acceptor direct excitation.

#### 4.2.1 Signal versus spot

For any given single-molecule sample, the *total photon collection efficiency* of a spot depends on its excitation PSF, as well as the detection PSF due to optics and detector. In freely-diffusing single-molecule experiments, single-molecule bursts are superimposed to a stationary or slowly varying background coming from detector dark counts and sample background. In single-spot μs-ALEX measurements, the contribution of SPAD dark count rate (DCR) was negligible (<250 Hz). For the multispot measurements, however, some of the detectors had DCR which dominated other sources of background (see Table 1). By taking the total count rate minus the DCR, we obtain a “signal” proportional to the product of excitation and detection efficiency which can be used to compare the collection efficiency (excitation + detection) of different spots. This approach assumes that the sample background arises mainly from fluorescence of out-of-focus molecules.

**Fig 3:**
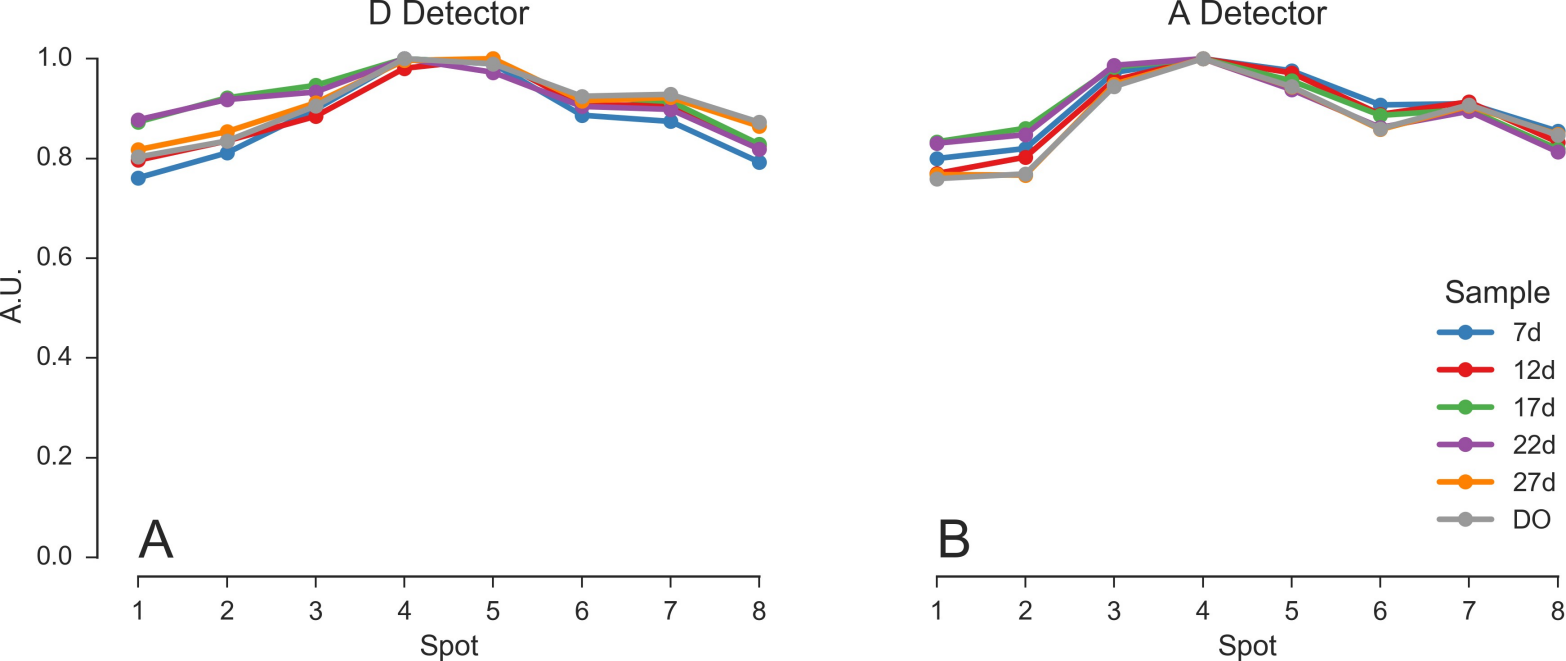
DCR-corrected total count rate as a function of the pixel. Characterization of the excitation × detection profile as a function of spot. Values computed from DCR-corrected count rate in each pixel for different samples. (A) Signal detected by the SPAD array in the D emission channel. (B) Signal detected by the SPAD array in the A emission channel. The samples are the 5 doubly-labeled dsDNA samples (7d - 27d) plus the D-only dsDNA sample (DO). For each sample, the signal is normalized to 1 for the detector with the highest signal. For computational details see section *Signal vs spot* (view online) of the accompanying Jupyter notebook.

Fig 3 shows the DCR-corrected total signal discussed before as a function of detector for the 6 dsDNA samples. In each measurement, the signal is normalized to 1 for the detector with the highest signal. A clear signal reduction in the lateral spots compared to the central ones is visible, which reflects in part the expanded Gaussian beam profile used to generate the pattern.

This variation of the signal across spots may be due to variation in the excitation intensity and detection efficiency, indicating that either one or both change as we move from the center of the spot pattern toward its edges. FCS analysis provides complementary information helping to remove the ambiguity, as discussed in the next section.

#### 4.2.2 FCS analysis

Fluorescence correlation spectroscopy (FCS) analysis of each spot signal can in principle provide information on sample concentration and diffusion constant, if the excitation/emission PSFs are known. In multispot experiments, the diffusing species is identical for each spot, therefore, FCS analysis can be used to detect and quantify differences in excitation/emission PSFs among spots (Appendix 12 in S1 File).

**Fig 4:**
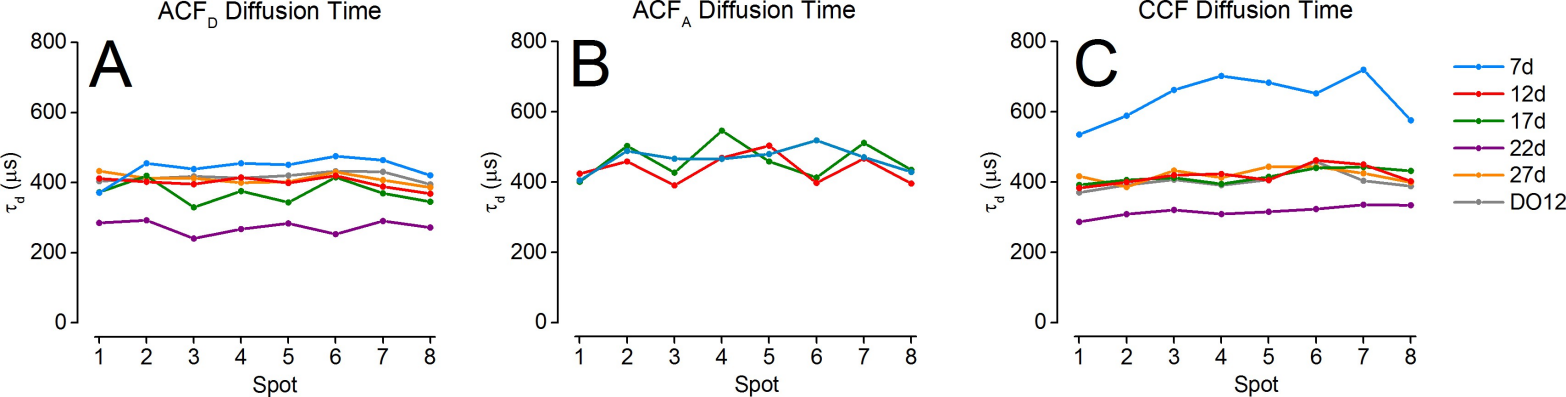
Multispot FCS analysis. For each sample, 8 donor autocorrelation functions (ACFs) and for samples with sufficient acceptor signal (7d, 12d and 17d), 8 acceptor ACFs, were fitted with a 2D diffusion model with multi-exponential afterpulsing components. Additionally, for all samples, the cross-correlation function (CCF) of the 8 donor and acceptor signals were computed. Each curve was fitted with the respective model described in the text. (A) Diffusion times for the donor ACFs. (B) Diffusion times for the acceptor ACFs. (C) Diffusion times for the donor-acceptor CCFs. A significantly shorter diffusion time is observed for the 22d sample in both the ACFs and CCFs. Sample 7d is characterized by a significantly larger diffusion time in the CCF only.

Fig 4 represents the diffusion times obtained from fitting donor or acceptor autocorrelation functions (ACFs) and of the donor-acceptor cross-correlation functions (CCFs) for the different samples (curves shown in Fig SI-20 in S1 File), whose average and standard deviations are reported in Table 3. Because of very low to non-existent acceptor signal, no fits of the acceptor ACFs could be performed for samples DO, 27d and 22d.

**Table 3:** 8-spot average of diffusion times (μs) fitted from ACF and 2-color CCF curves. Average diffusion times (*±* 1 standard deviation) obtained from correlation function fits (in μs). *τ_G_*: donor-ACF, *τ_R_*: acceptor ACF, *τ_GR_*: donor-acceptor CCF diffusion time obtained for a 2D diffusion model with no offset. No fit was performed for the acceptor ACF (A-ACF) of sample DO, 27d and 22d due to lack of sufficient acceptor signal.

	DO	27d	22d	17d	12d	7d
*τ*_*G*_	416 ± 13	411 ± 15	273 ± 19	371 ± 33	400 ± 17	441 ± 33
*τ*_*R*_				462 ± 53	438 ± 42	466 ± 35
*½(τ*_*G*_ *+ τ*_*R*_*)*				417 ± 34	419 ± 24	454 ± 33
*τ*_*GR*_	402 ± 26	420 ± 21	317 ± 16	417 ± 20	418 ± 26	640 ± 66

For each sample, apparent diffusion times from ACFs are relatively uniform among spots (~400 μs), suggesting that the observation volumes of the different spots are similar. Moreover, acceptor ACF diffusion times are only marginally larger than their donor counterpart, suggesting a minimal chromatic effect. Of particular note, however, is the systematically reduced diffusion time of ~300 μs observed across all spots for the 22d sample (Fig 4A, purple). A possible explanation for this observation (detector misalignment) will be discussed after we examine the complementary information provided by CCF analysis.

The CCF fits provide similar diffusion times (*τ*_*GR*_) for most samples. In particular, a lower diffusion time is computed for sample 22d as well. Since for donor-acceptor CCF, the diffusion time is theoretically equal to the mean of donor and acceptor diffusion times [36]:

$$\tau_{\mathrm{GR}}=\frac{1}{2}\left(\tau_{G}+\tau_{R}\right),$$ (4)

a lower value indicates that the corresponding acceptor channel ACF diffusion time *τ*_*R*_ (which could not be measured due to insufficient acceptor signal in sample 22d) is comparably small. Eq (4) is indeed verified for sample 12d and 17d, for which both *τ*_*G*_ and *τ*_*R*_ are available (Table 3).

For sample 7d, however, the CCF exhibits a much larger diffusion time and larger variance than their ACF counterparts (~650 μs versus ~450 μs), suggesting that misalignment of one of the two detector arrays is responsible for this discrepancy. Using Eq. (SI.83), with parameter *τ*_*GR*_ fixed for all spots to the value obtained from Eq (4), a value of the relative shift between donor and acceptor PSFs, *d*/*ω*_*GR*_ = 0.56 ± 0.06 was obtained, where the uncertainty is equal to the standard deviation over the 8 spots (see Appendix 12 in S1 File). Assuming a typical PSF waist parameter, *ω*_*GR*_ ~ 100 nm, this corresponds to a *d* ~ 60 nm shift in the sample plane, or equivalently, taking into account the 60× magnification of our setup, a ~ 4 μm shift in the detector plane. This is a rather small shift, which does not significantly affect the number of photons collected during each burst.

#### 4.2.3 PSF characterization by burst analysis

When the sample is homogeneous, as is the case across the spots in the multispot system, and burst search criteria are chosen appropriately (as discussed in Appendix 7 in S1 File), burst statistics such as *burst size* and *burst duration* can be used to characterize the product of the excitation-detection PSF. Burst size, for instance, is directly related to the integral of the excitation-detection PSF along the molecule’s trajectory, while burst duration is related to the PSF size. Additionally, the *peak count rate* reached in each burst depends directly on the peak excitation PSF intensity. All these burst statistics are characterized by probability densities with an asymptotic exponential behavior due to diffusion and the profile of the excitation/detection volume [31]. This property allows estimating mean values for each of these burst statistics, as described in Appendix 15 in S1 File.

Using these statistics, one needs to keep in mind that burst search parameters may influence some of them, such as burst size and duration distribution. For instance, the count rate is low at the beginning of a burst, increases and eventually decreases at the end. Therefore, considering a particular burst, increasing the photon rate threshold used for burst search, will reduce its duration and size (the burst will start later and end earlier, everything else being equal).

On the other hand, the peak count rate during a burst is typically attained well within the burst and therefore it is expected to be less, if at all dependent on the count rate threshold used for burst search.

Using a minimum SBR burst search, a larger background results in an increase in the count rate threshold used for burst search (Section 3.3). This causes a reduction in burst duration and size for spots characterized by a larger background rate, other spot characteristics being identical. This situation is relevant here, since a significant fraction of the background is due to detector dark counts, which differ significantly between SPADs within each array (Section 2.2). The peak count rate for identical species, however, should not be affected, provided all other acquisition parameters are identical (e.g. excitation intensity and alignment).

**Fig 5:**
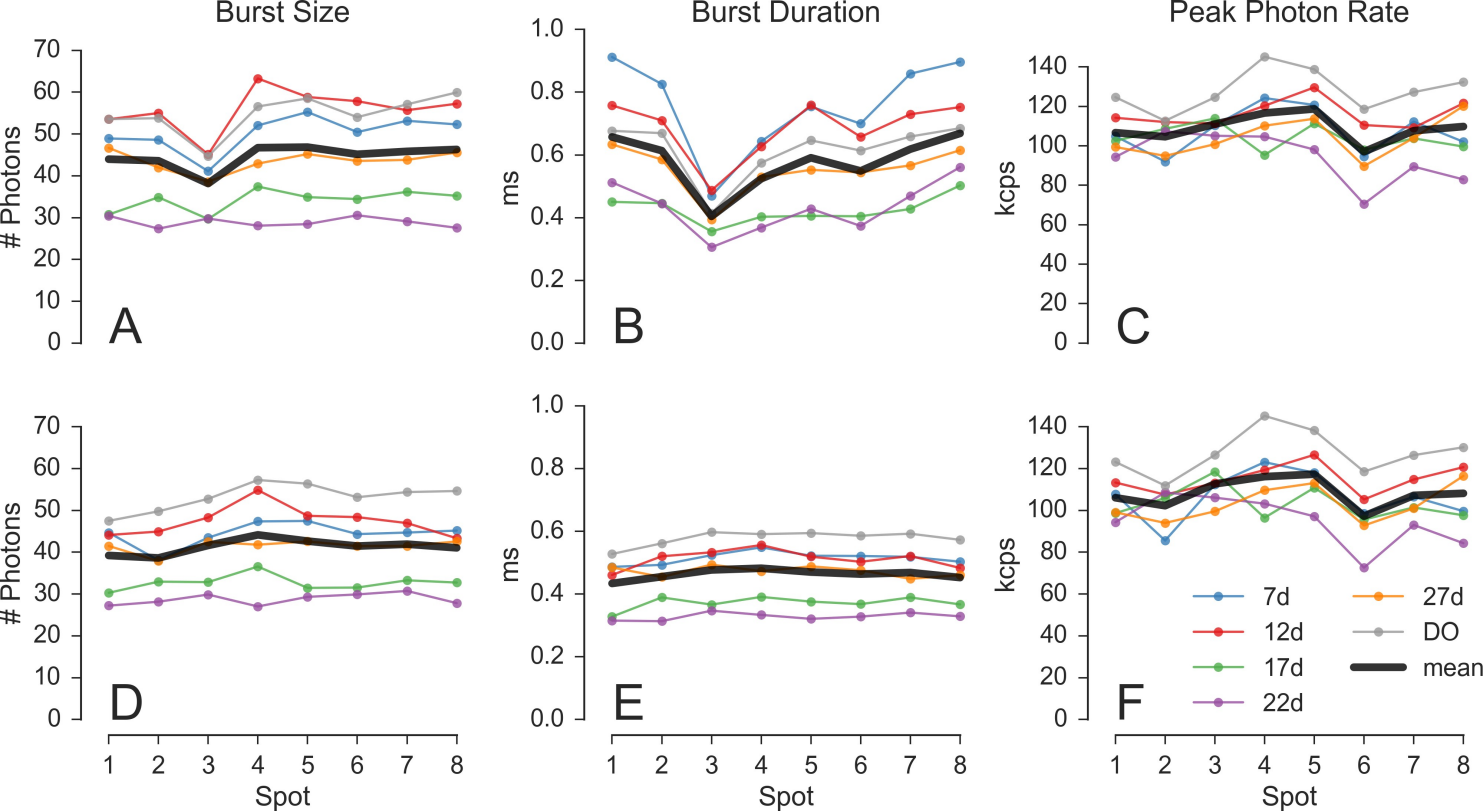
Burst statistics versus spot. Burst statistics extracted for the D-only population in the series of 6 dsDNA samples (7d, 12d, 17d, 22d, 27d and DO) using burst search on D_em_ photons. The first row (A, B, C) shows results obtained with a minimum SBR criterion burst search. The second row (D, E, F) show results obtained with a fixed threshold burst search. The three columns report different burst statistics as a function of the spot number. A, D: Mean burst size. B, E: Mean burst duration. C, F: Mean burst peak count rate. While the peak count rate is largely invariant from the type of burst search (C & F), burst size and duration are affected. In particular, the uneven DCR distribution of the donor SPAD array (maximum DCR in pixel 3), causes a dip in both mean burst size and duration (A & B). The DCR influence is eliminated when using a fixed threshold (D & E). Graphs D—F show the fairly uniform properties of the excitation-detection PSFs across the different spots (see main text). Additional computational details can be found in Section *Burst statistics vs spot* (view online) of the accompanying Jupyter notebook.

Fig 5 shows the effect of different burst searches on DO burst statistics for each of the 6 dsDNA samples. Focusing on the DO population eliminates complexities introduced by the different measured brightnesses of the FRET population of each sample (Appendix 15 in S1 File). The top row (Fig 5A–5C) shows results obtained with a minimum SBR criterion burst search, while the second row (Fig 5D–5F) reports results obtained with a fixed threshold burst search.

Burst size and duration computed in the first case (panel A & B) both show a dip for spot 3, whose SPADs are characterized by the highest DCR (Table 1). By contrast, burst size and duration computed in the second case (panel D & E) show a flatter profile, independent from DCR, suggesting minimal differences between spots. Differences from one experiment to the next can be observed, which can be explained by different excitation intensities.

Fig 5C & 5F show the mean peak count rate for both types of burst search. Their similarity demonstrates that, as expected, the peak count rate is largely independent from the burst search parameters (unlike burst size and duration). The mean peak count rate is therefore a reliable reporter for the excitation-emission PSF peak intensity.

Finally, looking at Fig 5D & 5E, we observe that, in agreement with the results of FCS analysis, the characteristics of the different spots in a given experiment are fairly uniform. Burst analysis also reproduces the difference between the measured samples obtained from FCS. In particular, the mean burst duration (*i*.*e*. the PSF width, since all samples are characterized by the same diffusion coefficient) is lower for samples 22d and 17d, suggesting differences in alignment in these measurements.

These results demonstrate that burst analysis following the proper burst search (fixed count rate threshold) can provide information on PSF size and peak intensity consistent with information obtained by FCS analysis. Reciprocally, when comparing a series of samples, bursts analysis can be used to estimate changes in molecular properties such brightness and diffusion coefficient.

#### 4.2.4 Donor leakage factor

Because the data collected from each spot involves two separate SPADs, and the SPAD array alignment is a global process, it is possible that acceptor signal is collected with different efficiency in different spots. As a consequence, the donor leakage factor characterizing the amount of donor signal collected in the acceptor channel could vary across spots. Moreover, because occasional realignment was performed in between measurements, it is not even certain that the leakage coefficient was identical for all samples. For these reasons, we computed the donor leakage factor for each spot individually as described in Appendix 9 in S1 File. Since this estimation requires isolating the D-only subpopulation on the basis of the PR histogram, it was only performed with the 7d, 12d, 17d and D-only (DO) samples. It turns out that the leakage coefficient is small (3–4%), therefore applying a spot-specific correction is not critical. This analysis, however, serves as further characterization of spot uniformity.

**Fig 6:**
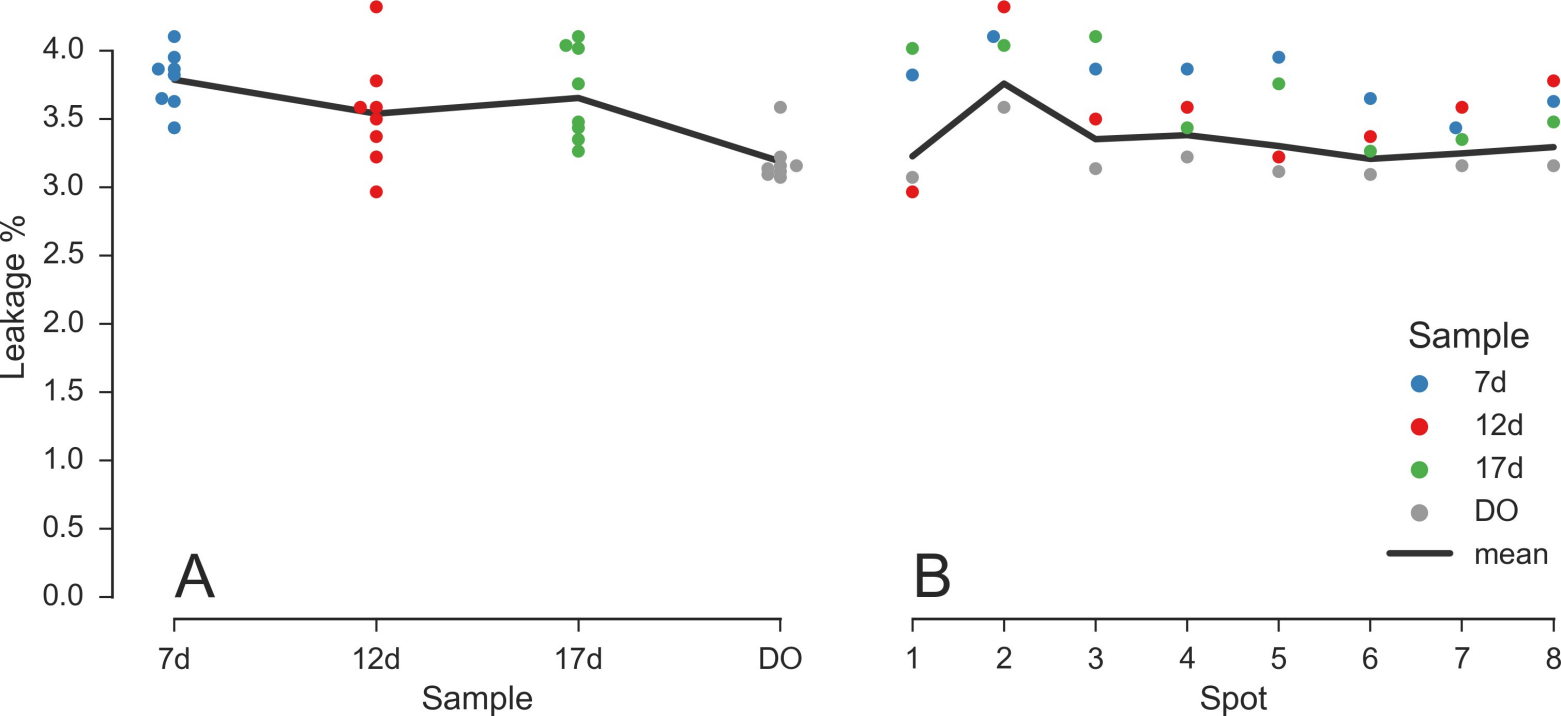
Leakage coefficient for different samples and spots. Leakage coefficient estimated for each spot for dsDNA samples 7d, 12d, 17d and DO. Each dot is the estimated leakage coefficient for a given spot and sample (the color indicates the sample). The left panel (A) shows the dependence of leakage versus sample, while the right panel (B) shows the dependence of leakage versus spot number. Black lines are weighted mean with weights proportional to the number of bursts detected in each sample (A) or spot (B). Both graphs show a good uniformity across samples and spots, with leakage factors in the 3–4% range. For computational details (including the numerical values used in this figure) see section *Leakage coefficient* (view online) of the accompanying Jupyter notebook.

Fig 6 compares the individual leakage coefficients for each spot and for each measurement (see also Tables SI-4 to SI-6 and Fig SI-17 in S1 File). In the left panel (A) the estimated leakage coefficients are grouped by sample while in the right panel (B) are grouped by spot number. The DO sample yields slightly lower values for all spots (Fig 6A), while spot 1 appears to have a larger leakage factor for all samples (Fig 6B). However, the absolute variation is of the order of 1% and results in negligible changes in the computed FRET efficiency *E* values. For this reason, we used a fixed value of *l* = 3.3% for all spots and samples in the remainder of the multispot analysis.

#### 4.2.5 Direct acceptor excitation factor

Due to the absence of an acceptor-specific laser excitation signal, the contribution to the acceptor signal due to direct excitation of the acceptor by the donor-excitation laser needs to be expressed as a function of the total (*γ*-corrected) burst size as explained in Appendix 9 in S1 File [30, 37]. The corresponding factor, *d*_*T*_, depends only on the dye pair and can be therefore estimated from μs-ALEX measurements (Section 3.6 and Appendix 9 in S1 File).

In this work, we found that the mean value of this factor was *d*_*T*_ = 4.9%. For computational details, see Section *Direct excitation*: *physical parameters* (view online) of the accompanying Jupyter notebook.

#### 4.2.6 Correction factor *γ*

As discussed in Appendix 9 in S1 File, the multispot correction factor *γ*_*m*_ is computed from the measured *PR* value of a sample, so that the *γ*-corrected FRET efficiency *E* of that sample matches the corresponding FRET efficiency measured in the single-spot μs-ALEX measurement (Eq. (SI.61)). This can in principle be done individually for each spot, and separately for each sample. Considering the low dispersion of the donor leakage correction factors (Section 4.2.4) and the good uniformity across spots of the FCS analysis results (Section 4.2.2), we only report here the average *γ*_*m*_ factor (and its standard deviation across spots) for each sample. Table 4 reports *γ*_*m*_ estimated using different *PR* analysis methods. The results for samples 22d and 27d are also shown, even though they are strongly affected by the overlap of the FRET and D-only *PR* distribution and are therefore unsuitable for *γ* factor estmation.

**Table 4:** Multispot γ factor. Line 1–3: multispot *γ_m_* values and their obtained from Table 5 and Fig 2, using Eq. (SI.61) with *PR* estimates obtained by Gaussian fit, KDE or SNA, respectively. Values computed for samples 22d and 27d (indicated in italics) are unreliable due to the overlap between DO and DA *PR* peaks. Last line: factor *K* (Eq. (SI.65)), proportional to the uncertainty on *γ_m_*, computed for the Gaussian fir approach. Values for the other methods are comparable. A common set of parameters *d_s_* = 0.061, *β_s_* = 0.81, *l_m_* = 0.033 was used, average of the values obtained with the various analysis methods.

	7d	12d	17d	22d	27d
Gaussian	0.44 ± 0.03	0.45 ± 0.04	0.46 ± 0.02	*0*.*37 ± 0*.*09*	*0*.*53 ± 0*.*08*
KDE	0.41 ± 0.03	0.39 ± 0.03	0.41 ± 0.05	*0*.*24 ± 0*.*09*	*0*.*54 ± 0*.*09*
SNA	0.41 ± 0.03	0.44 ± 0.02	0.43 ± 0.02	*0*.*29 ± 0*.*09*	*0*.*64 ± 0*.*20*
*K*	3.5	1.9	2.4	*4*.*6*	*8*.*5*

We observe a reasonable uniformity between the different estimates for samples 7d-17d. Error analysis reported in Appendix 9 in S1 File (Eq. (SI.65)) provides *K*, a scaling factor proportional to the uncertainty on *γ*_*m*_. The minimum *K* value is obtained for sample 12d, which was therefore used as reference in the remainder of this work (*γ*_*m*_ = 0.45).

#### 4.2.7 FRET results

The *PR* histograms of most spots and samples yielded two peaks, the lowest one corresponding to the donor-only (DO) population and the largest one to the doubly-labeled (DA) population. In the low FRET cases (sample 22d and 27d), only one peak could be resolved. Its location was used to estimate the *PR* value of the DA population. While this location is probably biased towards small values due to the presence of a residual DO population, the DO fraction observed in the corresponding samples studied on the single-spot μs-ALEX setup was below 10%. Gaussian fitting of the 22d and 27d data assuming an additional DO fraction (kept below 20%) did not significantly affect the peak position estimate (*ΔPR/PR* < 2%).

The results of Gaussian fit, KDE and SNA analysis of the different samples and spots are provided in Table SI-13 to SI-17 in S1 File. Analysis of the pooled data of all spots for each sample yielded results essentially identical to the average of all individual spots. The resulting proximity ratios are reported in Table 5.

**Table 5:** Fitted PR values. Mean and standard deviation of *PR* histogram peak values obtained using Gaussian fit, KDE or SNA analysis (SNA mean value).

	7d	12d	17d	22d	27d
Gaussian	0.85 ± 0.01	0.57 ± 0.02	0.29 ± 0.01	0.12 ± 0.02	0.10 ± 0.01
KDE	0.85 ± 0.01	0.55 ± 0.02	0.27 ± 0.02	0.09 ± 0.02	0.08 ± 0.01
SNA	0.82 ± 0.01	0.55 ± 0.02	0.28 ± 0.01	0.10 ± 0.02	0.08 ± 0.01

*PR* values from Table 5 were corrected using this value of *γ*_*m*_ and the values of *l* and *d’* obtained earlier, using Eq. (SI.57). They are compared to the previously obtained single-spot μs-ALEX values in the next section

### 4.3 Comparison of multispot and single-spot results

In this section, we compare the results obtained in the single-spot μs-ALEX measurements and those obtained with the multispot setup, and review the main differences between both measurements, and lessons to be drawn from their comparison.

#### 4.3.1 FRET efficiencies

**Fig 7:**
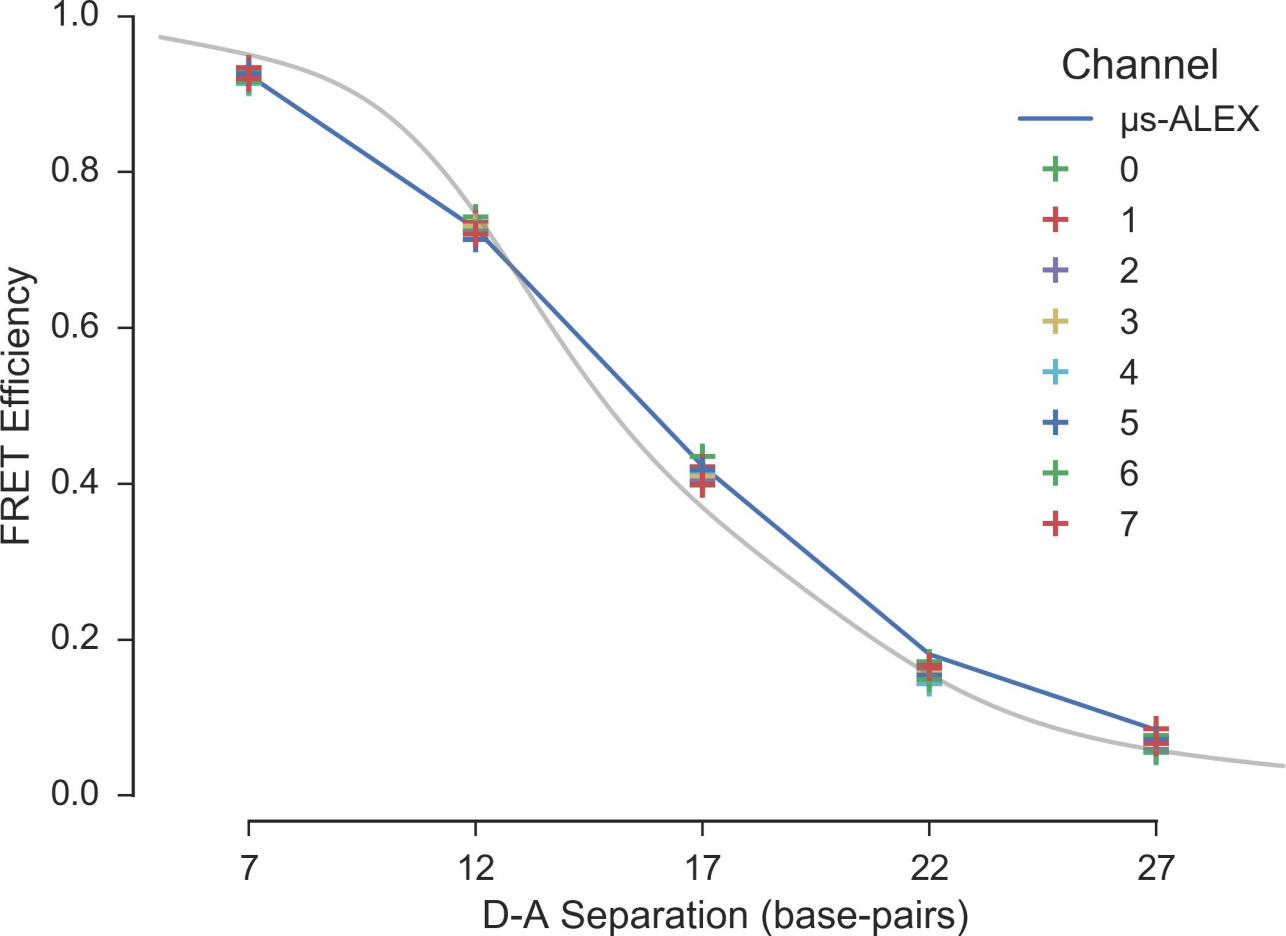
FRET efficiency versus dye separation in the multispot experiments. The *γ*-corrected FRET efficiency values obtained by Gaussian fit are represented as a function of dye separation (in base pair unit). Spots are indicated by different colors. The single-spot μs-ALEX results are represented as a connected line. The distance dependence of E predicted by a simple model of the DNA double helix, to which dyes are attached by a linker at a fixed position, is shown as a guide to the eye. The parameters used in the model are defined in Appendix 16 in S1 File. For further details see section *FRET vs distance* (view online) of the accompanying Jupyter notebook.

Fig 7 represents the corrected FRET efficiencies obtained in the previous section, together with the results of the single-spot μs-ALEX analysis (Section 4.1). The prediction of a simple DNA model (discussed in Appendix 16 in S1 File) is indicated as a guide for the eye.

The agreement between the two measurements is good, considering the impossibility to separate the contributions of donor-only and low FRET populations for sample 22d and 27d (resulting in an underestimation of the low FRET values in the multispot experiment), and the possible differences between spots in the multispot measurements. It is sufficient for studies concerned with identifying different populations more than extracting precise structural information.

For a visual comparison of FRET histograms obtained with single- and multispot setups on the 5 dsDNA samples see Appendix 9.4 in S1 File.

#### 4.3.2 Detection efficiency differences

The photon detection efficiency (PDE) of the SPAD arrays in the red region of the spectrum (acceptor emission) is about half lower than that of the SPADs used in the single-spot experiments (Fig SI-2 in S1 File). While the *γ*_*m*_ factor value of ~0.45 computed for the multispot setup (while *γ* ~ 1 for the single-spot setup) takes care of this difference when computing FRET efficiencies, too small a PDE could result in low detected counts and therefore, low signal-to-noise and signal-to-background ratios.

**Fig 8:**
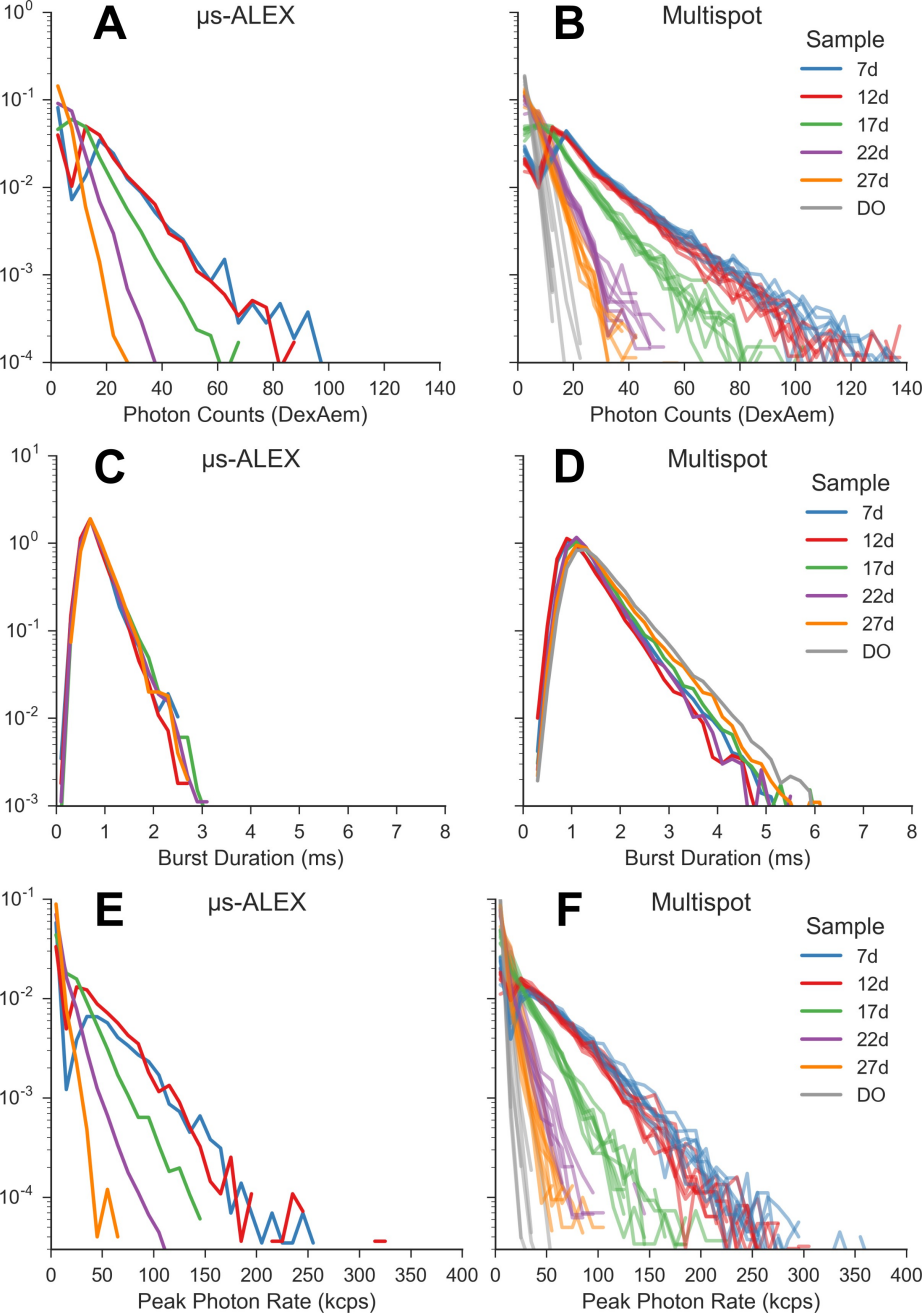
μs-ALEX vs multispot burst data distributions. Distributions of acceptor counts after donor excitation, burst duration and peak photon rate in each burst for both single-spot μs-ALEX (left column) and multispot (right column) experiments. Bursts were searched using the donor-excitation stream and a constant count rate threshold (*r*_*min*_ = 25 kHz for both single-spot and multi-spot measurements), followed by a selection with *γ*-corrected burst size ≥ 15. All the distributions (i.e. histograms) are normalized so that their integral is equal to 1. For the multispot setup, acceptor counts and peak photon rates distributions (first and last row) are reported for each channel separately; for readability, for the multispot burst duration distribution (second row, right), we report the mean across the channels. For more details see section *Burst statistics vs usALEX* accompanying Jupyter notebook (view online).

However, despite this lower PDE, the number of acceptor channel counts per burst (*n*_*DexAem*_) in the multispot measurements were about 30% larger than in the single-spot μs-ALEX measurements (Fig 8A and 8B). A first contributing factor to this surprisingly large acceptor signal is the longer burst duration observed in the multispot measurements (Fig 8C and 8D). This increased duration is confirmed by FCS measurements of diffusion times (Table 3 and Table SI-12 in S1 File), and discussed further in the next section.

Another contributing factor to the measured burst size is the excitation intensity. The peak intensity in a spot can be estimated from the measurement of the peak count rate in that spot. Fig 8E and 8F show the distribution of peak acceptor photon count rates during donor excitation (D_ex_A_em_) in both types of measurement. The single-spot results were corrected for the fact that the D_ex_ period represents only about half of the total period, while the multispot results are raw values, uncorrected for the difference in PDE between the two experiments. Because the observed peak count rates are comparable, but the PDE of the detectors used in the multispot experiments is about half that of the detector used in the single-spot experiment, we conclude that the effective acceptor excitation intensity in the multispot measurements was approximately double that of the single-spot measurements.

The fact that an increased excitation intensity was used to obtain similar acceptor burst counts in the multispot measurements has several potential drawbacks. First, due to the cumulated losses in the excitation path, the required output laser power is quite considerable, calling for expensive laser sources (the input laser power used in our measurements was ~400 mW). Secondly, since some amount of saturation due to triplet-state blinking was observed in the single-spot measurements with lower excitation intensities (Table SI-5 in S1 File), larger amounts of saturation were likely present in the multispot measurements. Finally, since a pulsed laser was used during multispot measurements, the probability to excite dyes into higher excited states was higher in these experiments, potentially accelerating photobleaching.

While these were not issues in these particular experiments, such a situation is detrimental and will benefit from detectors with better PDE.

#### 4.3.3 Setup characteristics

While the multispot setup is intended to be equivalent to multiple single-spot setups working in parallel, constraints related to the generation of an excitation spot array, and to the alignment of SPAD arrays, create imperfections whose signature can be detected. In particular, as discussed above, both FCS and burst duration analysis indicate significantly longer diffusion times in the multispot than in the single-spot measurements. This increase could be due to two different causes: differences in PSF dimension or saturation effects.

A wider excitation PSF is expected (and has been observed previously [26]) due to the reduced overfilling of the objective lens back aperture by each of the beamlets constituting the excitation pattern source. Moreover, optical aberrations affecting the illumination spots furthest away from the optical axis, could result in deformation of the corresponding excitation PSF [9]. This effect does not appear to be noticeable in the ACF measurements (Fig 4A & 4B), but could be responsible for some of the differences observed in the CCF apparent diffusion times (Fig 4C). This was in particular noticeable for sample 7d, which exhibits an increased apparent diffusion time toward the center of the pattern, or sample 22d, which is characterized by a small steady increase from one end of the pattern (spot 1) to the other (spot 8).

Saturation effects could not be directly characterized in the multispot measurements because of complications in the afterpulsing contribution to the ACFs (Appendix 12 in S1 File). However, as mentioned before, some saturation effect was detectable in the single-spot measurements, as demonstrated by the presence of a 10–15% fraction of triplet state blinking (Table SI-5 in S1 File). Since the mean excitation power used in the multispot measurements was about twice larger than in the single-spot measurements, it is plausible that an increased triplet state population was present in the multispot measurements. The use of pulsed excitation in the multispot versus μs alternated CW excitation in the single-spot measurement further complicates the comparison between both types of measurements [38]. Finally, saturation increases the apparent diffusion time obtained from FCS analysis [39], which could further contribute to the longer diffusion times measured in the multispot experiments.

In summary, while they did not affect the results after proper analysis, some of the observed differences between the single and multispot setups could in principle be reduced by using a CW laser source (to reduce saturation).

#### 4.3.4 Limitation of non-ALEX measurements

The absence of an acceptor excitation laser in the multispot experiment limited the amount of information which could be extracted from the measurements. In particular, correction factors had to be estimated in non-ideal ways:

- The *γ* correction factor had to be estimated from comparison of the proximity ratio obtained in one measurement (12d sample) with the accurate FRET efficiency value computed from the corresponding single-spot μs-ALEX measurement.
- The direct acceptor excitation correction had to be estimated from μs-ALEX measurements and expressed as a function of the corrected burst size (Eq. (SI.53) or (SI.55)).
- For low FRET sample measurements (22d, 27d), no independent estimation of the donor-leakage coefficient *l* could be performed, since the DO population could not be distinguished from the doubly-labeled one (and was probably imperfectly corrected for direct acceptor excitation).

In addition to these issues, proximity ratio estimations were affected by the difficulty (or impossibility for some samples) to isolate singly-labeled from doubly-labeled molecular bursts:

- For all samples, bursts corresponding to molecular transit during which the acceptor blinked or bleached (resulting in a proximity ratio between the DO value and the actual FRET population value) created a “bridge” between the donor-only PR peak and the FRET PR peak, affecting the accuracy with which the peak position could be determined (in particular using a Gaussian fit method, or the shot noise analysis approach).
- For low FRET samples, the overlap between the donor-only and doubly-labeled PR peaks, prevented determining the DO fraction and thus, correcting for its influence on the computed FRET efficiency of the doubly-labeled population.

While these limitations are important, we have shown that a quantitative analysis covering the whole range of FRET efficiencies can be performed with a careful analysis. In cases where analysis is limited to samples with medium to high FRET efficiency, and when no accurate measurement of the FRET efficiencies is needed, the current multispot setup can provide high throughput without overly complex analysis, as demonstrated in the next section.

## 5 RNAP promoter escape kinetics

### 5.1 Introduction

To illustrate the high-throughput capabilities of the multispot system, we studied the kinetics of DNA transcription by the RNA polymerase (RNAP) of *Escherichia coli* (*E*. *coli*) in a reconstituted *in vitro* assay. RNAP carries the task of DNA transcription, which can be divided into three major steps:

1. Initiation, during which RNAP identifies and binds the promoter sequence of a gene and prepares RNAP for RNA polymerization activity;
2. Elongation, during which RNAP escapes from the promoter sequence and rapidly processes downstream DNA and polymerizes RNA;
3. Termination, during which RNAP identifies a stop signal and terminates the RNA polymerization process.

The strong interaction between the promoter sequence and the *σ* promoter-specificity factor of the RNAP complex [40–42] delays the transcription process and makes transcription initiation the rate limiting step of DNA transcription in many genes [43–45].

**Fig 9:**
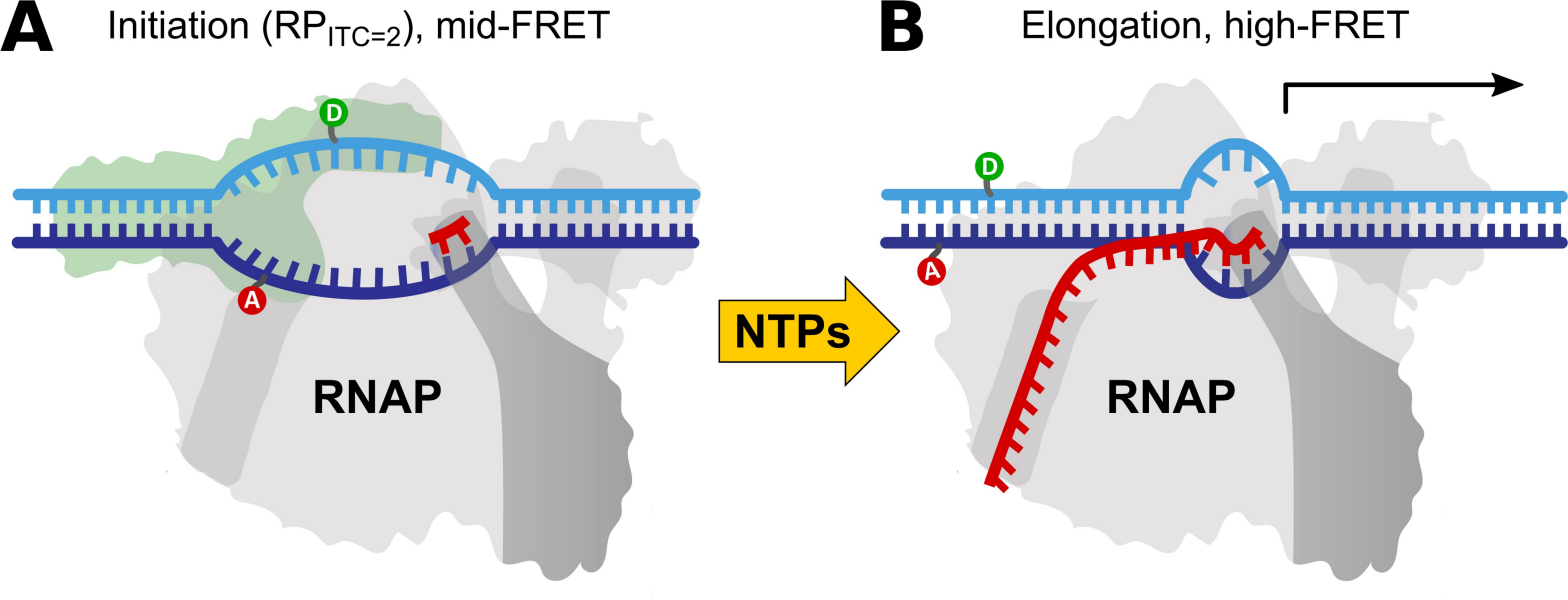
Schematic of the reaction observed in real time with the 8-spot smFRET setup. (**A**) The RNAP-promoter initially transcribed complex (RP_ITC_) is prepared with an initiating dinucleotide (red “π” symbol) as the nascent RNA chain. Complementary DNA strands are labeled at DNA promoter bases with donor (D, green, position -5) and acceptor (A, red, position -8) dyes. After formation of a transcription initiation bubble, the dyes are separated, resulting in medium FRET. The initial state remains in stationary conditions until the addition of the four missing nucleotides (NTPs, yellow arrow), which triggers transcription initiation and elongation. (**B**) During elongation, the transcriptional bubble moves downstream (to the right), causing hybridization of the sequence of the initial transcriptional bubble and a corresponding decrease of the D-A distance (FRET increase).

In *in vitro* transcription assays, the system starts in an initial state comprised of a stable RNAP-promoter open complex, in which a ~13 bases transcription bubble is formed by melting the promoter sequence between promoter sequence positions -11 and +2 (where +1 is the transcription start-site). In the *in vitro* assay, the open bubble is further stabilized by a dinucleotide to form an initially transcribed complex with a 2 bases nascent RNA (RP_ITC = 2_, Fig 9A). The final state (elongation and run-off, Fig 9B) is reached after RNAP escapes from the promoter sequence and starts transcribing the sequence downstream from the promoter.

The onset of the transcription reaction is triggered by the addition of all four nucleoside triphosphates (NTPs: ATP, TTP, GTP, and CTP). Direct measurement of the kinetic transition from RP_ITC = 2_ to run-off on freely-diffusing smFRET setups is difficult due to the small number of bursts available for population analysis over the short time scale of the kinetics (< 3 min). Here, we report a real-time measurement of such kinetics at the single-molecule level, which, to the best of our knowledge, was never measured before on non-immobilized molecules. Exploiting the higher throughput of the 8-spot smFRET system, we recorded the kinetics of promoter escape from initiation into elongation in the *E*. *Coli* lacCONS promoter [46–48]. Details of the sample preparation can be found in Lerner, Chung *et al*. [49].

### 5.2 Principle of the experiment

The change in DNA conformation during the reaction is monitored by labeling the two complementary DNA strands with fluorescent probes (donor: ATTO550 and acceptor: ATTO647N). The dyes are positioned within the transcriptional bubble opened in the RP_ITC = 2_ state, as shown in Fig 9. During the transition into elongation, the RNAP moves downstream with the transcription bubble, causing re-annealing of the upstream DNA strand and a corresponding melting of downstream template DNA. Re-annealing of the template DNA causes reduction in the donor-acceptor distance and increase in FRET efficiency. With this labeling configuration, we obtain a medium-FRET population (*PR* = 0.62) for RP_ITC = 2_, and a high-FRET population (*PR* = 0.952) for the final state. The medium-FRET population is well separated from the donor-only peak, mitigating the limitation of the single laser excitation configuration of our 8-spot setup.

The measurement is performed during a continuous acquisition comprised of 3 phases:

- Phase 1 (10–15 min): 100 μl of the sample in the RP_ITC = 2_ state is placed on the setup and measured in order to acquire an accurate representation of the initial FRET efficiency histogram.
- Phase 2 (<20 s): without stopping the acquisition, 2 μl of NTPs are added with a manual pipette into the sample, in order to reach a final concentration of 100 μM of NTPs. Then, we immediately pipette up and down 30 μl of sample solution for a few seconds to ensure mixing in less than 20 s. Moreover, even though the entire volume is not perfectly mixed, the high NTP concentration allows a quick start of the reaction as along as a small fraction of NTP reaches the excitation volume.
- Phase 3 (30–45 min): the acquisition continues until an asymptotic steady state is reached.

### 5.3 Data analysis

Burst search was performed on the full data set, in windows of constant durations, selecting bursts containing more than 30 counts after background correction (*n*_*Dex*_ ≥ 30). Since the experiment’s purpose was to monitor the transition from one state (medium FRET) to a very distinct one (high FRET), there was no need for *γ-* or other corrections. *PR* distributions exhibited three distinct peaks: DO, medium-FRET (open complex in RP_ITC = 2_ state) and high-FRET (hybridized DNA), which were fitted with 3 Gaussians having constant peak positions and widths, while amplitudes were allowed to vary as a function of time.

Two pre- and post-kinetics steady-state regimes (10–15 min long) in the measurement were identified and used to fit the initial and final *PR* histograms. These initial and final *PR* peak positions and widths, were used throughout the whole measurement, the only adjustable parameters being the respective fraction of each peak.

**Fig 10:**
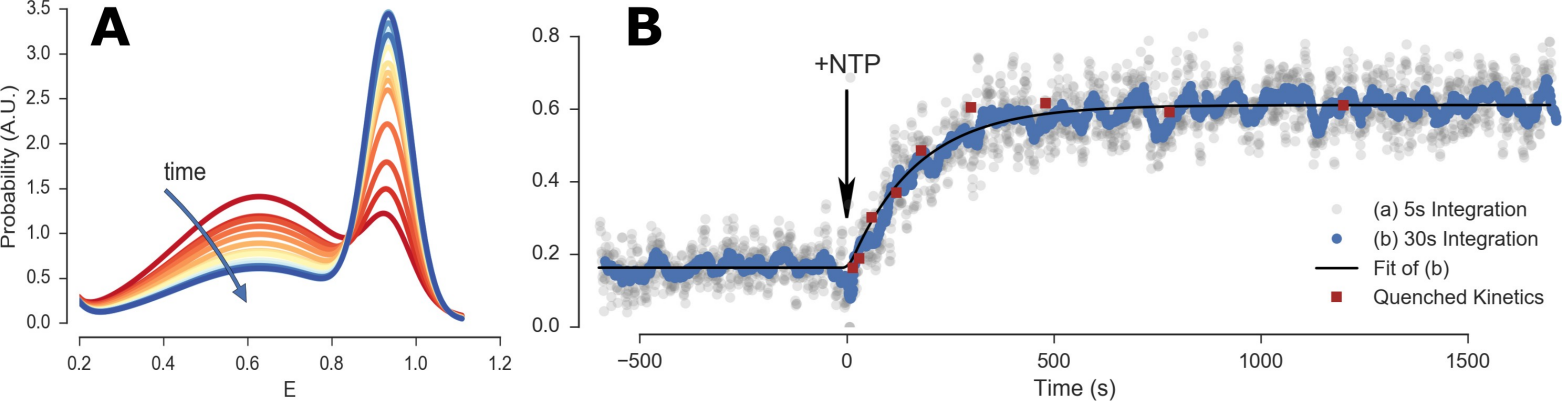
Real time transcriptional bubble closure kinetics results. (A) Evolution of FRET efficiency distribution as function of time (one curve per 30 s). The curves represent Gaussian fit of the FRET histograms. (B) Fraction of high FRET population obtained in the real time kinetics measurement (grey and blue dots) and fraction of probe hybridization to a run-off transcript from quenched kinetics assays (red squares). Dots are computed as a function of time using either a 5 s (grey) or 30 s (blue) moving integration window. The solid black curve is a single-exponential model fitted to the 30 s moving integration window. Quenched kinetics data (from ref. [49]) are normalized to fit initial and final values of real time kinetics trajectory. For more details on the analysis see accompanying *Realtime Kinetics Analysis* Jupyter notebook (view online).

To compute a kinetic curve, we selected bursts within sliding integration windows (duration: 5 or 30 s, step: 1 s). For each window (*i*.*e*. time point in the kinetics), the amplitudes of the 3-Gaussian model were fitted (Fig 10A). The ratio of fitted component fractions (high-FRET / (mid-FRET + high-FRET)) was then represented as a function of time (Fig 10B). A first order kinetic time constant *τ* was fitted using an exponential model incorporating the effect of the integration window. Monte Carlo simulation of simulated kinetic curves with additive Gaussian noise (with variance equal to that observed in the experimental trajectories) were used to estimate the uncertainty on the kinetic time constant for different values of *τ*. Simulations show that time constants as small as 10 s can be reliably measured using this approach (*σ*_*τ*_ = 2.3 s).

### 5.4 Results

Fig 10 shows the measured evolution of the high FRET fraction in a single continuous acquisition. Time zero represents the time of NTP injection (Section 5.2) and marks the start of the reaction (RP_ITC = 2_ to elongation, Fig 9). After injection and mixing, the reaction starts with a short delay (< 20 s), due to the time needed for NTPs to reach the excitation volume by diffusion and convection. Data processed using sliding windows of 5 or 30 s illustrate the necessary trade-off between accuracy and time resolution. In this particular case, fit of the 30 s resolution curve was sufficient and led to first order kinetic time constant *τ* = 172 ± 17 s (mean and standard deviation of 3 measurements).

Fig 10B also shows data points from a series of quenched kinetics experiments red square), obtained with the same system but a completely different experimental strategy (see ref. [49] for details). In this approach, a doubly-labeled complementary ssDNA probe hybridizes with the RNA transcript once the transcription reaction is stopped after a specified reaction time *t* (the probe is a 20dT oligo labeled at the 3' and 5' termini with Tetramethylrhodamine and Alexa Fluor 647 respectively). Hybridization of the probe results in a shift from high to low FRET. As in the experiment reported here, the reaction is started from a RP_ITC = 2_ state by addition of NTPs, but it is then stopped (quenched) after a fixed time *t*, by addition of 0.5 M Guanidine Hydrochloride (GndHCl). The sample is then incubated for 30 min for hybridization with the ssDNA probe to occur, and finally measured on a single-spot μs ALEX setup for 10–20 min. The procedure is repeated for different time points (reaction times), yielding the data points (red squares) reported in Fig 10B.

The remarkable agreement between the two experimental approaches used to probe transcription: (i) quenched kinetics experiments probing the transcript production and (ii) real-time kinetics experiments, probing the change in conformation upon promoter escape, demonstrates the advantages of a multispot approach for tracking kinetics in real-time. From a biochemical point of view, the agreement between the two different assays indicates that the transcript production rate in quenched kinetics experiments is limited by the initiation part of the reaction, which is the only part probed in realtime kinetics experiments. This result is in accordance with previously published works which measured the high processivity of RNAP in elongation (~10ms per base) [50] and identitified initiation as the rate-limiting step for transcript production [3, 43–45, 50–53].

## 6 Conclusion and perspectives

We have presented a detailed description of an 8-spot confocal setup and illustrated its use for smFRET studies with the following examples. First, measurements of a series of freely diffusing doubly-labeled dsDNA samples demonstrated that data simultaneously acquired in different spots could be properly corrected and analyzed in parallel, resulting in measured sample characteristic identical to those obtained with a standard single-spot μs-ALEX setup. We discussed the advantages of a multispot setup, while pointing potential limitations of the current single laser excitation design, as well as analysis challenges and their solutions. Second, we leveraged the increased throughput provided by parallel acquisition to address an outstanding question in the field of bacterial RNA transcription. We showed that real-time kinetic analysis of promoter escape by bacterial RNA polymerase confirmed results obtained by a more indirect route, shedding additional light on the initial steps of transcription.

The concepts discussed here are relevant to future generations of multispot setups, including those using multicolor μs-ALEX or ns-ALEX/PIE excitation schemes [54, 55]. Freely diffusing smFRET and other single-molecule fluorescence applications will directly benefit from one to two orders of magnitude throughput improvements afforded by the development of custom-technology SPAD arrays with enhanced sensitivity in the red region of the spectrum [56, 57], or larger number of detectors and different geometries [16].

Progresses making this technology more accessible can be anticipated in different areas. Improvements to the LCOS-SLM-based excitation scheme presented here can lead to a much better utilization of the laser output power. While the current approach provides flexibility in designing the illumination pattern geometry and helps with alignment, simpler and cheaper alternatives can be envisioned.

Efficient, parallel and user-friendly analysis tools will be needed to handle the large amount of data generated by these types of measurements. An open source and rigorous approach to algorithm development will be indispensable to allow cross-validation of massive data sets acquired on different setups across research groups [18].

Finally, coupling this type of setup with other technologies such as microfluidics [58, 59] is poised to transform single-molecule analysis from a niche technology limited to research laboratories to a mainstream and powerful tool with applications in diagnostics and screening [46].

## Supporting information

S1 File Supporting Information for the manuscript “Multispot single-molecule FRET: towards high-throughput analysis of freely diffusing molecules”. (PDF)
