# Supplementary material for: Multispot single-molecule FRET: High-throughput analysis of freely diffusing molecules
Source: PLoS One. 2017 Apr 18;12(4):e0175766. doi: 10.1371/journal.pone.0175766 (PMC5395192; doi:10.1371/journal.pone.0175766)
Supplement: S1 File — (PDF) [file pone.0175766.s001.pdf]

Supporting Information for
“Multispot single-molecule FRET: high-throughput analysis of freely diffusing molecules”

A. Ingargiola[1,†], E. Lerner[1], S. Chung[1], F. Panzeri[2], A. Gulinatti[2], I. Rech[2], M. Ghioni[2], S. Weiss[1], X. Michalet[1,‡]

† tritemio@gmail.com

‡ michalet@chem.ucla.edu

1. Department of Chemistry & Biochemistry, UCLA, Los Angeles, CA

2. Dipartimento di Elettronica, Informazione e Bioingeneria, Politecnico di Milano, Milan, Italy

Contents

Appendix 1 Data Files used in this work 5
1.1 Photon-HDF5 Files 5
1.2 Other Files 5

Appendix 2 Software 5
2.1 Jupyter notebooks 5
2.2 ALiX Scripts 6

Appendix 3 Setups and Acquisition Hardware 6
3.1 Single-Spot µs-ALEX Setup 6
3.1.1 Setup description 6
3.1.2 Data acquisition 8
3.2 Multispot setup 8
3.2.1 Optics 8
3.2.2 Background suppression 9
3.2.3 Detection path 10
3.2.4 Multispot data acquisition 10
3.2.5 Alignment 10

Appendix 4 Crosstalk & Afterpulsing Analysis 14
4.1 Crosstalk 14
4.2 Afterpulsing 16

Appendix 5 Photon Streams Definition 16

Appendix 6 Background Rate Calculation 18
6.1 Introduction 18
6.1.1 *Sources of background 18*
6.1.2 Background rate calculation for each measurement 18
6.1.3 Inter-photon distribution: 18
6.2 Variable background rates. 20
6.3 Special consideration for µs-ALEX 22

Appendix 7 Details on Burst Search 23
7.1 Photon Streams 23
7.2 Burst Threshold 24
7.2.1 Standard Definition of the Burst Threshold 24
7.2.2 Other Definitions of the Count Rate 24
7.3 Burst Fusion 25
7.4 Burst Search Influence on Burst Statistics 25

Appendix 8 PR and SR Histograms 30
8.1 Standard Histograms 30
8.2 Weighted PR Histogram 31

Appendix 9 Correction Factors 36
9.1 Theory 36
9.1.1 Donor Leakage Factor 36
9.1.2 Acceptor Direct Excitation Factor 36

9.1.3 The γ Factor *37*
9.2 Single-Spot μs-ALEX Experiments 37
9.2.1 Donor Leakage Factor *37*
9.2.2 Acceptor Direct Excitation Factor *38*
9.2.3 γ Factor *38*
9.3 Multispot Experiments 38
9.3.1 Donor Leakage Factors *38*
9.3.2 Acceptor Direct Excitation Factor(s) *40*
9.3.3 γ Factor *42*
9.3.4 Error on factor γ estimation *42*
9.3.5 Proximity Ratios *43*
9.4 Multispot vs single-spot FRET histogram comparison 44
Appendix 10 Determination of Proximity Ratio and FRET Efficiency 46
Appendix 11 Shot Noise Analysis 47
11.1.1 Using the acceptor excitation information in μs-ALEX measurements in SNA *48*
11.1.2 Algorithm for shot noise analysis in multispot measurements *49*
11.1.3 Normal FRET efficiency distribution model *49*
11.1.4 Beta distribution of FRET efficiencies *50*
11.1.5 Fuzzy dumbbell model of distance distribution *50*
Appendix 12 FCS Analysis 52
12.1 Single-Spot μs-ALEX FCS 53
12.2 Multispot FCS 54
12.3 Single-spot FCS Results 56
12.4 Multispot FCS Analysis Results 58
Appendix 13 Samples Description 60
13.1 dsDNA FRET Samples 60
13.2 RNAP Transcription Samples 60
Appendix 14 Single-Spot Setup Characterization 61
14.1 Mean Count Rates and Background Rates 61
14.2 Peak Burst Count Rate 62
14.3 Burst Rate *n(t)* 62
14.4 FCS analysis 63
Appendix 15 Burst Statistics Definitions 63
15.1 Peak Burst Count Rate 63
15.2 Count Rate in the Presence of Laser Alternation 64
15.3 Proxy for the Maximum Peak Count Rate 65
15.4 *γ*-Corrected Burst Size 68
15.5 SBR & SNR 70
15.6 Burst rate *n(t)* 73
Appendix 16 Simple model of the DNA double-helix with two labels 74
16.1 Model description 74
16.2 Model parameterization 76
Appendix 17 RNAP Escape Kinetic Experiments 77

Appendix 18 References 78

Convention used in this document: underlined text is associated with a clickable web link. To access the linked document, follow the instructions for the PDF reader you are using (generally, Ctrl+click on the link will open the default browser at the specified address).
Contact the authors if you encounter any problems with the links or documents.

Appendix 1 Data Files used in this work

Original data files as well as accessory files useful during data analysis have been deposited on Figshare and given a digital object identified (DOI), which can therefore be used for retrieval and citation. Direct links are provided below.

1.1 Photon-HDF5 Files

Photon data files, saved in the Photon-HDF5 format (.hdf5 extension) [1], can be downloaded using the following DOI/links:

- Single-spot µs-ALEX Data Files [2]: https://doi.org/10.6084/m9.figshare.1098961
- Multispot Data Files [3]: https://doi.org/10.6084/m9.figshare.1098962
- RNAP Promoter Escape Kinetics Data Files [4]: https://dx.doi.org/10.6084/m9.figshare.3810930

These files can be analysed using FRETBursts, ALiX or any other single-molecule data analysis software supporting the Photon-HDF5 format described in ref. [1].

1.2 Other Files

Files used to correct the single-spot µs-ALEX autocorrelation function for afterpulsing [5] can be downloaded using the following DOI/link: https://doi.org/10.6084/m9.figshare.3817062.
Files used to compute the afterpulsing probabilities of SPAD arrays [6] (cf Section 4.2) can be downloaded using the following DOI/link: https://doi.org/10.6084/m9.figshare.4784572.

Appendix 2 Software

2.1 Jupyter notebooks

Installing FRETBursts. In order to execute the notebooks, you need to install FRETBursts first. If you have already installed python through conda just type:

```
conda install fretbursts -c conda-forge
```

Otherwise, see the instruction on the FRETBursts manual.

Downloading the notebooks. The multispot_paper github repository [7] contains all the note-books and results produced for this paper. We suggest to download the ZIP archive from this link. The archive is ~110 MB and contains all the output of the processing (figures and numeric results such as bursts data, fitted parameters, etc.).

Downloading the data. Download the dsDNA datasets for single-spot µs-ALEX (link) and for multispot smFRET (link) and put it in the folder *data/singlespot* and *data/multispot* respectively. For the realtime kinetics experiments, download the data files (link) and place them in the folder *realtime kinetics/data/*.

Reproducibility. For expert python users, the notebook archive contains a conda environment file that can be used to recreate the exact environment (i.e. the exact version of each library) used during the preparation of this paper.

Using the notebooks. The main notebook, index.ipynb (in the root folder of the archive), contains links to all the other notebooks used for the analysis (with a brief explanation of what each notebook does). It also contains links to output data files in CSV format (stored in the results subfolder). The index notebook can also be used to re-execute all the notebooks in a single step and recompute all the paper’s results and figures from scratch. The notebooks for realtime kinetics analysis can be found in the realtime kinetics subfolder of the archive. Therein, the notebook index_realtime_kinetics.ipynb links to all the notebooks of the realtime kinetics analysis.

2.2 ALiX Scripts

ALiX is a standalone Microsoft Windows 64 bit standalone application which can be downloaded from its public website (https://sites.google.com/a/g.ucla.edu/alix/), by visiting the Installation webpage for detailed instructions. The website contains an extensive online manual including tutorials. Among the many features of the software is the ability to save settings as well as a list of analysis steps into a “script” text file, which can be reloaded later on to replicate the analysis (or follow the same analysis steps with a different data file). Instruction on how to load and execute a script can be found in the corresponding page of the online manual (General Multi-spot Analysis).

These scripts have been deposited on Figshare [8] and can be downloaded using the following DOI/link: https://doi.org/10.6084/m9.figshare.3839427. The repository contains a readme.txt file which describes all scripts and the associated data files.

Appendix 3 Setups and Acquisition Hardware

3.1 Single-Spot μs-ALEX Setup

3.1.1 Setup description

The setup used in this work was previously described in ref. [9]. The setup schematic is reproduced in Fig. SI-1.

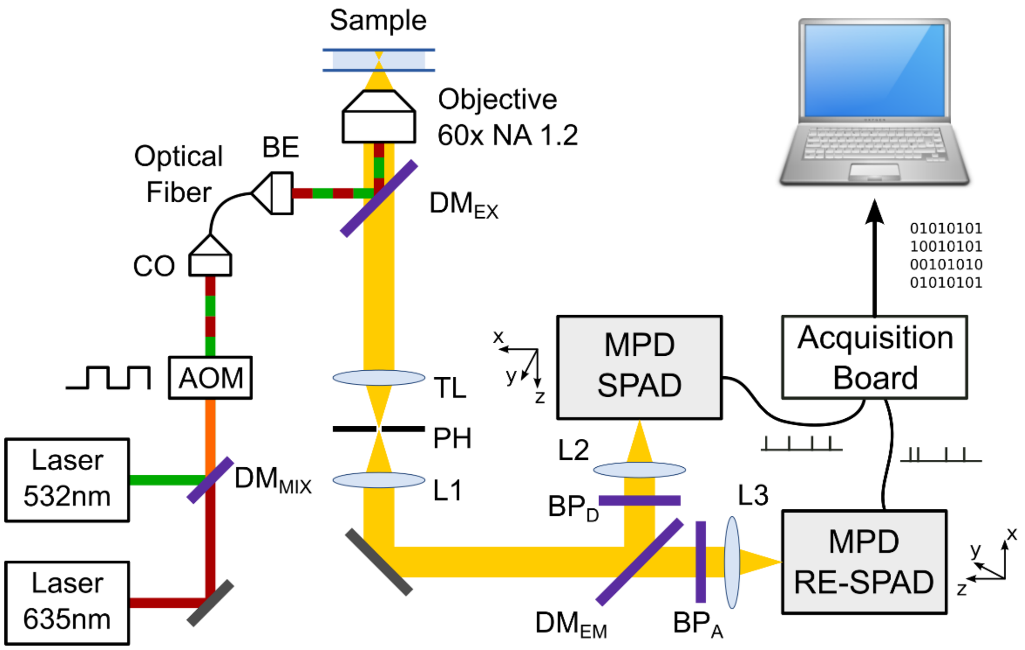

Fig. SI-1: Single-spot µs-ALEX setup. Two CW lasers are alternated using a computer controlled acousto-optic modulator (AOM). The beams are then coupled to a single-mode fiber whose output is expanded using a beam expander (BE) before coupling to the objective lens via a dichroic mirror (DMex). The emitted fluorescence is collected through the objective lens and dichroic mirror and refocused onto a 100 µm pinhole (PH) between the microscope tube lens (TL) and a recollimating lens L1. The two channels (green: D, red: A) are separated using a dichroic mirror (DMem) and focused onto the RE-SPAD using a 75 mm focal length lens (L2 or L3). The TTL output from each SPAD are sent to a counting board (NI-6602) installed in a PC running the LabVIEW acquisition software.

The PDEs of the two types of detectors used in this study are represented in Fig. SI-2.

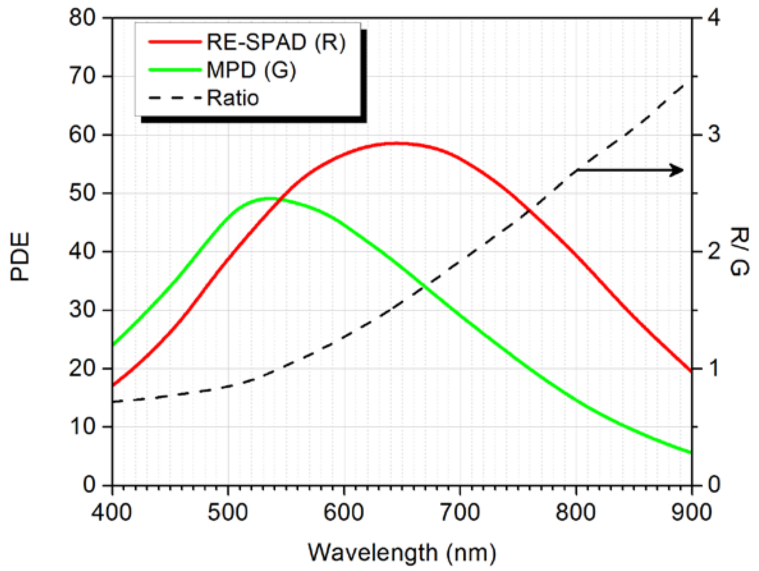

Fig. SI-2: **Photon detection efficiency (PDE) of the two types of detectors used in this study**. In the single-spot µs-ALEX measurements, a standard technology SPAD from MPD (green) was used for the donor channel, while a red-enhanced SPAD detector from the Polimi group (red) was used for the acceptor channel. The multispot experiments used SPAD arrays manufactured with the same technology as the MPD SPAD and are therefore characterized by the same PDE curve. The ratio of the two PDEs is represented in black (left axis, R/G). The spectral range of the emission bandpass filters (donor: green, acceptor: red) use in the single-spot µs-ALEX measurements are indicated as rectangles on the graph.

3.1.2 Data acquisition

A single digital input/output board (NI PCI-6602, National Instruments, Austin, TX) was programmed using LabVIEW 7.1 (National Instruments) to:

(i) send TTL signals (+2.4 V) to each line of the AOM controller, the “on” (resp. “off”) period of each laser alternation corresponding to the +2.4 V (resp. 0 V) state of the corresponding line,

(ii) detect and time stamp TTL pulses emitted by each SPAD (one time counter per channel).

TTL inputs and outputs were recorded and generated using a single on-board 80 MHz clock, guaranteeing synchronization of the 12.5 ns resolution time stamps with laser alternation. Each time stamp was recorded with its detector number, and the stream of time stamps recorded in a proprietary binary format (.sm files). Files were converted into the open source HDF5 photon data format (.hdf5 files) [1] and are available as online at URLs indicated in the references [2].

Single spot μs-ALEX measurements for each of the five samples were performed sequentially on a distinct setup from that used for multiple spot measurements. Performing measurements sequentially minimized the possibility that the setup characteristics change from one measurement to another. However, since the measurements were performed on a different day than the multispot measurements, concentration of the samples could be different in the μs-ALEX and multispot measurements.

3.2 Multispot setup

A general schematic overview of the setup can be found in Fig. SI-3.

3.2.1 Optics

The 8-spot excitation pattern was generated using a 1 W, 532 nm pulsed laser (IC-532-1000 ps, High Q Laser Production GmbH, Hoheneims, Austria). After laser beam expansion and polarization adjustment, the beam reflects off an LCOS spatial light modulator (LCOS-SLM, model X10468-01, Hamamatsu Corp., Bridgewater, NJ, USA), which generates 8 separate spots on a focal plane a few centimeters from the LCOS surface. The LCOS is a programmable phase modulator, which can impose an arbitrary phase pattern to the incident linearly polarized plane wave (see details below, Section 3.2.5). In the 8-spot setup, the phase pattern creates the same phase modulation as would an array of small lenses (or lenslet array). Whereas a lens imposes a phase difference by putting different amount of material with specific refraction index in the light path (*i.e.* more material in the center of the lens, less at the edges), the LCOS SLM achieves the same result by interposing a thin layer of liquid crystals in the light path, whose light retardation can be controlled by an electric field (electro-optics effect) [10]. By designing a pattern of phase delays similar to that created by a Fresnel lens, the net result is to focus the light incident on that patch of LCOS SLM at a distance (focal length) specified by the pattern (32 mm in our case). Once formed, this pattern of focused beamlets can be manipulated by far-field optics just as any other light pattern.

A recollimating lens L3 (*f* = 200 mm, AC508-200-A, Thorlabs Inc., Newton, NJ, USA) collimates the 8 LCOS-focused spots, directing them toward the objective lens through a dichroic mirror. The unmodulated light, (mostly light incident on the LCOS outside the multispot pattern) is focused at the focal point of L3, where it is stopped by a background-suppressing spatial filter. A 60X water-immersion objective lens (numeric aperture, NA = 1.2, Olympus, Piscataway, NJ) focuses the spots into the sample through a microscope coverslip. The (*x*, *y*) position, orientation and pitch of the spots can be adjusted by changing the modulation pattern on the LCOS device. A linear arrangement of 8 excitation spots was generated in order to match the linear 8-pixel geometry of the detector.

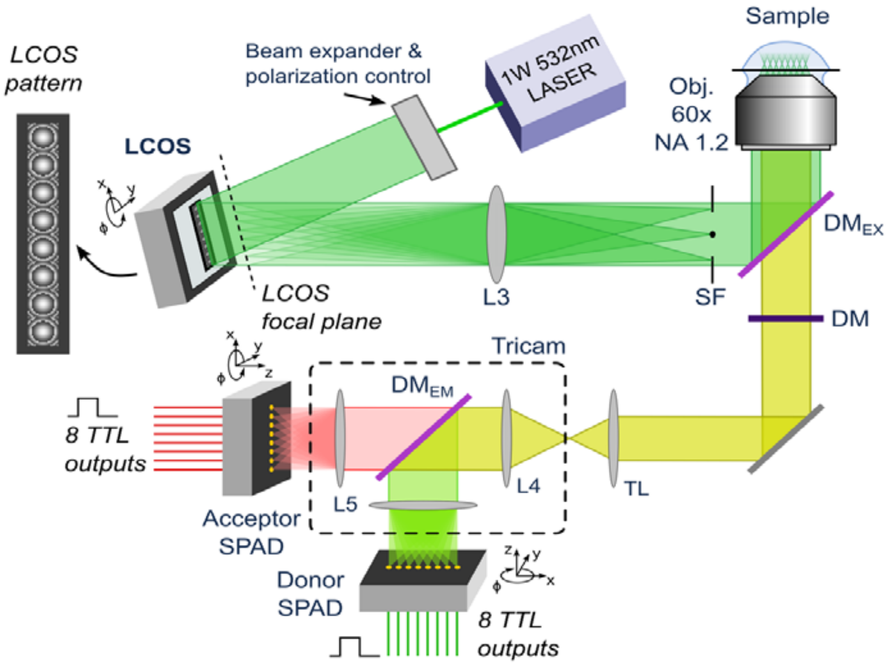

Fig. SI-3: Schematic of the 8-spot single-molecule FRET setup. A freely-diffusing sample is probed via 8 independent excitation spots generated by a 532nm CW laser and an LCOS spatial-light modulator. The fluorescent signal from each excitation spot is optically conjugated to a pair of pixels in the two detectors. The donor and acceptor emission is separated in two distinct spectral bands and detected by two separated SPAD arrays.

3.2.2 Background suppression

The background-suppressing spatial filter used here is an improved version of the pin-dot pattern reported previously [11, 12]. In the original design any light outside the multispot pattern, as well as the small fraction (<10 %) of unmodulated light incident on the multispot pattern, is simply reflected off the LCOS surface. If not blocked, this light generates unacceptable background levels (unfocused sample excitation). The spatial filter is placed at the focal plane of the L3 lens to block this unmodulated light. The pin-dot is a critical component which needs to block a continuous flow of high-power laser and can be easily damaged. Moreover, minimal alignment errors both in the X, Y and Z direction can cause significant increase in background and deterioration of the multispot pattern. To improve this pin-dot design, we use a linear Bragg grating pattern on all the LCOS area outside the multispot pattern.

The Bragg pattern creates a series of diffraction spots on the L3 focal plane. The 0-th order diffraction lays on the optical axis while the higher orders are offset in a direction orthogonal to the Bragg pattern. Using the highest spatial frequency achievable with the LCOS (one line with

phase 0 and one with phase π), more than 90% of the background light is steered away from the optical axis, into the first and higher diffraction orders. The higher orders are blocked in the L3 focal plane by two narrows stripes of absorbing material. This design achieves better background suppression than the pin-dot-only version and improve the long-term reliability of the pin-dot.

3.2.3 Detection path

Fluorescence emission generated by molecules diffusing through the 8 excitation spots was collected by the objective lens. A dichroic mirror separated the scattered laser light from the dyes emission (Fig. SI-3). An additional long pass filter was inserted to eliminate any residual fraction of scattered laser light transmitted by the dichroic mirror. The 8–spot emission signal was focused by the microscope tube lens and relayed by a multi-camera port system (Tricam, Cairn Research Ltd., Kent, UK) to two 8-pixel SPAD modules [13], after spectral separation. Each module was individually aligned so that each pixel's active-area received the emission signal from one of the conjugated excitation spots (Fig. SI-3).

The arrays geometry consisted of eight 50 µm-diameter pixels linearly distributed with a 250 µm pitch. The arrays, which have been described previously [11, 13], use a custom process developed by the Polimi group. This process allows obtaining arrays with low dark-counting rates (DCR) and higher PDE than arrays manufactured using CMOS processes (see for instance ref. [14, 15]). However, compared to the thick SPAD process (the detector technology used by the SPCM-AQR detectors of Excelitas or τ-SPAD detectors of Laser Components), the PDE of the custom arrays is about two-fold lower in the red part of the visible spectrum (>600 nm). Therefore, we expected a low sensitivity of the multispot system for low-FRET populations, characterized by very low photon counts in the red part of the spectrum (*i.e.* in the acceptor channel). A more detailed comparison of the different SPAD technologies mentioned above can be found in ref. [16].

3.2.4 Multispot data acquisition

Data acquisition from the two SPAD arrays was performed as described in ref. [17]. Briefly, each SPAD's TTL output was connected to a single digital input channel of a programmable board (PXI-7813R, National Instruments). The LabVIEW FPGA firmware uploaded on the board detects and time-stamps each signal with 12.5 ns resolution and associates it with its channel number, before transferring the data to a host computer running the LabVIEW acquisition software. Data was saved in binary format recording the time stamp and channel number of each photon in the order they were received, and displayed in real time as time traces of all channels for monitoring purposes. Raw data files were later converted in the Photon-HDF5 data format using the phconvert Python script [1]. These file are available online at URLs indicated Appendix 1.

3.2.5 Alignment

3.2.5.1 Excitation Path

When building the setup, before placing the L3 lens and the pindot filter, the laser needs to be aligned as in a standard confocal setup, using the LCOS as a mirror (the LCOS must be turned on and displaying a uniform black pattern). The expanded beam needs to be centered and aligned along the objective lens' optical axis. As in standard confocal systems, this alignment can be achieve using a pair of mirrors which are enough to provide both tilting and lateral translation of the beam. The LCOS position should be aligned so that the multispot pattern would lay as close

as possible to the peak of the Gaussian beam profile. One the beam is aligned the lens L3 can be inserted. This lens can be easily centered making sure that the confocal spot generated by the objective lens is in the same position after the L3 lens insertion. Finally, the pindot filter can be inserted at a focal-length distance after L3. In this configuration, when the pindot is aligned, it should block the laser excitation almost entirely. For fine alignment of the pindot, we suggest to use a high-concentration dye sample (e.g. 100 nM Cy3B solution) and a camera. Focusing several a few tens of microns inside the sample, it is possible to finely align the pindot by minimizing the emission intensity recorded by the camera. After this point, the multispot LCOS pattern can be activated. The same high concentration dye sample should show the multispot emission pattern. In principle is possible to translate the LCOS pattern to align the emission on the detector. However, this would bring the pattern away from the peak of the beam profile and away from L3 optical axis, causing uneven attenuations and aberrations. Therefore, it is better to align the SPADs instead, leaving the LCOS pattern centered.

3.2.5.2 SPAD Array Alignment

The alignment procedure was performed using a concentrated (100 nM) Cy3B dye solution. Cy3B has a wide enough emission band to be detectable by both the donor and acceptor channels. During alignment the laser power was adjusted such as to achieve a high signal-to-noise ratio (SNR) on the SPADs, making the dark counting rates (DCR) of each pixel negligible.

As a preliminary step, the two detectors are manually aligned to the pattern by maximizing the signal of an 8-spot pattern (formed as described in the next section). After the two detectors are roughly aligned, we perform a fine-alignment procedure described below.

Focusing one detector, we perform a software-controlled cross-hair scan of a single-spot LCOS pattern across the approximate centers of each SPAD pixels and record the corresponding intensities traces from the SPADs. The scan pattern (a cross comprised of a horizontal and a vertical segment) provides intensity profiles which are used to identify the X-Y coordinates of each SPAD pixel. The same procedure is repeated for the second detector. From the coordinates of the 8 pixels in each detector, we compute the X-Y offset and the relative rotation of the two detectors. Next, we adjust the SPAD micro-positioners in order to reduce the offset in X-Y and rotation. The scan procedure is iteratively repeated getting new SPAD coordinates, and adjusting the alignment of one SPAD detector until the offset is minimized.

3.2.5.3 LCOS Pattern Formation

Each excitation spot is generated by modulating the incident plane wave with the phase of a spherical (converging) wave. It is easy to derive the phase from geometric considerations (Fig. SI-4).

The relation between phase delay $\Delta\phi$ and physical displacement Δf is:

$$\Delta\phi = \frac{2\pi}{\lambda}\Delta f \quad , \tag{SI.1}$$

where λ is the wavelength. The phase delay to be applied to a plane wave in order to obtain a converging spherical wave is given by:

$$\phi = N\pi - \frac{2\pi}{\lambda}\Delta f \qquad N = 2 \ldots 4 \tag{SI.2}$$

where N is an arbitrary constant, which is only used to ensure that the phase remains inside the LCOS dynamic range. To obtain a convergent spherical wave, Δf should follow the profile of a sphere:

$$\Delta f = \sqrt{r^2 + f^2} - f \quad , \tag{SI.3}$$

or, equivalently:

$$\Delta f = (1 - \cos\theta)\sqrt{r^2 + f^2} \quad . \tag{SI.4}$$

The choice between the two expressions is just a matter of implementation preference.

It is possible to derive a paraxial approximation of the previous formulas which only uses algebraic operations. Using the expansion:

$$g(x) = \sqrt{1 + x^2} \sim 1 + x^2 \quad , \tag{SI.5}$$

The following approximate expression for Δf and ϕ are obtained:

$$\Delta f \sim \frac{r^2}{f} \tag{SI.6}$$

and:

$$\phi \sim \pi\left(N - \frac{2r^2}{\lambda f}\right) \tag{SI.7}$$

The latter expression is the one reported in [11].

To generate an array of spots, we synthesize an array of 8 adjacent “lens patterns” on the LCOS surface. Each “lens pattern” focuses a spot at a focal length from the LCOS (in our case 32 mm). The pitch of the lens pattern on the LCOS is equal to the pitch of the spots at a distance f from the LCOS. The spots are de-magnified by a recollimating lens L3 and the objective lens. Therefore, the pitch of the excitation spots in the sample is controlled by the pitch of the “lens pattern” on the LCOS. The emission pattern on the SPAD plane needs to have the same pitch as the SPAD array, which is 250 µm. This translates into a pitch of 4.1 µm in the sample (60X de-magnification). The overall pattern dimension in the sample is therefore of the order of 30 µm. From the sample to the LCOS, there is a magnification of (250/3 = 83.3), therefore the pitch on the LCOS is nominally 347.2 µm (or 17.4 x LCOS pixels). This value needs to be adjusted experimentally (usually ± 5 %) because of variations of lens focal length from nominal values

and slight differences in the collimation of the incoming laser beam. The experimental pitch can be accurately estimated with the scanning procedure described in Section 3.2.5.2.

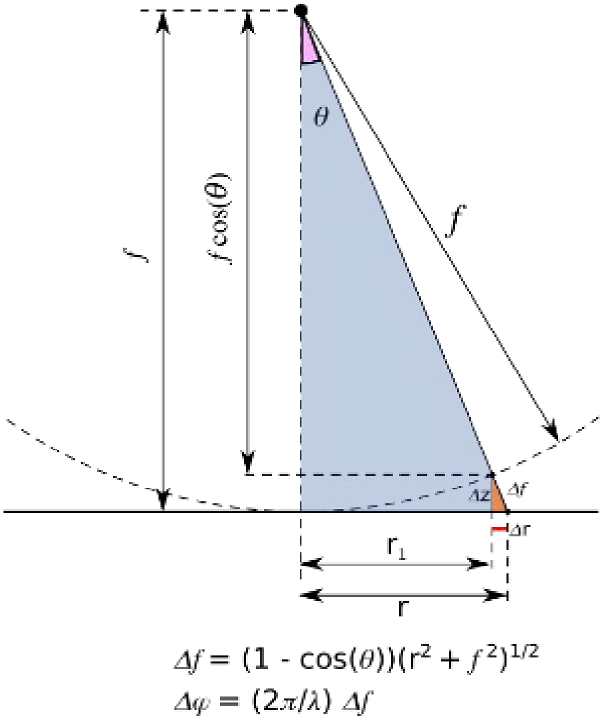

Fig. SI-4: Geometrical construction illustrating how to compute the phase delay of a spherical wave. An incident plane wave propagating top to bottom can be focused at a distance *f* from the reflecting surface (e.g. the LCOS surface) if, after reflection, its wave-front (surface of equal phase) is transformed into a sphere. This happens if the phase is modulated with a radially symmetrical function with suitable dependence on *r*.

Overall the extension of the 8-spot pattern on the LCOS is less than 2.5 mm along the long axis and around 350 µm along the short axis.

Given the pitch and the focal length, the 8-spot LCOS pattern can be generated using equations (SI.1) and (SI.3). In particular, the coordinates of the centers of the *n* spots (in our case, *n* = 8) for a pattern centered around the origin (conventionally chosen as the LCOS center) can be expressed as:

$$\begin{cases} x_i = \left(i - \dfrac{n-1}{2} \right) p \qquad i = 0 \ \ldots \ n-1 \\ y_i = 0 \end{cases} \qquad \text{(SI.8)}$$

In practice, we need to translate this pattern to an arbitrary position *Δ* on the LCOS, and rotate it with respect to the pattern's center. The rotational degree of freedom is needed to match the exact orientation of the detector array (we found that the rotational adjustment was around 1 degree). A rigid transformation (translation + rotation) is a linear operation that can be synthetically expressed as:

$$\widetilde{X_i} = \mathbf{R} X_i + \Delta \qquad \text{(SI.9)}$$

Where $X = [x_i, y_i]$, $\widetilde{X_i}$ are the rotated coordinates and $\mathbf{R}$ is the rotation matrix:

$$\mathbf{R} = \begin{bmatrix} \cos\rho & -\sin\rho \\ \sin\rho & \cos\rho \end{bmatrix} \quad \text{(SI.10)}$$

Finally, for each i, we evaluate function $\Delta\phi$ (Eq. (SI.1)) centered around each spot position $\widetilde{X}_i$ in a region limited to a small square with side equal to the pattern pitch. The LCOS pixels outside this “lens patterns” are filled with a beam steering pattern, which is an alternation of horizontal lines with phase of 0 and π. A zoomed in image of a typical multispot pattern is shown in Fig. SI-5. Readers can find a complete Python implementation for generating multispot LCOS patterns with arbitrary input parameters in the `LCOS_pattern` folder of the notebooks archive (link).

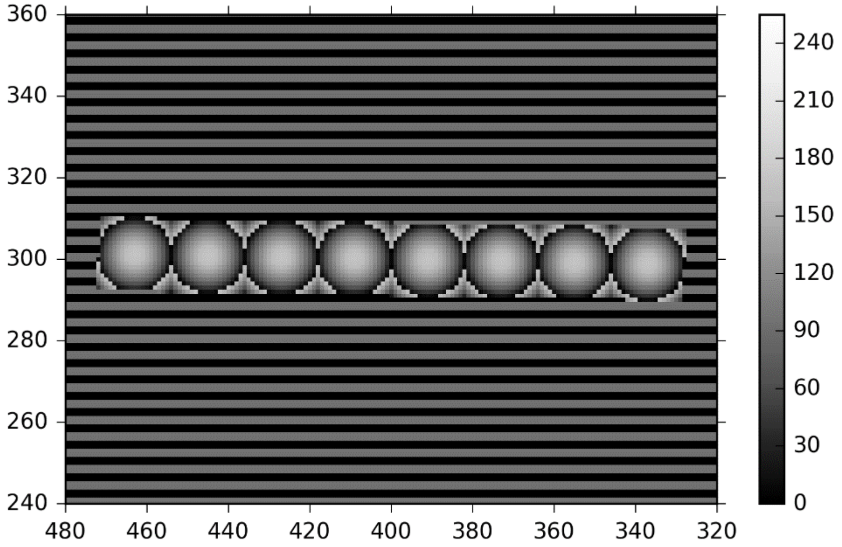

Fig. SI-5: Zoomed in of a typical multispot pattern used to generate 8 excitation spots. The value of each pixel ranges between 0 and 255, matching the grey-scale image format required by the LCOS used in this work. The discontinuity (black to grey transition) inside each spot happens at points where the phase difference from the spot center is larger than 2π. The steering pattern, visible outside the lens pattern, is comprised of alternating stripes of phase 0 and π. Note that the pattern has a rotation angle of 1 degree applied to it.

Appendix 4 Crosstalk & Afterpulsing Analysis

4.1 Crosstalk

The amount of crosstalk between two SPADs measures the correlation between their signals appearing on top the expected correlation due to diffusion or photophysics, in the case of two detectors detecting the signal emitted from the same spot. Unwanted crosstalk between channels can happen due to different reasons:

- electrical signal pick-up
- emission point spread function (PSF) spillover
- intrinsic optical crosstalk

The first kind of crosstalk happens when the electrical pulse coming from one channel is picked up by another line, triggering a pulse detection on another line. It is rare in well-designed setups and electronics.

The second kind of crosstalk can happen if the illumination/detection geometry (in particular the size of the image of each excitation spot) is such that a fraction of the signal emitted from one excitation spot, which should optimally confined to a dedicated SPAD, can be detected by a neighboring SPAD, for instance because the SPAD separation is comparable to the PSF extension. This was not the case in these experiments, where the separation between spots was at least 5 times larger than the PSF extension.

The third type of crosstalk is specific to SPAD arrays and is due to secondary photon emission during each avalanche. Those isotropically emitted photons can reach neighboring SPADs (either through a direct path or after reflection off the chip's surfaces) and can trigger an avalanche in those SPADs [18].

The detailed description of crosstalk analysis of the SPAD arrays used in this study is beyond the scope of this article and will be presented elsewhere. The underlying phenomenon is now well understood and has been described in the literature [18, 19]. Here we merely provide the expression used to compute the crosstalk coefficient l_{AB} between two SPADs A and B, based on the computation of the value of the cross-correlation function of the two signals at time lag 0, $CCF_{AB}(0)$:

$$l_{AB} = \left[\frac{\lambda_A + \lambda_B}{\lambda_A \lambda_B \theta} \frac{1}{CCF_{AB}(0) - 1} - 1 \right]^{-1}, \qquad \text{(SI.11)}$$

Where, θ is the resolution chosen to compute the correlation function and λ_A (resp. λ_B) is the average count rate measured in channel A (resp. B). The only subtlety in this analysis is the choice of the resolution parameter θ, which we briefly address here.

The minimum value is set by the timestamp resolution, imposed by the counting electronics. In these experiments, timestamps where obtained using a 80 MHz clock, defining a resolution of 12.5 ns.

While using this value in Eq. (SI.11) and calculating *CCF(0)* with this resolution provided crosstalk coefficients of the correct order of magnitude, it neglects the fact that quenching the avalanche in a SPAD takes a finite amount of time (typically a few tens of ns), during which the probability of secondary photon emission is not zero. During this time, a crosstalk avalanche can be triggered in a nearby SPAD. It is therefore important to compute Eq. (SI.11) using a resolution θ covering the time scale of the SPAD avalanche. We found that a value of 37.5 ns (3 times the clock resolution) was appropriate for this calculation (using θ = 50 ns provided identical results).

Eq. (SI.11) is equivalent to the expression provided in ref. [19], and can also be expressed in terms of the number of counts detected in each channel during the chosen integration time θ (N_A and N_B), as well as the number of coincident counts C, using:

$$CCF_{AB}(0) = \frac{(N_A + N_B)C}{N_A N_B} \; . \qquad \text{(SI.12)}$$

Tables SI-1 and SI-2 report the measured crosstalk values (in percent) for both SPAD arrays. Because of their extremely small values, crosstalk is therefore not a concern in this study.

SPAD #	1	2	3	4	5	6	7	8
1	0	0.11	0.13	0.12	0.12	0.11	0.09	0.13
2	0.11	0	0.15	0.16	0.16	0.13	0.12	0.13
3	0.13	0.15	0	0.15	0.15	0.17	0.13	0.26
4	0.12	0.16	0.15	0	0.15	0.17	0.17	0.26
5	0.12	0.16	0.15	0.15	0	0.17	0.19	0.28
6	0.11	0.13	0.17	0.17	0.17	0	0.18	0.32
7	0.09	0.12	0.13	0.17	0.19	0.18	0	0.46
8	0.13	0.13	0.26	0.26	0.28	0.32	0.46	0

Table SI-1: Crosstalk coefficients (in percent) for the donor channel SPAD array.

SPAD #	1	2	3	4	5	6	7	8
1	0	0.26	0.27	0.24	0.18	0.17	0.15	0.27
2	0.26	0	0.19	0.27	0.20	0.18	0.16	0.27
3	0.27	0.19	0	0.21	0.25	0.24	0.21	0.26
4	0.24	0.27	0.21	0	0.17	0.22	0.21	0.33
5	0.18	0.20	0.25	0.17	0	0.18	0.25	0.45
6	0.17	0.18	0.24	0.22	0.18	0	0.19	0.41
7	0.15	0.16	0.21	0.21	0.25	0.19	0	0.38
8	0.27	0.27	0.26	0.33	0.45	0.41	0.38	0

Table SI-2: Crosstalk coefficients (in percent) for the acceptor channel SPAD array.

4.2 Afterpulsing

The formula used to compute the afterpulsing probability for each SPAD of both arrays used in this study (Eq. (1) in the main text) is implemented in the ALiX software (linked to in Section 2.2) as a simple script activated by pressing the “Afterpulsing %” button in the Scripting>>Multispot Analysis panel. The only parameter needed for this calculation is the SPAD deadtime, common to all SPADs in these arrays and equal to 160 ns. More information can be found in the corresponding manual page at: https://sites.google.com/a/g.ucla.edu/alix/manual/scripting/multi-spot-correlation-analysis/compute-afterpulsing-probabilities

Table 2 reports, for each SPAD, the mean value and standard deviation computed for the 3 provided data files, using a 1 ms time bin to compute the time trace.

Appendix 5 Photon Streams Definition

In µs-ALEX, photon streams are defined based on a histogram of the “reduced” timestamps of photons in each detection channel (D and A). The reduced timestamp $\bar{t}_i$ of a photon is simply its timestamp value t_i modulo the alternation period T:

$$\bar{t}_i = t_i \bmod (T). \tag{SI.13}$$

As shown in Fig. SI-6, the resulting alternation period histograms exhibits two regions with different count rates, one of which corresponds to the donor excitation period, the other to the acceptor excitation period.

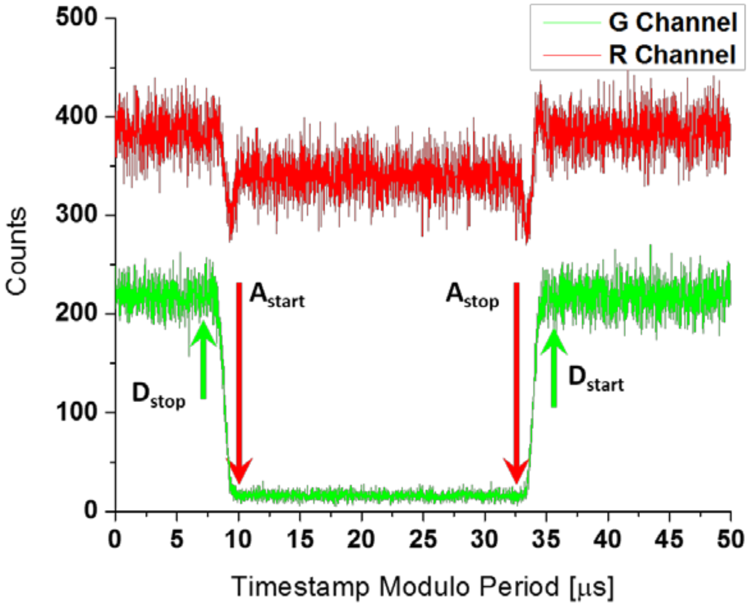

Fig. SI-6: Definition of the donor and acceptor excitation periods within the alternation period in the 12d sample. Histograms of all photons timestamps recorded in the green (G) and red (R) channels are binned with 12.5 ns resolution. The acceptor excitation period is characterized by a lower photon count in the green channel.

The latter is easily identified as the period with almost no donor photon detection (the low detected count rate corresponds to the dark count of the detector and residual background in that channel). Due to the finite response time of the AOM, there are transition regions between donor and acceptor periods, during which both laser lines are partially transmitted. For this reason, two ~2 µs "gap" regions around the donor to acceptor and acceptor to donor transitions are usually rejected. This preprocessing step allows to define the 4 base streams of timestamps: $D_{ex}D_{em}$ (D excitation, D emission channel stream), $D_{ex}A_{em}$ (D excitation, A emission channel stream), A_{ex}-A_{em} (A excitation, A emission channel stream) and $A_{ex}D_{em}$ (A excitation, D emission channel stream). These streams identify photons detected during the D or A excitation periods (D_{ex} or A_{ex}) by the D or A detection channels (D_{em} or A_{em}).

In practice, reduced timestamps might be offset by a few microsecond (t_0) with respect to the alternation period "edges" (the time when one laser is turned off and the other on), as illustrated in Fig. SI-6. In this case, it might be easier to define:

$$\overline{t_i} = \left(t_i - t_0\right)\text{mod}\left(T\right), \qquad \text{(SI.14)}$$

which allows defining each excitation period by a simple condition such as:

$$\begin{aligned} D_{ex} &: \overline{t_i} \in \left[t_{D,start}, t_{D,stop}\right] \\ A_{ex} &: \overline{t_i} \in \left[t_{A,start}, t_{A,stop}\right] \end{aligned} \qquad \text{(SI.15)}$$

Appendix 6 Background Rate Calculation

6.1 Introduction

6.1.1 Sources of background

Sources of background in single-molecule experiments are multiple and commonly include Rayleigh and Raman scattering, scattering or fluorescent impurities, out-of-focus molecules and detector noise. The first two can be practically eliminated by a proper choice of filters. Buffer contributions can consist of single-molecule-like bursts (which will be treated as single-molecule bursts) or uncorrelated background signal, and can be minimized using ultrapure reagents and filtration. The detector dark count rate is easily characterized but can be non-Poissonian. In ideal situations, background rates can be reduced to less than 1 kHz.

6.1.2 Background rate calculation for each measurement

While it is possible to use a buffer sample and use the measured count rates for that sample as background rates for subsequent samples using the same buffer and experimental conditions, this procedure ignores the contribution of out-of-focus molecules present in most samples. Therefore, a procedure extracting the relevant background rates from each sample file is preferable. A simple approach consists in looking at the inter-photon delay distribution, $\varphi(\tau)$, for each stream.

6.1.3 Inter-photon distribution:

For photons generated by a hypothetical background Poisson process, this distribution will be exponential. In the case of single-molecules diffusing through a continuous (non-alternated) excitation spot, this distribution is complex but can be approximated as the weighted sum of two terms, one of which is exponential [20]:

$$\varphi(\tau) \approx (1-p_b)\, g(\tau) + p_b T_b \exp(-\tau/T_b) \qquad \text{(SI.16)}$$

In this expression, $g(\tau)$ is the inter-photon delay distribution for a single molecule diffusing through the excitation volume ($\sim \tau^{-3/2}$ for a 3-dimensional Gaussian PSF, see ref. [21]) and T_b is the mean time between bursts. The first term dominates at short time scales, while the second dominates at long time scales. Since this expression is derived in the absence of external background rate, this means that a background-free single-molecule signal generates an effective Poisson background rate proportional to the concentration, $b = 1/T_b$, to which any additional background source (buffer, detector, etc.)[1] will be added in real experiments [20].

The background rate used for correction is defined as the exponential rate extracted from the long time scale behavior of the inter-photon delay distribution. This rate can be estimated without computing the inter-photon delay histogram (which can be time consuming for large data sets), using the fact that the maximum likelihood estimator (MLE) of the rate parameter of an exponential distribution is simply equal to the inverse of the mean of that distribution:

$$b^{-1} = \frac{1}{n}\sum_{i=1}^{n} \tau_i = \langle \tau_i \rangle \ , \qquad \text{(SI.17)}$$

[1] In this document, we will indifferently refer to detector counts as "photons" or "counts", since there is no way to distinguish between them at the individual count level.

where the τ_i's are the inter-photon delays. Note that the MLE estimator is only one of many possible estimators. Alternatives are the minimum variance unbiased estimator (MVUE) or the estimator which minimized the mean square error. All these estimators only differ by a multiplicative factor depending on *n*. Because the background rate is estimated with a number of inter-photon delays much larger than 1,000, the difference between the various estimators is in general negligible.

As discussed previously, the inter-photon delay distribution is not exponential, only its large time scale behavior is (Eq. (SI.16)). Therefore, only part of the distribution should be used to estimate the background rate. The MLE of the rate parameter *b* of such a truncated exponential distribution ($\tau_i > \tau_{min}$) is simply given by:

$$b^{-1} = \langle \tau_i \rangle_{\tau_i > \tau_{\min}} - \tau_{\min} \quad . \qquad \text{(SI.18)}$$

Choosing τ_{min} involves a trade-off: on one hand, using only the longest time scales of the distribution would result in poor statistics. On the other hand, trying to improve the statistics by extending the range of included time scales towards small values would result in the incorporation of (short) inter-photon delays from within single-molecule bursts, biasing the background rate estimates (towards larger values). Optimization of the lower inter-photon delay used for background rate estimation can be performed systematically, but its study is beyond the scope of this paper. For the experiments in this paper, we computed the τ_{min} threshold with a previously described, simple iterative algorithm [22]. While it is possible (for example using the FRETBursts software [22]) to perform a global optimization in order to find the optimal τ_{min} for background

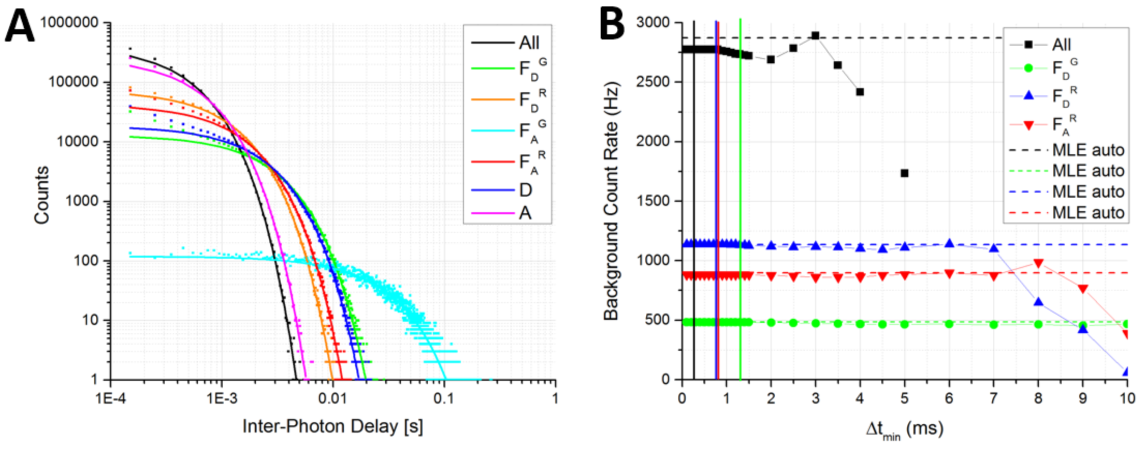

estimation, this was not necessary as the procedure only yielded negligible differences.

Fig. SI-7: Robust Fit of Background Count Rates. (**A**) Inter-photon delay histograms (bin = 100 µs) for different photons sets of file 006_dsDNA_7d_green100u_red40u.hdf5 (points) and their robust fit with a single exponential (curves). D (resp. A or All) indicates donor (resp. acceptor or all) photons, irrespective of their time of emission and detection channel. F_D^G = D_{em}, F_D^R = A_{em}, F_A^R = $A_{ex}A_{em}$. Fits were performed with an automatically computed Δt_{min} for each set (Δt_{min} = 1/<count rate>, where <count rate> is the mean count rate for that set, computed over the whole experiment). (**B**) Dependence of the background count rate estimated by robust fit of the tail of the inter-photon delay histogram on the minimum interval. The points correspond to the fitted rate obtained using the specified minimum inter-photon delay Δt_{min} for 4 representative sets of photons. Fits were performed using a single exponential model, statistical weights and the bisquare algorithm implemented in LabVIEW 2015 with default parameters. The vertical lines indicate the location of the automatically determined Δt_{min} for each set of photons. The dashed horizontal lines correspond to the MLE result for each photon set, using the automatically determined Δt_{min} for each set. Except for the "all photons" set, the agreement between MLE and fitted background rates is excellent. Notice how the fitted

count rate deviates from the automatically determined value only for very large Δt_{min} and is otherwise practically constant in the range 0 – 1 ms for the “all photons” set and well past 5 ms for the other sets.

An alternative approach (resulting in similar results) consists in performing a robust fit (e.g. a non-linear least square fit using Tukey’s biweight [23], also known as a bisquare fit) of the inter-photon delay histogram of each stream, for inter-photon delay larger than a minimum inter-photon delay λ_{min}. The definition of λ_{min} is such that the resulting background count rate is, to a good approximation, not dependent on it. An example of definition of λ_{min} is the average count rate in the photon stream (as illustrated in Fig. SI-7), but the advantage of this robust fit approach is that the result does not depend on the value chosen for λ_{min} over a large range of values. Its disadvantage is that it requires calculating inter-photon delay histograms, which can be memory and CPU intensive for large files (the fit itself is fast).

6.2 Variable background rates.

If the background rates vary during the experiment (for instance due to focus drift, evaporation or other reasons), background estimation needs to be performed piecewise over time windows during which rates can be considered as approximately constant, but long enough to ensure proper estimation of this “local” background rate. In this work, a few files exhibited varying background rates justifying using this approach (Fig. SI-8A & B). Note that for the single-spot μs-ALEX measurements, the background levels were low enough that even a significant variation did not result in major differences between results obtained with or without time-dependent background rate calculation (Fig. SI-8C-F). Because background levels were larger in multispot measurements, and for consistency, time-dependent background rates (typical window size: 30 s) were used.

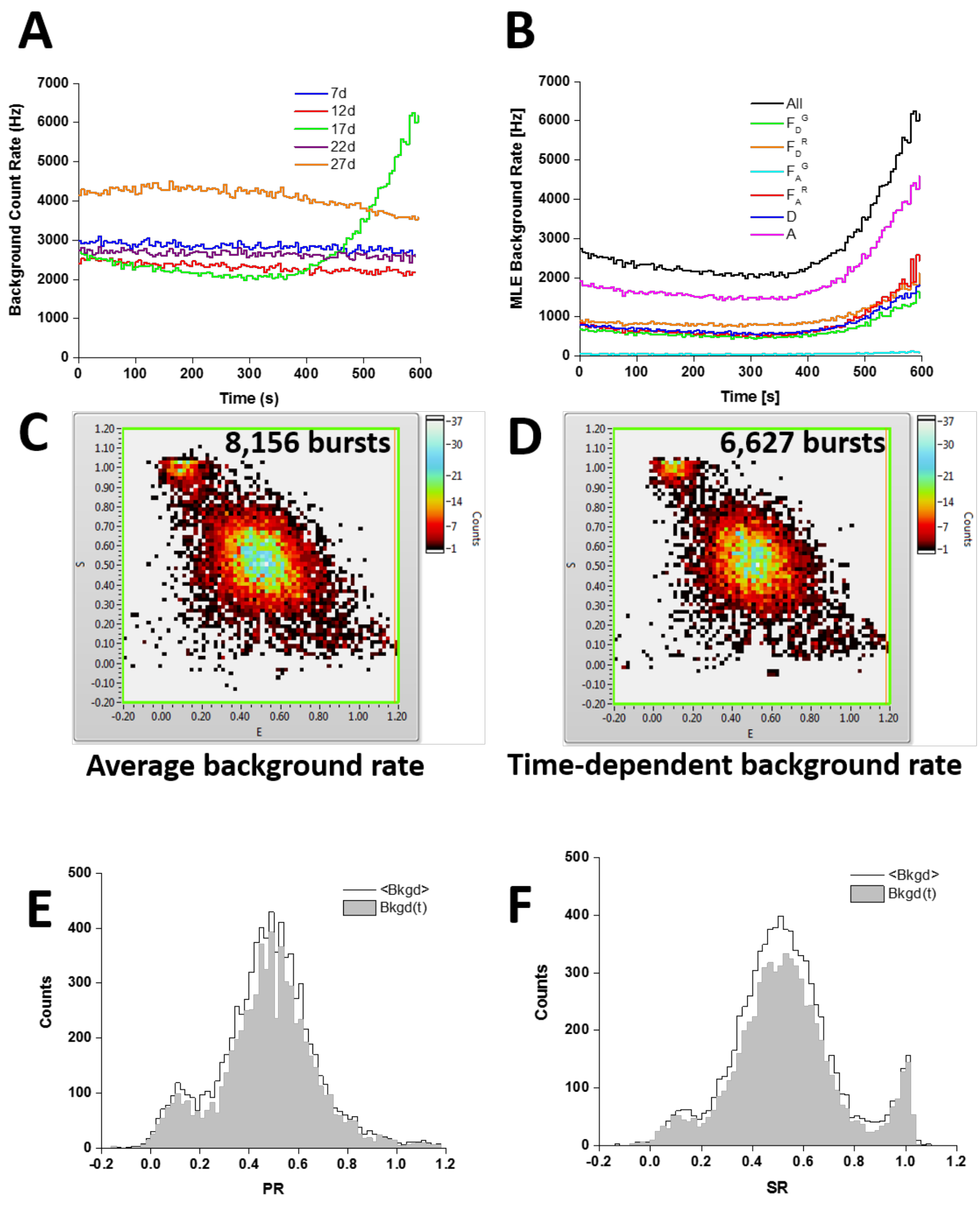

Fig. SI-8: Time-Dependent Background Rate Fit. (**A**) Time-dependent maximum likelihood estimates (MLE) of the total background rates for all single-spot µs-ALEX measurements. The MLE calculation was performed on consecutive windows of 5 s duration, using the automatically determined Δt_{min} for each set (Δt_{min} = 1/<count rate>). The large variation for sample 17d is further studied in the other panels. (**B**) Time-dependent MLE of the background rates of different photons streams of the 17d sample. Notice how the background rate first decreases, then increases significantly during the last third of the measurement, most likely due to evaporation. (**C**) ALEX histogram (background corrections only) of the 8,156 burst of total size S > 30 obtained when using constant mean background rates for each photon set throughout the whole acquisition. (**D**) ALEX histogram (background corrections only) of the 6,627 burst of total size S > 30 obtained when using time-dependent background rates for each photon set (as computed in A). (**E**) Proximity ratio histograms of all bursts in C (black) and D (grey). Notice that, while the number of detected bursts is smaller when using a variable background rate, the characteristics of each sub-population are barely affected by the difference in background rates. (**F**) Stoichiometry ratio histograms of all bursts in C (black) and D (grey). As for the proximity ratio histogram, notice that the characteristics of each sub-population are barely affected by the difference in background rates.

6.3 Special consideration for μs-ALEX

A final comment is in order regarding μs-ALEX background analysis. Due to laser alternation, photon streams are periodically interrupted. Therefore, the photon and background rates within a stream periodically drop to almost zero (in practice, down to a value close to the detector dark count rate). The background rates fitted as described, are therefore rates averaged over the whole alternation period.

The "instantaneous" background rate alternates between a high value (hb_X^Y, $h > 1$, when excitation laser X is on) and a low value (lb_X^Y, $l < 1$, when excitation laser X is off), where b_X^Y is the background rate in channel Y during excitation period X (X and Y are either D or A). The average background rate is a weighted sum of these two rates:

$$b_X^Y = \frac{1}{T}\left(T^+ \times hb_X^Y + T^- \times lb_X^Y\right), \quad \text{(SI.19)}$$

where T^+ (resp. T^-) is the duration of the X laser-on (resp. -off) period, and $T^+ + T^- = T$, the alternation period (generally of the order of a few tens of μs)[2].

Correspondingly, we can divide a burst duration into alternate X laser-on segments, $\{\delta T_i^+\}_{i=1,\dots,p}$ and X laser-off segments, $\{\delta T_i^-\}_{i=1,\dots,m}$. The expected total background counts during the burst duration are thus:

$$\begin{aligned} B_X^Y &= hb_X^Y \sum_{i=1}^{p} \delta T_i^+ + lb_X^Y \sum_{i=1}^{m} \delta T_i^- \\ &= hb_X^Y \times \Delta T^+ + lb_X^Y \times \Delta T^- \end{aligned} \quad \text{(SI.20)}$$

where ΔT^+ (resp. ΔT^-) is the total duration of X laser-on (resp. -off) periods during the burst.

We can therefore rewrite the background correction term used in this work as a function of the expected background counts as:

$$\begin{aligned} b_X^Y \times \Delta T &= \frac{\Delta T}{T} T^+ \times hb_X^Y + \frac{\Delta T}{T} T^- \times lb_X^Y \\ &= B_X^Y + \left(\frac{\Delta T}{T} T^+ - \Delta T^+\right) \times hb_X^Y + \left(\frac{\Delta T}{T} T^- - \Delta T^-\right) \times lb_X^Y \end{aligned} \quad \text{(SI.21)}$$

The last two terms are equal to zero when the burst duration ΔT is a multiple of the laser alternation period T: $\Delta T = nT$ (in other words, the background correction term is equal to the true estimation), since:

$$\begin{aligned} \Delta T &= nT \\ &= \Delta T^+ + \Delta T^- \\ &= nT^+ + nT^- \end{aligned} \quad \text{(SI.22)}$$

In the special case where the burst duration contains exactly one more complete on-period than off-period ($p = m + 1$):

[2] Note that Eq. (SI.19) uses the fact that the "gap" regions discussed in Appendix 5 have a negligible influence.

$$\begin{aligned} \Delta T^{+} &= \sum_{i=1}^{p} \delta T_i^{+} = pT^{+} \\ \Delta T^{-} &= \sum_{i=1}^{m} \delta T_i^{-} = mT^{-} \\ \Delta T &= \Delta T^{+} + \Delta T^{-} = mT + T^{+} \end{aligned} \tag{SI.23}$$

We can thus rewrite:

$$\begin{aligned} b_X^Y \times \Delta T &= B_X^Y + \left(\left(m + \frac{T^+}{T} \right) T^+ - pT^+ \right) \times hb_X^Y + \left(\left(m + \frac{T^+}{T} \right) T^- - mT^- \right) \times lb_X^Y \\ &= B_X^Y - \frac{T^- T^+}{T} \times \left(hb_X^Y - lb_X^Y \right) \sim B_X^Y - \frac{T^+}{2} \times \left(hb_X^Y - lb_X^Y \right) \\ &\sim B_X^Y \left(1 - \frac{1}{2p} \right) \end{aligned} \tag{SI.24}$$

where the last two approximations use the fact that the on and off durations are approximately equal to one half the total alternation period *T*. The difference between the background correction term used in this work (the left hand side term) and the "true" background contribution, B_X^Y, is thus equal to a fraction of the correction term, and is increasingly negligible for longer burst duration. Bursts with duration comparable to the alternation period contain in general very few counts and are not normally selected for further analysis, justifying using the background correction term $b_X^Y \times \Delta T$ in all cases.

Appendix 7 Details on Burst Search

7.1 Photon Streams

The photon stream is simply the set (or sets) of photons used for the search. The simplest choice consists in using all photons ("all photons burst search", APBS) [24] but others can be defined to focus the analysis on specific species (footnote [3]).

While different sets of photon streams can in principle be chosen to identify different types of molecules during burst search, it is often best to perform a generic single-molecule burst search (*e.g.* "all photons burst search", APBS) and perform the burst selection at a later stage, as

---

[3] Other possibilities are, for instance, to use only donor channel photons ("donor emission burst search", or D_{em}BS: all photons recorded by the donor channel, irrespective of the laser alternation period they were emitted in), or acceptor channel photons ("acceptor emission burst search", or A_{em}BS: same as above, but for acceptor photons), donor excitation period photons ("donor excitation burst search", or D_{ex}BS: all photons recorded by either channels but limited to the donor laser on period), or acceptor excitation period photons ("acceptor excitation burst search", or A_{ex}BS: same as above, but for the acceptor laser). Any of these burst searches can be combined using logic AND or OR operations. The AND combination of two searches simply keeps the burst parts overlapping in both searches (in other words, their intersection, ∩). The OR combination of two searches returns all bursts found in either search, fusing any two overlapping bursts into a single, larger burst (their union, ∪). For instance, the "dual channel burst search" (DCBS) defined in ref. 21 corresponds to the intersection (OR operation) of a donor excitation and an acceptor excitation burst searches: DCBS = D_{ex}BS AND A_{ex}BS.

discussed in the next subsection. Most analyses presented in this work used APBS (unless specified otherwise), after we verified that the final results were not affected by this choice.

7.2 Burst Threshold

7.2.1 Standard Definition of the Burst Threshold

Bursts are defined by their start and end counts, identified respectively as counts in the stream where the observed local count rate, $r_m(t_i)$, computed over m consecutive counts starting with time stamp t_i:

$$r_m(t_i) = \frac{m-1-c}{t_{i+m-1} - t_i} \qquad \text{(SI.25)}$$

changes from "likely to be due to background only" to "unlikely to be due to background only", and vice versa [16, 25, 26]. The role of parameter c in the previous equation, which was historically set to 0, is discussed in Section 7.2.2.

Burst searches using a minimum SBR criterion compare the value of Eq. (SI.25) to a threshold value defined as F times the local background count rate $b(t_i)$ [25]:

$$r_m(t_i) \geq F.b(t_i). \qquad \text{(SI.26)}$$

If the background count rate changes over time, the threshold also changes, guarantying a minimum SBR $\geq F - 1$.

While such a criterion is appropriate for single-spot µs-ALEX experiments of samples characterized by similar background rates, molecular brightness and excitation intensity, it might not always be ideal. For instance, using a minimum SBR criterion might affect the comparison of multiple experiments, if the background rate varies significantly from experiment to experiment. In particular, the large dark count rates of some of the detectors used in our multispot experiments, resulted in very different background rates among spots and detection channels. In these cases, a fixed minimum count rate criterion is preferable, as discussed in Section 4.2.3 of the main text and in Section 7.4 below.

7.2.2 Other Definitions of the Count Rate

The family of count rate estimators defined in Eq. (SI.25) has different properties for different values of c. For example, the MLE estimator is obtained for $c = 0$, the minimum variance *unbiased* estimator is obtained for $c = 1$, the estimator minimizing the mean square error is obtained with $c = 2$ while $c = 1/3$ yields an estimator of the median count rate (see this notebook for details).

To verify that the case $c = 1$ yields an unbiased estimator of the rate for a Poisson process, consider m consecutive photons generated by a Poisson process with rate λ. Let t_i, t_{i+1}, …, t_{i+m-1} be the timestamps of the m photons, and:

$$\Delta t_i^{(j)} = t_{i+j-1} - t_i \ , \qquad \text{(SI.27)}$$

the delay between the last and first photon in a bunch of j successive photons. It is well known that the probability distributions of these quantities are Erlang distributions:

$$p\left(\Delta t_i^{(j)}\right) = \frac{\lambda^{j-1}}{(j-1)!}\left[\Delta t_i^{(j)}\right]^{j-2} \exp\left(-\lambda \Delta t_i^{(j)}\right) . \qquad \text{(SI.28)}$$

Using this formula, the mean value of the inverse of t_{i+m-1} - t_i (from which the count rate is calculated) is:

$$\left\langle \frac{1}{\Delta t_i^{(m)}} \right\rangle = \int_0^{\infty} du \frac{1}{u} \frac{\lambda^{m-1}}{(m-1)!} u^{m-2} \exp(-\lambda u) = \frac{\lambda}{m-2} \quad . \qquad \text{(SI.29)}$$

Consequently, to define a count rate from m consecutive timestamps in a manner resulting in an average count rate equal to the actual count rate λ (unbiased estimator), the following definition is needed (c = 1):

$$r_i = \frac{m-2}{t_{i+m-1} - t_i} \quad . \qquad \text{(SI.30)}$$

The exact version of the rate estimator (i.e. the value of c) used for burst search or burst statistics analysis is not critical, as long as the analysis is carried out consistently, using the same definition. Changing (*e.g.* increasing) c, while leaving all other burst search parameters unchanged, will change (*e.g.* decrease) the effective threshold used during bursts search. Note also that Eq. (SI.26) can be equivalently rewritten as a condition on $t_{i+m-1} - t_i$ (the difference between the last and first timestamps in the current set of m counts):

$$t_{i+m-1} - t_i < \frac{m-1-c}{r_m(t)} = T \, , \qquad \text{(SI.31)}$$

where T in the previous equation corresponds to the T parameter in the original publication introducing burst search [25], with the definition:

$$T = \frac{m-1-c}{F \; b(t)} \, . \qquad \text{(SI.32)}$$

7.3 Burst Fusion

A typical burst search performed as described above, yields distributions of burst sizes, burst durations and burst separations which are generally monotonically decreasing. Burst separations, in particular, exhibit two general characteristic time scales: a short time scale corresponding to individual molecule exit from and reentry in the excitation spot, and a longer time scale corresponding to the typical interval between different molecules entering the excitation spot. The latter time scale decreases as concentration increases, while the former scales inversely to the molecule's diffusion coefficient. Some analyses may benefit from considering bursts separated by short time intervals as independent (see for instance ref. [27]).

In some other cases, it might be preferable to fuse consecutive bursts separated by less than some minimum time, in order to increase the photon statistics of the resulting burst. The counts detected between the two fused bursts are mostly comprised of background, but are taken care of by background subtraction. It is worth mentioning that, even with background correction, the noise associated with these mostly background gap periods is added to the fused burst signal.

All results discussed in this paper were obtained without any burst fusion.

7.4 Burst Search Influence on Burst Statistics

The impact on single-molecule analysis of the burst search criteria is a common problem in single-spot measurements. In the case of multispot experiments, an additional complexity arises from the actual differences between each single-spot experiment performed in parallel. Some of these differences are discussed in the main text (Section 4.3). Here, we examine their influence

on burst search results by focusing on the variations of a few observables as a function of burst search parameters. The effect of burst selection criteria on these observable is of course important as well, and needs to be considered carefully. The example of the influence of the minimum burst size is discussed in Appendix 8. Details on the definition of the observables discussed next can be found in Appendix 14.

By definition of a burst, the burst count rate (as calculated with Eq. (SI.30)) is initially low, increases and eventually decreases at the end of the burst (with possible fluctuations in between). Therefore, considering a particular burst, increasing the count rate threshold defining the beginning and end of the burst, will reduce its duration (the burst will start later and end earlier, everything else being equal) as well as reduce its background-corrected size. However, this should not affect the peak detected count rate during the burst, since the peak is typically attained well within the burst and not close to its beginning or end.

Such an increase of the effective count rate threshold is exactly what happens when a fixed set of search parameters (m, F) is used and a larger background rate is encountered in one spot compared to another. In other words, using the same SBR criterion for all spots will result in reduced burst duration and size for spots characterized by a larger background rate (a significant part of which is due to detector dark counts in our case). The peak burst count rate of identical species, however, should not be affected, provided all other acquisition parameters are identical (excitation intensity, alignment and molecule brightness).

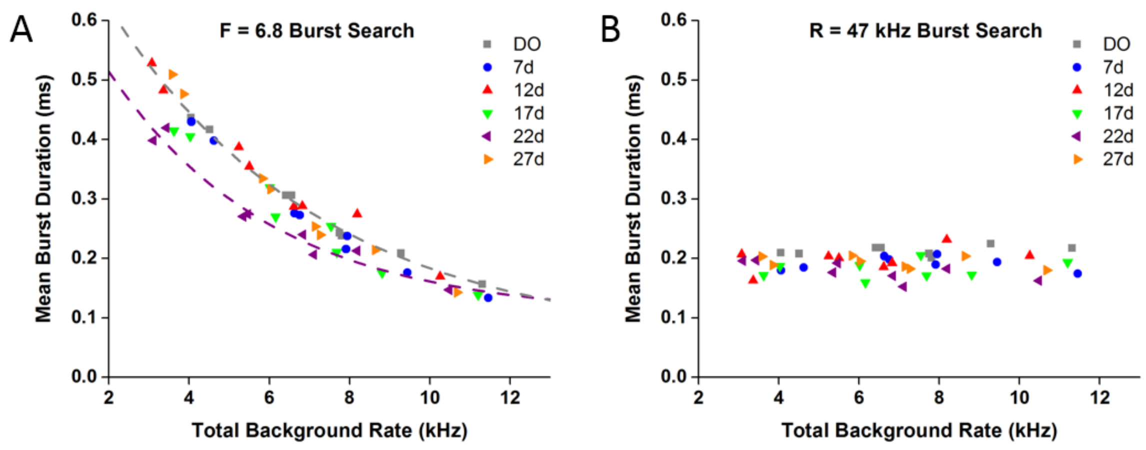

Fig. SI-9: Mean burst duration dependence on total background rate. The mean duration of bursts with brightness-corrected size larger than 30 was computed for all samples (each sample represented with a different color) and each measurement spot within the samples, using two types of burst search. **(A)** $m = 5$, $F = 6.8$. **(B)** $m = 5$, fixed burst threshold rate $R = 47$ kHz. When a constant SBR search is performed (A), the mean burst duration is smaller when the background rate is larger, but does not depend on the sample or spot within a sample, provided the experimental conditions are similar (excitation intensity, alignment, molecular brightness, etc.). Note that the 22d sample trend (purple dashed curve) departs from the trend of all other samples (grey dashed curve), consistent with the observed shorter diffusion time measured for this sample.

As shown in Fig. SI-9A, the first conclusion is supported by our measurements. The average burst duration (irrespective of the sample or spot considered) falls on a universal decreasing curve when a constant minimum SBR criterion is used to detect bursts ($m = 5$, $F = 6.8$). Note that sample 22d, for which FCS analysis detected a shorter diffusion time, is characterized by noticeably smaller average burst durations, confirming the existence of a difference in the experimental

conditions for this experiment. By contrast, using a fixed minimum count rate threshold to detect bursts (Fig. SI-9B, r_{min} = 47 kHz), results in a mean burst duration which is independent of the sample or spot studied, as expected. The mean burst duration is also smaller, because the minimum count rate chosen (r_{min} = 47 kHz) is close to the largest minimum count rate used across all samples and all spots in Fig. SI-9A. The same conclusion is illustrated on Fig. SI-10, where the complete distributions of burst duration and separation for all spots and all samples are represented. These distributions superimpose only when a r_{min} = 47 kHz search is performed, while they are offset with respect to one another if a constant SBR search is used.

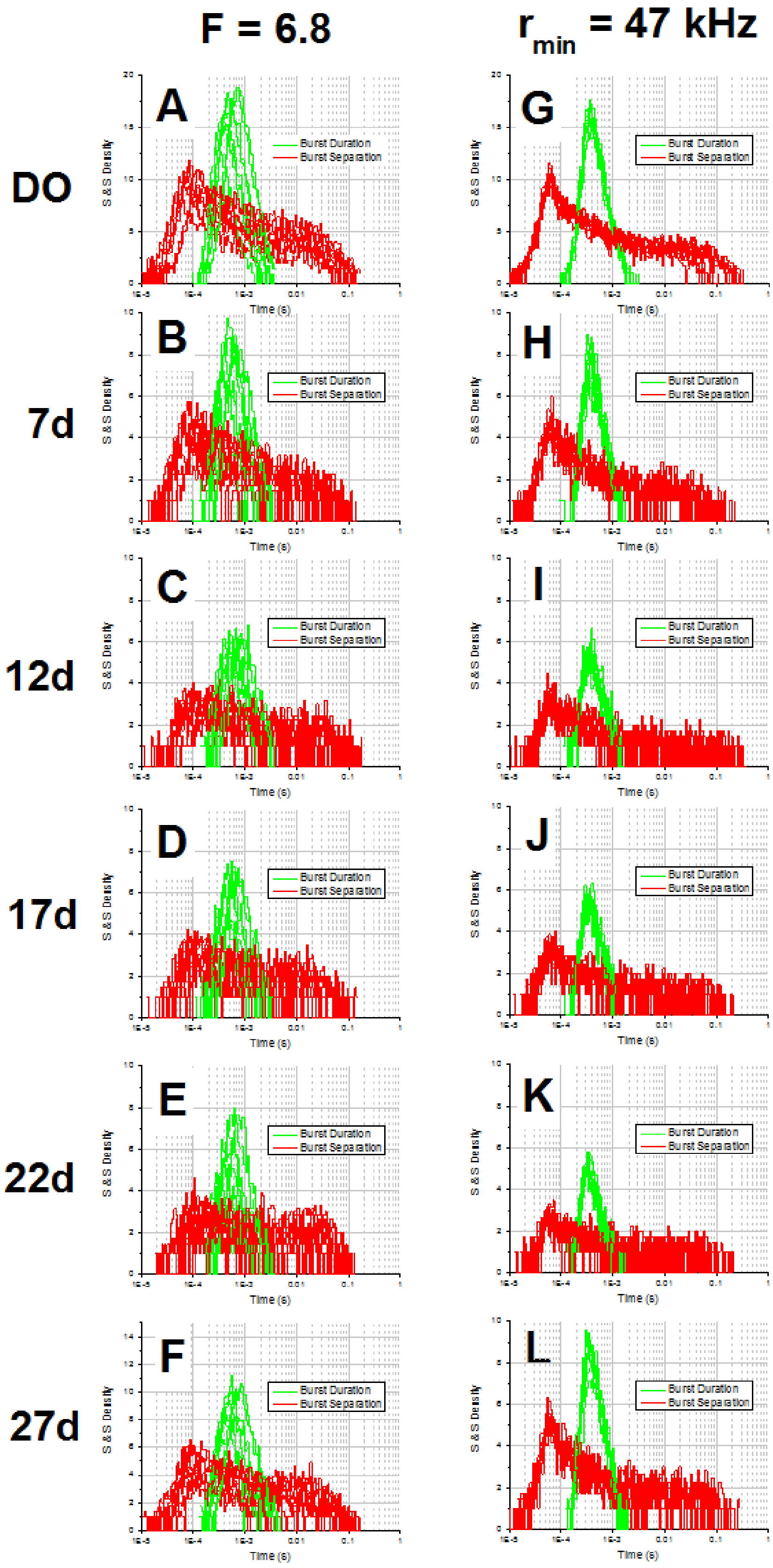

Fig. SI-10: Burst Duration and Sepa-ration Distributions. Two types of burst search were performed on the 6 samples studied in this work (donor only: DO, doubly-labeled: 7d to 27d): (**A-F**) a constant (m, F) = (5, 6.8) search, adjusting the burst count rate threshold to the measured sample/spot background rate (left column), and (**G-L**) a constant burst count rate threshold (r_{min} = 47 kHz) corresponding to 6.8 times the largest Donor + Acceptor channel dark count rate among all spots. After selection of all bursts with a minimum brightness-corrected size $\tilde{F}_{SBC}$ > 30, the burst duration (green plots) and separation (red plots) distribution for each spot were represented using a log-linear representation introduced by Sigworth & Sine [28]. For the spot-dependent search (left column), both distributions clearly depend on the spot studied, while in the other case (constant threshold), the burst duration PDFs of all spots are essentially identical and very similar from sample to sample. A similar observation holds for the burst separation PDFs, with the exception of the long time scale tail, which is underpopulated for spots with large background rates, due to the culling of bursts with insufficient sizes.

The predicted dependence of burst size on background rate, using a minimum SBR criterion (m = 5, F = 6.8), is also clearly visible in Fig. SI-11A. The larger the background rate, the smaller the mean burst size computed over all detected bursts (filled symbols). Indeed, a larger background rate results in the loss of smaller bursts during the search. This dependence is much more pronounced if small bursts are eliminated by imposing a minimum brightness-corrected burst size selection criterion ($F_{SBC} \geq 30$, open symbols).

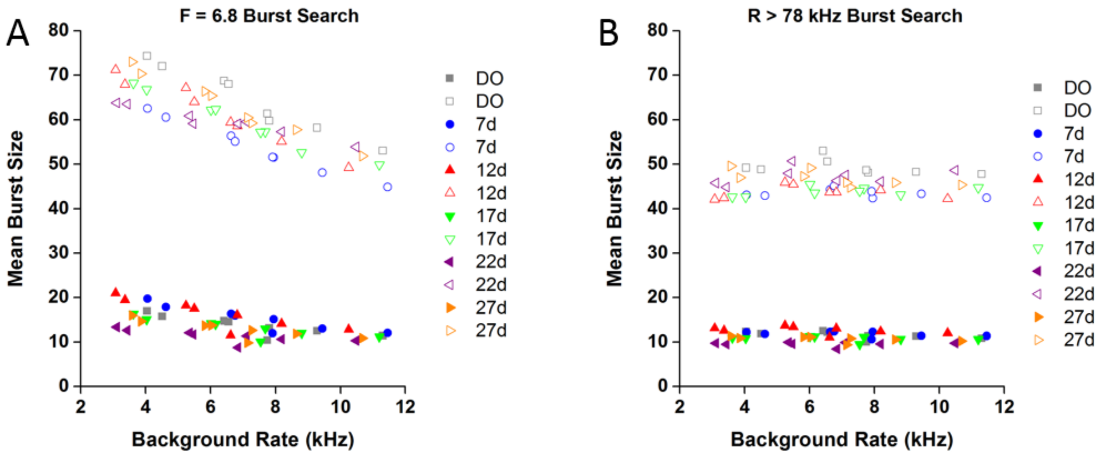

Fig. SI-11: Mean brightness-corrected burst size dependence on total background rate. The mean brightness-corrected size (F_{SBC}) of bursts was computed for all samples (each sample represented with a different color) and each measurement spot within the samples, using two types of burst search. **(A)** m = 5, F = 6.8. **(B)** m = 5, fixed burst threshold rate r_{min} = 78 kHz. Two values are reported for each spot and each sample: the mean F_{SBC} of all burst (plain symbols) and the mean F_{SBC} of bursts with $F_{SBC} \geq 30$ (open symbols). The latter is larger because small size bursts are not included. When a constant SBR search is performed (A), the mean burst size decreases when the background rate increases, but does not depend on the sample or spot within a sample, provided the experimental conditions are similar (excitation intensity, alignment, molecular brightness, etc). If a common minimum count rate is used for burst search (B), the mean F_{SBC} does not depend on background rate, spot or sample.

By contrast, using a fixed minimum count rate threshold (Fig. SI-11B, r_{min} = 78 kHz), results in a mean burst size which is independent of the sample or spot considered. By limiting the analysis to bursts whose brightness-corrected size is larger than a minimum value (set here to 30), the mean burst size naturally increases (open symbols), but remains independent of which sample or spot is considered.

In conclusion, the type of burst search used during analysis can have a significant influence on some burst statistics. This influence needs to be thoroughly characterized when comparing data from difference measurements, or, as in the case of this study, data obtained from different spots during the same experiment and with the same sample.

Appendix 8 PR and SR Histograms

8.1 Standard Histograms[4]

Background correction of raw burst counts F_X^Y (counts collected during X-excitation in the Y-channel) is obtained by subtracting $b_X^Y \times \Delta T$, where b_X^Y is the photon stream's background rate computed as described in Appendix 6 and $\Delta T = t_e - t_s$ is the burst duration, where t_s is the burst start photon timestamp and t_e, the burst end photon timestamp:

$$\overline{F}_X^Y = F_X^Y - b_X^Y \times \Delta T \,. \qquad \text{(SI.33)}$$

Note that Eq. (SI.33) uses the total duration of the burst and the average background rate for the selected photon stream. As discussed in Appendix 6.3, this formula is appropriate only for bursts long enough compared to the alternation period, which is the case in our analysis.

In the following, all overlined quantities are background-corrected quantities.

A useful quantity is the total background-corrected burst size:

$$\overline{F} = \sum_{\substack{X=D,A \\ Y=R,G}} \overline{F}_X^Y = \overline{F}_D^G + \overline{F}_D^R + \overline{F}_A^R \,. \qquad \text{(SI.34)}$$

An alternative quantity, convenient in the absence of acceptor excitation laser, is the donor excitation background-corrected burst size:

$$\overline{F}_D = \sum_{\substack{X=D \\ Y=R,G}} \overline{F}_X^Y = \overline{F}_D^G + \overline{F}_D^R \qquad \text{(SI.35)}$$

From the background-corrected burst observables $\overline{F}_X^Y$, two important quantities can be computed:

- the *proximity ratio, PR,* defined as:

$$PR = \frac{\overline{F}_D^R}{\overline{F}_D^R + \overline{F}_D^G} = \frac{\overline{F}_D^R}{\overline{F}_D} \quad . \qquad \text{(SI.36)}$$

- the *stoichiometry ratio, SR,* defined as:

$$SR = \frac{\overline{F}_D^G + \overline{F}_D^R}{\overline{F}_D^G + \overline{F}_D^R + \overline{F}_A^R} = \frac{\overline{F}_D}{\overline{F}_D + \overline{F}_A^R} \quad . \qquad \text{(SI.37)}$$

The stoichiometry ratio requires two laser excitations, and allows distinguishing between D-only labeled molecules (characterized by *SR* ~ 1), A-only labeled molecules (characterized by SR ~ 0) and doubly-labeled molecules (characterized by *SR* ~ 0.5).

PR and *SR* histograms of these quantities, or the joint (*PR*, *SR*) 2-dimensional histogram, also referred to as the ALEX histogram, are used during different steps of the analysis. Traditionally, they are computed using a bin size of 0.02-0.04, and cover a range slightly larger than the [0, 1] interval in which ratios of quantities not corrected for background would normally fall. In this

[4] Starting with this appendix, notations in this Supporting Material depart from the simpler notations introduced the main text in order to better distinguish several related quantities.

work, some of the joint (*PR*, *SR*) distributions are represented using hexagonal bins, as produced by the FRETBursts software [22, 29].

8.2 Weighted PR Histogram

Rejection of small bursts right after burst search is a standard analysis step. The choice of the minimum burst size S_{min} to include in any further analysis is important and deserves some care. Increasing S_{min} obviously reduces the number of selected bursts. This decrease is exponential (Fig. SI-12), with a characteristic size depending on the burst search type and parameters. This decrease in the final number of bursts, detrimental for sampling statistics, is however compensated by a reduction of variance of most observables, such as the PR histogram's width (Fig. SI-13). This is due to the reduction of shot noise broadening, as more small bursts are rejected. This effect can help separating two nearby *PR* peaks, as illustrated in Fig. SI-13, where the presence of two distinct peaks in the *PR* histograms of sample 17d, measured in spot 2 of the multispot setup, requires $S_{min} \geq 30$ to be clearly distinguishable.

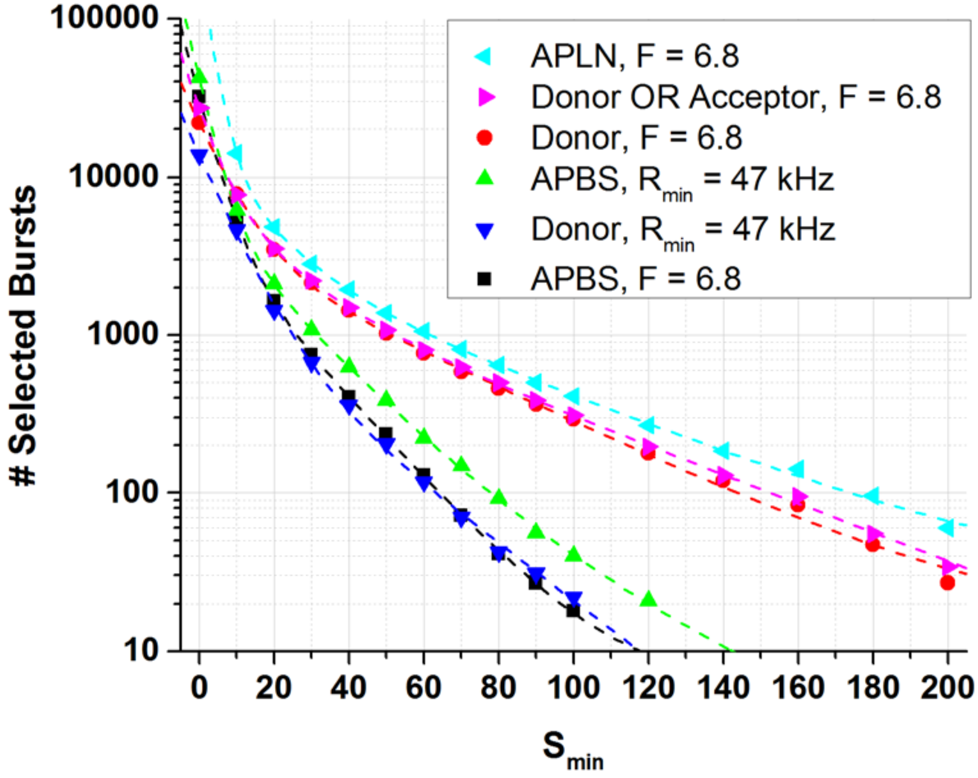

Fig. SI-12: Dependence of burst number on the minimum burst size S_{min}. Dependence of the total number of selected bursts on the minimum burst size selection parameter S_{min}, illustrated with sample 17d, spot 2. Results for different types of burst searches are shown. Notice in particular how the standard all photon burst search (APBS) leads to the smallest number of bursts, due to the fact that the large background rate in the donor channel, used for both donor-only or FRET bursts detection, results in a higher loss of FRET bursts. APLN: all-photon burst search, using the lowest of the two donor and acceptor channel background rates; Donor (resp. Acceptor): search limited to the donor (resp. acceptor) channel photons; Donor OR Acceptor: search corresponding to the union of a Donor and an Acceptor search; $F = 6.8$: search using a count rate threshold computed as F times the background rate; $R_{min} = 47$ kHz: fixed count rate threshold search. $m = 5$ was used for all searches. The dashed curves are multi-exponential fits to the data.

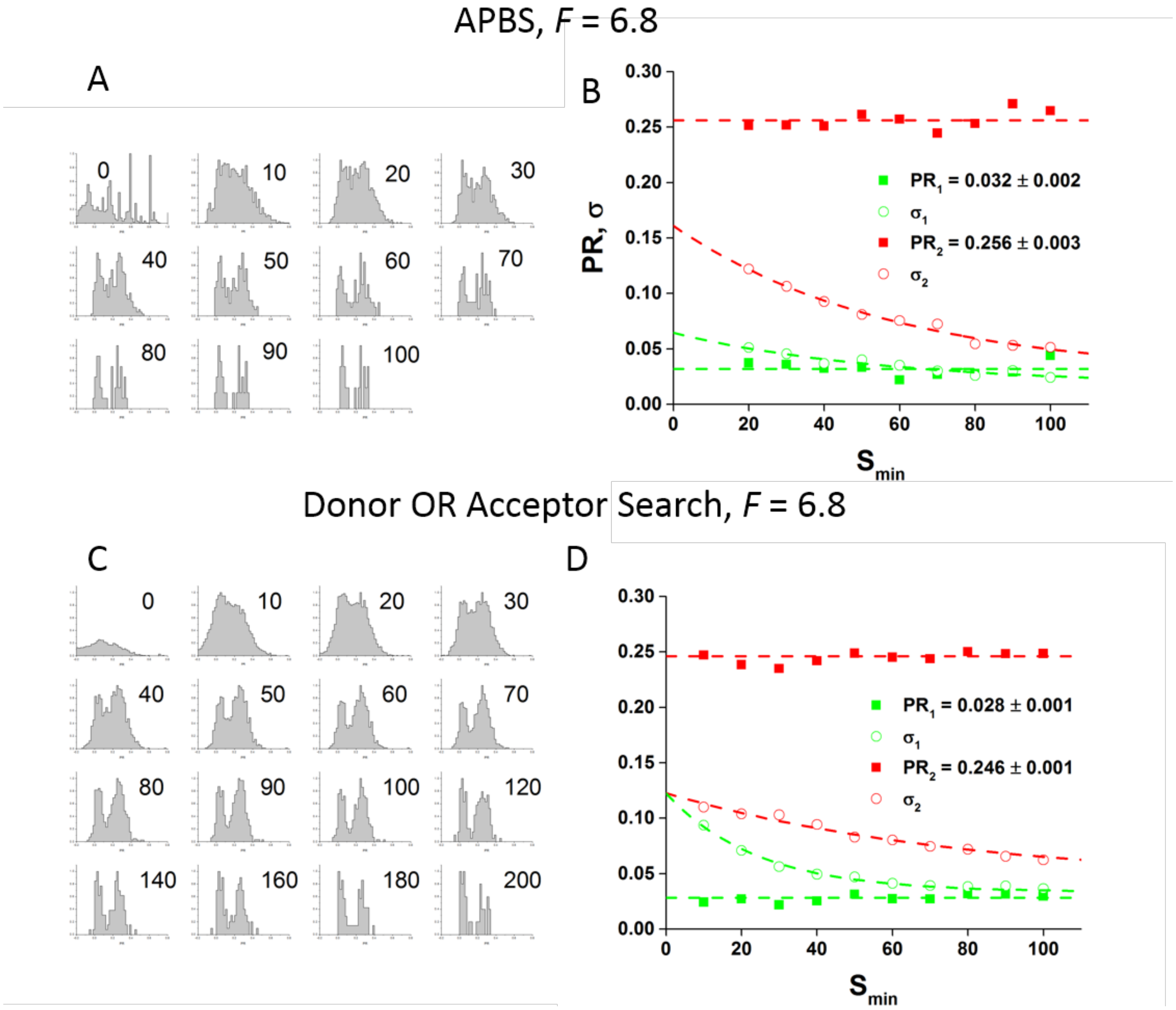

Fig. SI-13: Dependence of proximity ratio statistics on the minimum burst size *S_{min}*. Normalized PR histograms of bursts found by an $m = 5$, $F = 6.8$ all photons burst search (APBS) (**A**), or a donor OR acceptor burst search (OR gate of pure donor-channel search and a pure acceptor-channel search) (**C**). S_{min} is indicated for each plot. The horizontal axis covers [0, 1]. Both searches were followed by selecting bursts with a minimum total burst size $\overline{F}$ after background correction, $\overline{F} = \overline{F}_D^G + \overline{F}_D^R \geq S_{\min}$. Most PR histograms (shown on the left) exhibit two peaks which can clearly be identified for $S_{min} \geq 30$ (donor-only and FRET population). Fitting the *PR* histogram by a sum of two Gaussian functions returns 6 parameters: the peak positions (PR_i, i =1,2) and standard deviations (σ_i, also referred to informally as the "width"), as well as the fraction of each population (f_i), shown on the right plots (**B** & **D**). Increasing the size threshold results in decreased PR histogram widths for both components (open symbols, dashed curves), but little changes in the peak positions (full symbols, plain curves). Dashed curves are constant fits to the peak positions or exponential fits to the peak standard deviations. Red: FRET peak, green: donor-only peak.

The previous conclusions hold whatever search type is used (minimum SBR criterion or fixed minimum count rate threshold), and whatever burst selection criterion is used, including imposing a minimum γ-corrected burst size or a minimum peak burst count rate. This similar behavior is due to the strong correlation between all these observables (Fig. SI-14E, F).

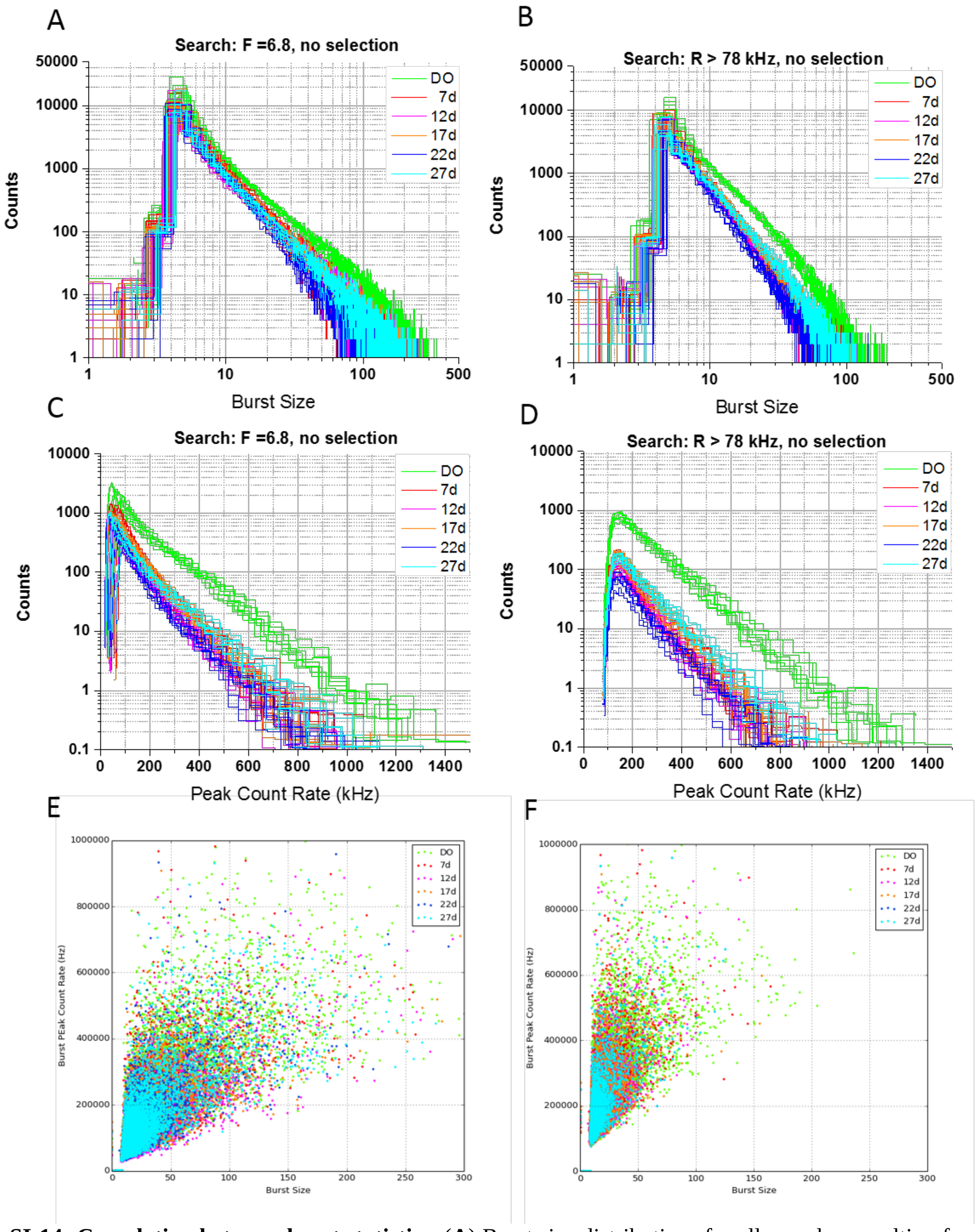

Fig. SI-14: Correlation between burst statistics. (**A**) Burst size distributions for all samples, resulting from an m = 5, F = 6.8 burst search without further selection. There are 8 distributions per sample. (**C**) Corresponding total peak count rate distributions. (**B**) Burst size distributions for all samples, resulting from an m = 5, $R \geq 78$ kHz burst search without further selection. There are 8 distributions per sample. (**D**) Corresponding total peak count rate distributions. (**E**) Scatterplots of total peak count rate versus burst size for spot 4. The same search (without selection) as in (A, C) was performed. (**F**) Scatterplots of total peak count rate versus burst size for spot 4. The same search (without selection) as in (B, D) was performed.

An alternative approach consists in using a "weighted" *PR* histogram, where each burst is counted not as one unit, but as a number related to its burst size. Formally, the weight factor can be expressed as a function *f* of the background-corrected burst size $\overline{F}$ (Appendix S6 of ref. [22], doi:10.1371/journal.pone.0160716.s006):

$$\overline{F} = \overline{F}_D^G + \overline{F}_D^R + \overline{F}_A^R \ . \qquad \text{(SI.38)}$$

The weighted histogram can be defined formally by its bin contents h_i:

$$\begin{aligned} h_i &= \frac{N}{N_f} \sum_{\substack{\text{burst } j \\ PR_j \in \text{bin } i}} f\left(\overline{F}_j\right) \\ N &= \sum_{\text{all bursts } j} 1 \\ N_f &= \sum_{\text{all bursts } j} f\left(\overline{F}_j\right) \end{aligned} \qquad \text{(SI.39)}$$

where the ith histogram bin boundaries are those of the regular (unweighted) histogram. As discussed in the reference cited above, the choice *f(x) = x* has a simple statistical interpretation and is illustrated in Fig. SI-15, for the same sample and observation spot as used in Fig. SI-13 (17d, spot 2).

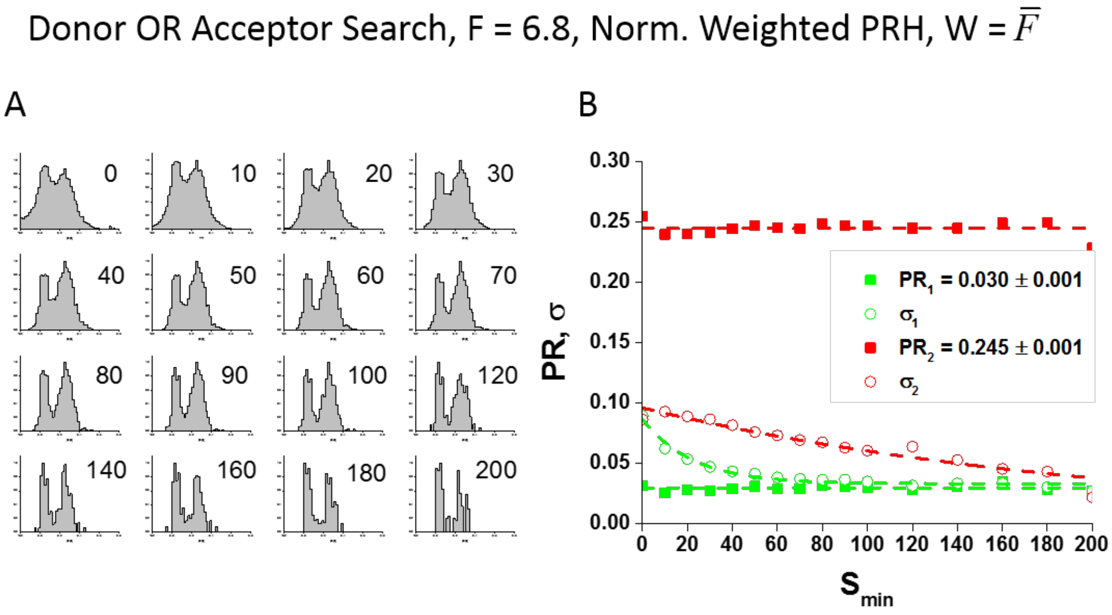

Fig. SI-15: Dependence of weighted proximity ratio statistics on the minimum burst size S_{min} (weight = $\overline{F}$). Same analysis as in Fig. SI-13, but using weighted *PR* histograms, each burst being associated with a weight equal to $\overline{F}$, where $\overline{F}$ is the total burst size. Notice the identical values of the peak PR, but somewhat reduced width of the *PR* histograms. The weighted *PR* histograms (A) show a clear separation between the two populations even without burst size selection (S_{min} = 0). (**A**) Normalized PRH, the horizontal axis covers [0, 1]. (**B**) Parameters of a 2-Gaussian Fit of the PRH.

Using a steeper function of the burst size as weight (e.g. $f(x) = x^2$), increasingly suppresses the influence of the smallest bursts on the weighted *PR* histogram (data not shown). This is easily seen by looking at the effect of varying the minimum burst size used to construct these histo-

grams (Fig. SI-15A). Loosely speaking, in this particular case, the first $\overline{F}$ -weighted *PR* histogram (S_{min} = 0 in Fig. SI-15A) is similar to the unweighted *PR* histogram with S_{min} = 30 (Fig. SI-13C).

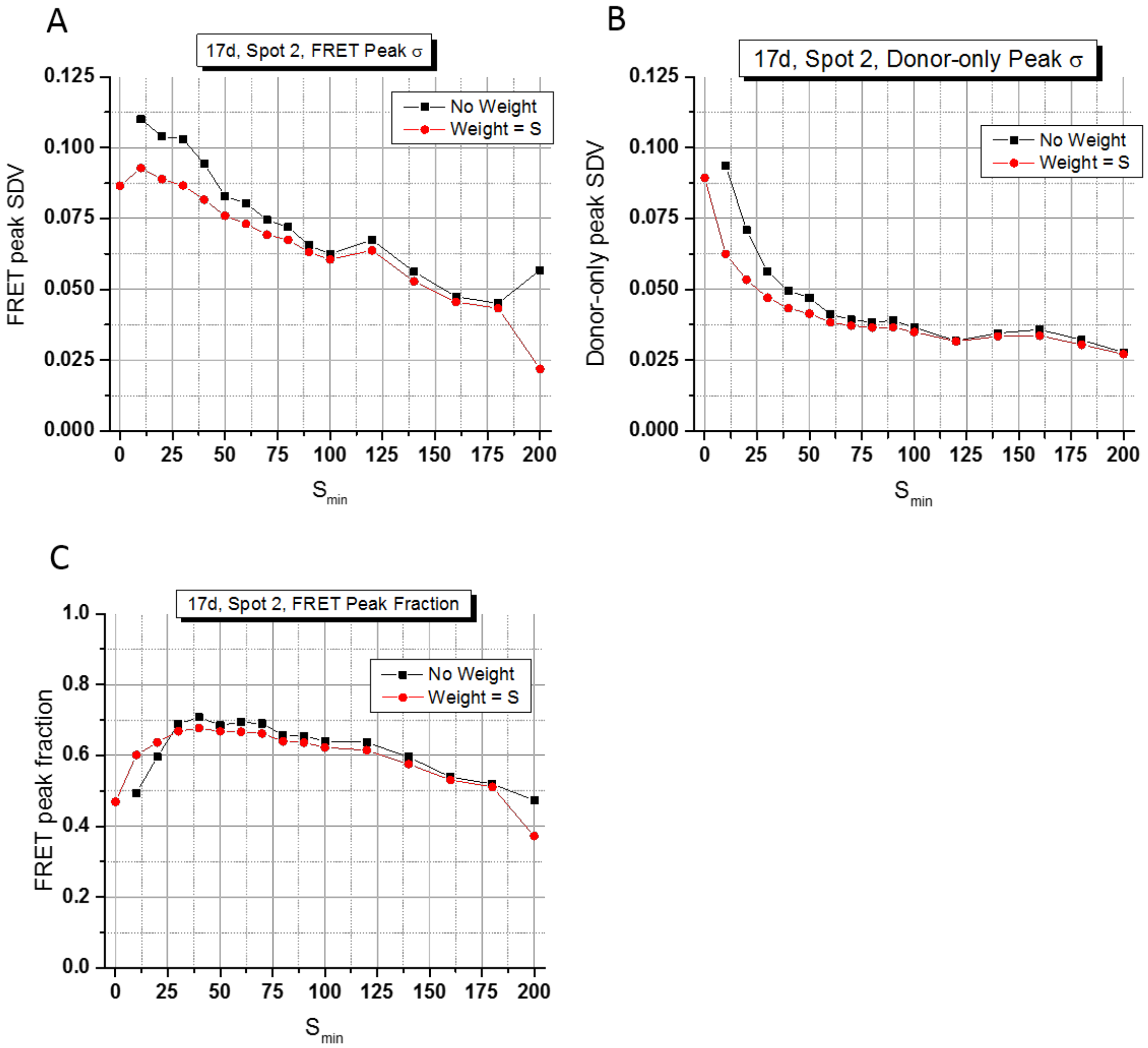

Fig. SI-16: Dependence of weighted proximity ratio statistics on the minimum burst size S_{min}. Results of the analyses presented in Fig. SI-13 & SI-15 (Donor OR Acceptor burst search). (**A**) Donor-only peak Gaussian fit parameter σ (width) as a function of burst size selection threshold S_{min} for different *PR* histogram weighting schemes; (**B**) FRET peak Gaussian fit parameter σ (width); (**C**) FRET peak fraction. The width of both peaks decreases as S_{min} increases, as expected from the reduction of shot noise broadening due to the rejection of small size bursts. Using a weighted *PR* histograms reduces both peak widths. The fraction of each population is dependent on S_{min}, with a decrease of the FRET fraction as the size selection threshold increases. This effect is due to the lower brightness of FRET bursts, itself due to the lower detection efficiency in the acceptor channel. Note that this effect is present even when a weighted *PR* histogram is used, and that the measured fraction depends on the weight chosen.

To quantify this comparison, the *PR* histograms can be fitted with a sum of two Gaussians and the evolution of their parameters studied as a function of S_{min} (Fig. SI-16). Each fit returns 6 parameters: the peak positions (PR_i, i =1,2) and standard deviations (σ_i, also referred to informally as the “width”), shown on the right plots in Fig. SI-13, as well as the fraction of each population (f_i) (f_1+ f_2 = 1). Increasing the size threshold results in decreasing PR histogram widths, with no variation of the peaks positions.

Appendix 9 Correction Factors

9.1 Theory

Four main corrections need to be applied on raw burst counts, in order to compute accurate FRET efficiency:

1. a stream-specific background correction,
2. a correction of the D-excitation R-detection stream for D-emission leakage in the R-channel,
3. a correction of the same stream for direct excitation of the acceptor by the D-excitation laser,
4. a correction for the difference in detection efficiency of G- and R-channels and quantum yields of the donor and acceptor species (γ factor correction).

We now briefly recall how the corresponding corrections and factors are computed [30]. A collection of similar derivations using notations used in FRETBursts notebooks can be found in ref. [31]. Background corrections are performed as described in Appendix 6.

9.1.1 Donor Leakage Factor

The donor leakage factor l is obtained as the parameter resulting in an l-corrected D-only (DO) proximity ratio histogram centered around 0. Using the most likely value PR_{DO} of the uncorrected proximity ratio of the DO population (see Appendix 10), the relation between PR_{DO} and l is:

$$PR_{DO} = \frac{l}{1+l} \qquad l = \frac{PR_{DO}}{1-PR_{DO}} \tag{SI.40}$$

Alternatively, l can be defined as the parameter resulting in a D-only, leakage-corrected acceptor channel signal:

$$\bar{F}_D^R - l \times \bar{F}_D^G \left(= \bar{F}_D^R - Lk \right) \tag{SI.41}$$

centered around 0.

9.1.2 Acceptor Direct Excitation Factor

The acceptor direct excitation factor d can be obtained as the parameter resulting in an A-only (AO) uncorrected stoichiometry ratio centered around 0. Using the A-only, uncorrected stoichiometry ratio SR_{AO}, the relation between SR_{AO} and d is:

$$SR_{AO} = \frac{d}{1+d} \qquad d = \frac{SR_{AO}}{1-SR_{AO}} \tag{SI.42}$$

Alternatively, d can be defined as the parameter resulting in a direct excitation-corrected A-only, D-excitation, acceptor channel signal:

$$\bar{F}_D^R - d \times \bar{F}_A^R \left(= \bar{F}_D^R - Dir \right) \tag{SI.43}$$

centered around 0.

We will introduce the following notation using a tilde symbol (~), for the D-excitation, A-channel signal corrected for background, leakage and acceptor direct excitation:

$$\tilde{F}_D^R = \bar{F}_D^R - l \times \bar{F}_D^G - d \times \bar{F}_A^R \left(= \bar{F}_D^R - Lk - Dir \right) . \qquad \text{(SI.44)}$$

In particular, for FRET species, this quantity will be noted $\tilde{F}_{FRET}$.

In order to use consistent notations, we will also introduce $\tilde{F}_D^G = \bar{F}_D^G$ and $\tilde{F}_A^R = \bar{F}_A^R$ (there is no additional correction for these specific streams beside background correction).

9.1.3 The γ Factor

The γ factor is obtained as described in Lee *et al.* [30], using the mean corrected proximity ratio E_{PR} and mean corrected stoichiometry ratio S of the series of dsDNA FRET samples described in Section 4 of the main text.

As a reminder, the *l*- and *d*-corrected proximity ratio E_{PR} and the *l*- and *d*-corrected stoichiometry ratio S are defined as follows:

$$\begin{aligned} E_{PR} &= \frac{\tilde{F}_{FRET}}{\tilde{F}_D^G + \tilde{F}_{FRET}} \\ S &= \frac{\tilde{F}_D^G + \tilde{F}_{FRET}}{\tilde{F}_D^G + \tilde{F}_{FRET} + \tilde{F}_A^R} \end{aligned} \qquad \text{(SI.45)}$$

Using the most likely values of these two quantities for the FRET subpopulation of each sample, $\{(E_{PR,i}, S_i)\}$ and fitting the following relation to this set of points:

$$1/S = \omega + \sigma \times E_{PR} , \qquad \text{(SI.46)}$$

one obtains experimental parameters ω and σ, from which the γ factor can be computed as:

$$\gamma = \frac{\omega - 1}{\sigma + \omega - 1} , \qquad \text{(SI.47)}$$

and the associated factor β:

$$\beta = \sigma + \omega - 1 . \qquad \text{(SI.48)}$$

The corrected FRET efficiency E and stoichiometry S_γ for each sample are then obtained using the following relations:

$$\begin{aligned} E &= \frac{E_{PR}}{\gamma - (\gamma - 1) E_{PR}} \\ S_\gamma &= \frac{\gamma S}{1 + (\gamma - 1)\left(E + (1 - E) S\right)} = \frac{S\left(\gamma + (1-\gamma) E_{PR}\right)}{1 + S(1-\gamma)\left(E_{PR} - 1\right)} \end{aligned} \qquad \text{(SI.49)}$$

9.2 Single-Spot µs-ALEX Experiments

9.2.1 Donor Leakage Factor

Donor leakage factors were obtained from analysis of the donor-only (D-only) population *PR* histogram of each sample, using three different approaches:

i. fit with a normal distribution;
ii. analysis by kernel density estimation (KDE), yielding the peak value, which was converted into a leakage factor using Eq. (SI.40)

iii. shot noise analysis (SNA) of the same *PR* was performed assuming an underlying Gaussian distribution of FRET efficiency with center and standard deviation (E, σ_E). Eq. (SI.40) was used with $PR_{DO} = E$ to obtain the leakage factor. In this latter calculations, the best fit parameters was $\sigma_E = 0$, indicating that the *PR* distribution was shot noise limited.

Table SI-3 reports the results of these analyses, which show a negligible negative bias of the standard *PR* histogram analysis results compared to shot noise analysis. The average value of the KDE results was used in subsequent analyses. Details of the analysis can be found in the μs-ALEX: Leakage coefficient section of the main Jupyter notebook [7].

	7d	12d	17d	22d	27d	All
Normal	0.106	0.106	0.111	0.096	0.098	0.103 ± 0.006
KDE	0.098	0.106	0.111	0.092	0.089	0.099 ± 0.009
SNA	0.115	0.115	0.120	0.110	0.104	0.113 ± 0.006
# Bursts	702	412	604	394	184	459 ± 201

Table SI-3: Donor-leakage factors obtained using kernel density analysis (KDE) or shot noise analysis (SNA) of the D-only *PR* histogram. Burst used for the analysis were obtained by an APBS $m = 5$, $F = 6.8$ search, $\overline{F} \geq 30$, $PR \leq 0.3$, $SR \geq 0.85$ selection criteria. Their number is indicated in the last row. All: mean and sample standard deviation of all samples. Results obtained using ALiX Scripts provided in ref. [8].

9.2.2 Acceptor Direct Excitation Factor

A similar approach could in principle be used for the acceptor direct excitation factor provided enough acceptor-only (A-only) bursts are detected in each sample. Since this was not the case, the A-only bursts of all samples (APBS $m = 5$, $F = 6.8$ search, $\overline{F}_A^R \geq 30$, $\overline{F}_D^G \leq 0$, $SR \leq 0.2$ selection criteria) were accumulated and their *SR* data was analyzed by normal fitting and kernel density estimation. The peak position ($SR = 0.044$ (Normal fit), 0.041 (KDE)) was converted into a acceptor direct excitation factor using Eq. (SI.42), resulting in a acceptor direct excitation factor of 0.045. Note that the value quoted in the main text ($d = 0.06$) corresponds to a different set of search parameters (A_{em}BS, $m = 10$, $F = 7$, $\overline{F}_A^R > 30$). The difference is small but points to the fact that it is difficult to obtain correction factors with an absolute precision of better than a few percents.

In general advisable to use dedicated D-only and A-only samples to estimate these correction factors reliably.

9.2.3 γ Factor

The γ factor was obtained as described in Section 9.1.3, resulting in a value $\gamma = 1.02$.

9.3 Multispot Experiments

9.3.1 Donor Leakage Factors

Leakage factors were measured as described, using 3 different methods. The results are presented in Fig. SI-17 and Table SI-4 to SI-5, and discussed in the main text.

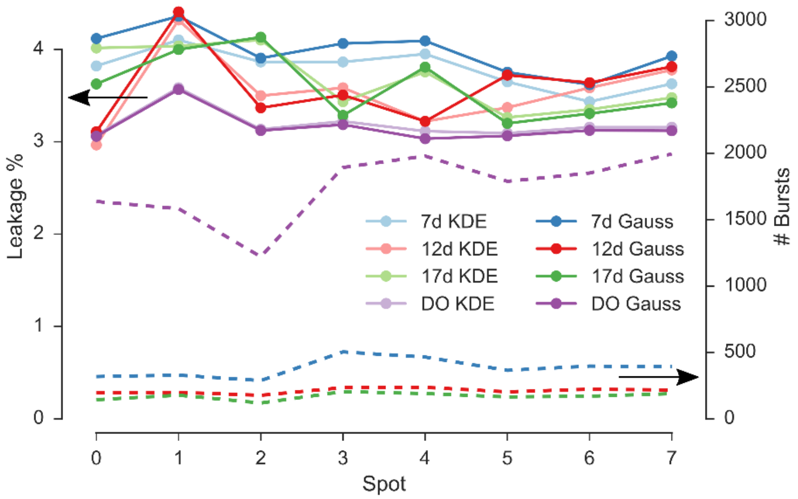

Fig. SI-17. Leakage factor versus spot computed with different fitting methods in different samples. Leakage factor (*solid lines*, *left vertical axis*) estimated for each spot in 4 dsDNA samples (7d, 12d, 17d and DO). The leakage coefficient is computed from the DO peak position in the background-corrected *PR* distribution. The DO peak position is estimated either by kernel density estimation (*label KDE, dark colors*) or using a Gaussian fit (*label Gauss, light colors*). *Dashed lines*, *right vertical axis*: number of bursts in the DO population for the different samples (colors are identical to those used for the leakage factor). For computational details (including the numerical values used in the above figure) see section Leakage coefficient of the main Jupyter notebook [7].

Spot	1	2	3	4	5	6	7	8	All
Gaussian	0.046	0.137	0.045	0.044	0.048	0.054	0.048	0.044	0.058 ± 0.014
KDE	0.023	0.103	0.030	0.034	0.034	0.040	0.028	0.029	0.040 ± 0.026
SNA	0.054	0.140	0.044	0.048	0.046	0.050	0.058	0.058	0.062 ± 0.032
# Bursts	425	463	361	623	599	464	546	513	499 ± 88

Table SI-4: Multispot Measurement. Donor-leakage coefficients obtained for the 7d sample using Gaussian fit, kernel density analysis (KDE) or shot noise analysis (SNA, 1 replicas per burst). Bursts used for the analysis were obtained by a D_{em} burst search with $m = 10$, $F = 6$, and $\overline{F}_D^D \geq 80$ selection criterion. All: mean (sample standard deviation) of all spots. The values obtained by pooling bursts from all 8 spots (instead of performing a spot by spot analysis) were $l = 0.051$ (Gaussian fit), $l = 0.032$ (KDE) and $l = 0.068$ (SNA).

Spot	1	2	3	4	5	6	7	8	All
Gaussian	0.040	0.111	0.043	0.036	0.042	0.039	0.045	0.046	0.050 ± 0.025
KDE	0.017	0.075	0.030	0.031	0.034	0.022	0.030	0.037	0.034 ± 0.018
SNA	0.028	0.11	0.036	0.040	0.036	0.046	0.038	0.040	0.047 ± 0.026
# Bursts	233	235	206	270	287	247	261	247	248 ± 25

Table SI-5: Multispot Measurement. Donor-leakage coefficients obtained for the 12d sample using Gaussian fit, kernel density analysis (KDE) or shot noise analysis (SNA, 1 replica per burst). Bursts used for the analysis were obtained by a D_{em} Burst Search, $m = 10$, $F = 6$, $\overline{F}_D^D \geq 80$ and $PR \leq 0.3$ selection criteria. All: mean (sample standard deviation) of all spots. The values obtained by pooling bursts from all 8 spots (instead of performing a spot by spot analysis) were $l = 0.046$ (Gaussian fit), $l = 0.033$ (KDE) and $l = 0.048$ (SNA).

Spot	1	2	3	4	5	6	7	8	All
Gaussian	0.030	0.067	0.035	0.034	0.034	0.033	0.019	0.034	0.036 ± 0.014
KDE	0.030	0.064	0.029	0.030	0.026	0.029	0.028	0.028	0.032 ± 0.013
SNA	0.026	0.050	0.032	0.032	0.028	0.028	0.030	0.030	0.032 ± 0.008
# Bursts	1618	1571	1197	1852	1952	1760	1819	1958	1716 ± 252

Table SI-6: Multispot Measurement. Donor-leakage coefficients obtained for the DO sample using Gaussian fit, kernel density analysis (KDE) or shot noise analysis (SNA, 1 replica per burst). Bursts used for the analysis were obtained by a D_{em} Burst Search, $m = 10$, $F = 6$, $\bar{F}_D^D \geq 80$ and $PR \leq 0.26$ selection criteria. All: mean (sample standard deviation) of all spots. The values obtained by pooling bursts from all 8 spots (instead of performing a spot by spot analysis) were $l = 0.036$ (Gaussian fit), $l = 0.029$ (KDE) and $l = 0.034$ (SNA).

9.3.2 Acceptor Direct Excitation Factor(s)

In the absence of acceptor excitation laser, the contribution of the direct acceptor excitation of the acceptor by the donor laser cannot be corrected using Eq. (SI.44). As shown in ref. [30], it is for instance possible to express it as a function of the donor signal coming from direct excitation by the donor laser, $\bar{F}_D^G$ [5]. The expression provided there (Eq. (27) in ref. [30]) was derived for a *doubly-labeled, zero-FRET sample*, for which:

$$Dir = d'\bar{F}_D^G \left(= d'\tilde{F}_D^G\right) , \qquad \text{(SI.50)}$$

with:

$$d' = \gamma \frac{\sigma_D^A}{\sigma_D^D} , \qquad \text{(SI.51)}$$

where σ_X^Y is the absorption cross-section of species Y at the wavelength of excitation laser X. Formally, using the notations of ref. [30], it is possible to express *Dir* as:

$$Dir = I_D \sigma_D^A \phi_A \eta_A^R . \qquad \text{(SI.52)}$$

Using the definitions of the corrected donor and acceptor signals, $\tilde{F}_D^G \left(= \bar{F}_D^G\right)$ and $\tilde{F}_D^R$ (Eq. (SI.99)), one obtains:

$$Dir = d_T \left(\gamma \tilde{F}_D^G + \tilde{F}_D^R\right), \qquad \text{(SI.53)}$$

with:

$$d_T = \sigma_D^A / \sigma_D^D . \qquad \text{(SI.54)}$$

[5] In the main text, this quantity is called n_{Dem} (or n_D). Quantity n_{Aem} (or n_A) in the main text corresponds to $\bar{F}_D^R$ in this document. $\bar{F}_A^R$ corresponds to n_{AA}.

Eq. (SI.53) expresses *Dir* as a function of the γ-corrected burst size (Eq. (SI.101)) and is valid forany doubly-labeled molecules[6]. The direct excitation coefficient d_T is an intrinsic property of the dye pair, being the ratio of D and A absorption cross-sections at the D excitation wavelength. Since Eq. (SI.53) involves the leakage and direct-excitation corrected quantity $\tilde{F}_D^R$ (Eq. (SI.44)), we can rewrite it in a way that isolates *Dir*, to obtain an expression involving only background-corrected burst quantities $\bar{F}_D^G \left(= \tilde{F}_D^G\right)$ and $\bar{F}_D^R$:

$$Dir = \frac{d_T}{1+d_T}\left(\gamma \bar{F}_D^G + \left(\bar{F}_D^R - l\bar{F}_D^G\right)\right). \quad \text{(SI.55)}$$

Note that for small values of parameters d_T, this expression will in practice differ very little from Eq. (SI.53) where $\tilde{F}_D^R$ is replaced by the leakage-only corrected quantity $\bar{F}_D^R - l\bar{F}_D^G$.

Another expression for *Dir* can be obtained as a function of the background-corrected burst size $\bar{F}_D = \bar{F}_D^G + \bar{F}_D^R$ (defined in Eq. (SI.35)):

$$Dir = \frac{d'}{(1+l)(1-E)+\gamma E + d'}\bar{F}_D , \quad \text{(SI.56)}$$

This relation is used to estimate the effect of direct acceptor excitation in Shot Noise Analysis (Appendix 11). Other relations between *Dir* and different burst quantities can be found in ref. [31].

Using factor d_T, the FRET efficiency *E* can be expressed as:

$$E = \frac{1-(1+l+\gamma d_T)(1-PR)}{1-(1+l-\gamma)(1-PR)} , \quad \text{(SI.57)}$$

which involves correction factor γ, D-leakage factor *l* (both spot-specific), the A-direct excitation factor d_T and the proximity ratio *PR* calculated without any leakage or direct excitation correction (Eq. (SI.36), E_{PR}^{raw} in ref. [30]).

The A-direct excitation factor $d_T = \sigma_D^A / \sigma_D^D$ can in principle be computed from the normalized absorption spectra of the donor and acceptor dye (measured in the environment they are found in in the sample) at wavelength λ_D, $\mathcal{A}_D(\lambda_D)$ and their respective extinction coefficients ε_D and ε_A:

$$d_T = \frac{\sigma_D^A}{\sigma_D^D} = \frac{\mathcal{A}_A(\lambda_D)}{\mathcal{A}_D(\lambda_D)}\frac{\varepsilon_A}{\varepsilon_D} , \quad \text{(SI.58)}$$

[6] Eq. (SI.50) only applies to zero-FRET samples. For a *doubly-labeled sample characterized by a non-zero FRET efficiency E*, it needs to be modified into:

$$Dir = \frac{d'}{1-E}\tilde{F}_D^G \left(= \frac{d'}{1-E}\bar{F}_D^G\right).$$

This expression reduces to Eq. (SI.50) for *E* = 0, but is indeterminate for a 100% FRET efficiency sample (*E* = 1), for which $\bar{F}_D^G \sim 0$. In this case, Eq. (SI.55) is a preferable expression. Obviously, a donor-only molecule will not contribute any direct acceptor excitation signal (*i.e. Dir* = 0 for these molecules).

where λ_D is the donor excitation laser wavelength.

Alternatively, since d_T is a characteristic of the sample, not of the measurement method, it can be obtained from μs-ALEX measurements. Simple algebra shows that:

$$d_T = \beta d \ , \qquad \text{(SI.59)}$$

where parameter β is defined by Eq. (SI.104) and d is the direct acceptor excitation factor defined in 9.1.2.

While d_T can be obtained from μs-ALEX measurements, the γ factor to be used in all multispot calculations is that characterizing the multispot setup, γ_m. In particular, when using parameter d' (e.g. in Eq. (SI.56)), the following formula applies:

$$d_m' = d_{T,s}\,\gamma_m \ , \qquad \text{(SI.60)}$$

where the subscripts s and m indicate which measurement the parameter is obtained from (s: single-spot μs-ALEX measurement, m: multispot, non μs-ALEX measurements).

9.3.3 γ Factor

In multispot experiments the absence of acceptor laser excitation prevents determining γ_m with the procedure used for μs-ALEX data. The value has been instead determined by comparison of PR for one sample (chosen as calibration sample) with the γ-corrected FRET efficiency obtained from μs-ALEX measurement of the same sample. Formally, γ_m can be computed as:

$$\gamma_m = \frac{1-E_s}{d_T + E_s} \cdot \frac{(1+l_m)PR_m - l_m}{1-PR_m} \ , \qquad \text{(SI.61)}$$

where the subscripts s and m indicate the setup with which the quantity is measured (s: single-spot μs-ALEX, m: multispot setup). Parameter d_T (Eq. (SI.54)) is a characteristic of the sample and can be computed from single-spot μs-ALEX measurements. Eq. (SI.61) is obtained from Eq. (SI.57), by solving for γ. As discussed next, it is possible to determine the value PR_m minimizing the uncertainty on γ_m. In this work, sample 12d was used for that purpose.

Note that the multispot γ factor (γ_m) could in principle be estimated on a spot-by-spot basis. This could be necessary if the PR peak position showed significant variation across channels, which was not the case for the measurements reported in this work. For computational details of how the γ_m coefficient is computed see section PR and FRET analysis of the main Jupyter notebook.

9.3.4 Error on factor γ estimation

Differentiating Eq. (SI.61) with respect to its two dependent variable l and PR, we obtain:

$$\operatorname{var}(\gamma) = \left(\frac{\partial\gamma}{\partial l}\right)^2 \operatorname{var}(l) + \left(\frac{\partial\gamma}{\partial PR}\right)^2 \operatorname{var}(PR) + 2\frac{\partial\gamma}{\partial l}\frac{\partial\gamma}{\partial PR}\operatorname{cov}(l, PR) \ , \qquad \text{(SI.62)}$$

with obvious notations. Assuming independence between the estimates of l and PR:

$$\begin{aligned}\operatorname{var}(\gamma) &= (1-PR)^{-4}\left(\frac{1-E_s}{d_T+E_s}\right)^2\left(\operatorname{var}(PR) + (1-PR)^4\operatorname{var}(l)\right) \\ &= K^2(E_s, PR, d_T)\left(\operatorname{var}(PR) + (1-PR)^4\operatorname{var}(l)\right)\end{aligned} \ . \qquad \text{(SI.63)}$$

Any *PR* variance will clearly be magnified for *PR* values close to 1, while any value of E_s small enough will amplify the right hand side of Eq. (SI.63) due to the factor $(d_T + E_s)^{-2}$, where d_T is in general small.

In the case where the term involving the variance of *l* is negligible compared to the term involving the variance of *PR*, and assuming that $PR \sim E_s \sim x$, the error on γ will minimal when $\mathrm{Var}(PR)^{1/2} \times \left[(d_T + x)(1-x)\right]^{-1}$ is minimal. Assuming furthermore (which is the case here) that $d_T \ll 1$, the minimum of $\left[x(1-x)\right]^{-1}$ is obtained for $x = ½$. In other words, in order to minimize the error on the estimation of factor γ using Eq. (SI.61), a sample with FRET efficiency close to ½ is preferable, assuming that var(*PR*) is similar for all samples. Using the above approximations, the error on this estimate is given by:

$$\sigma_\gamma \sim \frac{1-E_s}{E_s}(1-PR)^{-2}\sigma_{PR} \ , \qquad \text{(SI.64)}$$

where σ_X designates the standard deviation of *X*.

In general, when var(*PR*) and var(*l*) are similar for all samples, the sample minimizing the uncertainty on factor γ is the one minimizing *K* in Eq. (SI.63):

$$K(E_s, PR, d_T) = (1-PR)^{-2}\left(\frac{1-E_s}{d_T + E_s}\right) \qquad \text{(SI.65)}$$

9.3.5 Proximity Ratios

Proximity ratios estimated by single peak Gaussian fit, KDE analysis and SNA analysis are reported below for each sample. Details on SNA analysis can be found in Appendix 11. The values reported here are those obtained using the ALiX scripts provided in [8]. For similar values computed with the FRETBursts software see section PR and FRET analysis of the main Jupyter notebook [7].

Spot	1	2	3	4	5	6	7	8	All
Gaussian	0.87	0.86	0.83	0.84	0.83	0.85	0.84	0.85	0.85 ± 0.01
KDE	0.88	0.87	0.84	0.85	0.84	0.86	0.84	0.85	0.85 ± 0.01
SNA	0.91	0.90	0.86	0.88	0.88	0.90	0.88	0.88	0.82 ± 0.01
# Bursts	548	625	515	793	768	253	1040	1269	726 ± 318

Table SI-7: Multispot Measurement. PR values obtained for the 7d sample using Gaussian fit, kernel density analysis (KDE) or shot noise analysis (SNA, mean value, 1 replica per burst). Bursts used for the analysis were obtained by an APBS m = 10, F = 7 search, $\bar{F} \geq 30$ and $PR \geq 0.5$ selection criteria. All: mean ± sample standard deviation of all spots. The values obtained by pooling bursts from all 8 spots (instead of performing a spot by spot analysis) were *PR*= 0.85 (Gaussian fit), *PR* = 0.85 (KDE) and *PR* = 0.82 ± 0.05 (SNA: mean ± standard deviation). Data computed using ALiX Scripts 6-8.

Spot	1	2	3	4	5	6	7	8	All
Gaussian	0.59	0.60	0.57	0.57	0.56	0.55	0.56	0.56	0.57 ± 0.02
KDE	0.58	0.59	0.55	0.54	0.54	0.52	0.55	0.55	0.55 ± 0.02
SNA	0.57	0.58	0.55	0.55	0.54	0.54	0.55	0.55	0.55 ± 0.01
# Bursts	958	1062	1160	1532	1439	817	1617	1644	1279 ± 320

Table SI-8: Multispot Measurement. PR values obtained for the 12d sample using Gaussian fit, kernel density analysis (KDE) or shot noise analysis (SNA, mean value, 1 replica per burst). Bursts used for the analysis were obtained by an APBS m = 10, F = 7 search, $\overline{F} \geq 30$ and $PR \geq 0.24$ selection criteria. All: mean ± sample standard deviation of all spots. The values obtained by pooling bursts from all 8 spots (instead of performing a spot by spot analysis) were PR = 0.57 (Gaussian fit), PR = 0.56 (KDE) and PR = 0.56 ± 0.05 (SNA:: mean ± standard deviation). Data computed using ALiX Scripts 9-11.

Spot	1	2	3	4	5	6	7	8	All
Gaussian	0.30	0.31	0.28	0.28	0.29	0.29	0.28	0.29	0.29 ± 0.01
KDE	0.29	0.30	0.26	0.27	0.28	0.27	0.26	0.26	0.27 ± 0.02
SNA	0.29	0.29	0.27	0.28	0.29	0.28	0.27	0.28	0.28 ± 0.01
# Bursts	505	505	496	663	676	391	757	811	600 ± 147

Table SI-9: Multispot Measurement. PR values obtained for the 17d sample using Gaussian fit, kernel density analysis (KDE) or shot noise analysis (SNA, mean value, 1 replica per burst). Bursts used for the analysis were obtained by an APBS m = 10, F = 7 search, $\overline{F} \geq 50$ and $PR \geq 0.12$ selection criteria. The larger burst size threshold was used in order to better separate the D-only population from the FRET population. All: mean ± sample standard deviation of all spots. The values obtained by pooling bursts from all 8 spots (instead of performing a spot by spot analysis) were PR = 0.28 (Gaussian fit), PR = 0.27 (KDE) and PR = 0.29 ± 0.03 (SNA). Data computed using ALiX Scripts 12-14.

Spot	1	2	3	4	5	6	7	8	All
Gaussian	0.12	0.15	0.10	0.11	0.10	0.13	0.11	0.11	0.12 ± 0.02
KDE	0.11	0.14	0.08	0.08	0.07	0.11	0.08	0.07	0.09 ± 0.02
SNA	0.11	0.14	0.09	0.10	0.09	0.11	0.09	0.09	0.10 ± 0.02
# Bursts	876	825	946	1310	1092	744	1243	1386	1053 ± 240

Table SI-10: Multispot Measurement. PR values obtained for the 22d sample using Gaussian fit, kernel density analysis (KDE) or shot noise analysis (SNA, mean value, 1 replicas per burst). Bursts used for the analysis were obtained by an APBS m = 10, F = 7 search, $\overline{F} \geq 30$ selection criterion. All: mean ± sample standard deviation of all spots. The values obtained by pooling bursts from all 8 spots (instead of performing a spot by spot analysis) were PR = 0.11 (Gaussian fit), PR = 0.09 (KDE) and PR = 0.10 ± 0.04 (SNA). Data computed using ALiX Scripts 15-17.

Spot	1	2	3	4	5	6	7	8	All
Gaussian	0.10	0.13	0.09	0.09	0.09	0.10	0.09	0.09	0.10 ± 0.01
KDE	0.08	0.10	0.07	0.07	0.07	0.08	0.07	0.08	0.08 ± 0.01
SNA	0.09	0.12	0.08	0.08	0.08	0.08	0.08	0.08	0.08 ± 0.01
# Bursts	1459	1370	1721	2063	2111	1498	2147	2286	1832 ±361

Table SI-11: Multispot Measurement. PR values obtained for the 27d sample using Gaussian fit, kernel density analysis (KDE) or shot noise analysis (SNA, mean value, 1 replicas per burst). Bursts used for the analysis were obtained by an APBS m = 10, F = 7 search, $\overline{F} \geq 30$ selection criterion. All: mean ± sample standard deviation of all spots. The values obtained by pooling bursts from all 8 spots (instead of performing a spot by spot analysis) were PR = 0.09 (Gaussian fit), PR = 0.07 (KDE) and PR = 0.08 ± 0.02 (SNA). Data computed using ALiX Scripts 18-20.

9.4 Multispot vs single-spot FRET histogram comparison

A limitation of the current multi-spot system is related to the lower PDE of SPAD arrays in the red region of the spectrum (> 600 nm) compared to single-pixel SPADs.

For the current system, direct comparison of FRET histograms of the single-spot and multi-spot measurements is not trivial. In principle, we should compare the single-spot histogram acquired over a given duration with the multispot histogram obtained by pooling bursts from 8 channels covering ⅛ of that duration. However, two observations make this comparison less than ideal. First, the measurements are not performed with the same sample concentrations (they are from different dilution batches) therefore burst rates are not directly comparable between the two experiments. Second, the background is higher in the multispot setup, in part because of the multi-spot excitation scheme itself, but also because of the noisier SPAD arrays compared to single-pixel SPADs. For FRET efficiency estimation we used a constant SBR burst search, that is, a burst search depending on the background rate. This helps obtaining FRET histograms with fixed shot-noise level, regardless of the background. However, since the background is higher in the multispot setup, constant-SBR burst search results in a higher threshold and consequently smaller number of bursts. The choice between a fixed-rate and fixed-SBR burst search is a trade-off between collecting more bursts (but containing more background) and fixing the allowed amount of background relative to signal (SBR). A discussion of this trade-off can be found in section 7.4 (Burst Search Influence on Burst Statistics) and in ref. [22].

Despite all these caveats, we provide here a qualitative comparison of FRET histograms in the single-spot and multi-spot measurements. For the multispot data, we pooled bursts from all 8 channels and limited the measurement duration to ⅛ of the µs-ALEX duration (600/8 s). The results are reported in Fig. SI-17.1. Both number of bursts and the fraction of D-only population vary between multi- and single-spot experiments, since the two measurements are performed on different days and sample dilutions. Nonetheless, we note a good agreement between the peak positions and widths of each measurement.

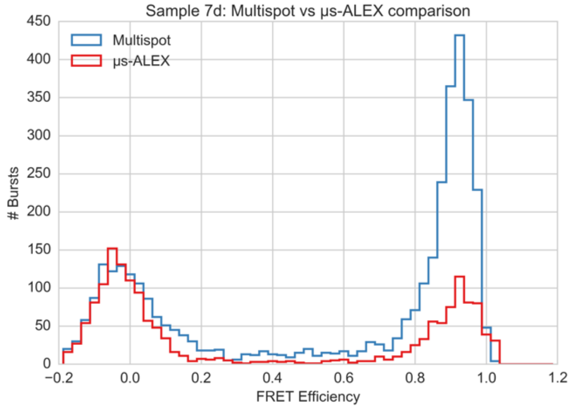

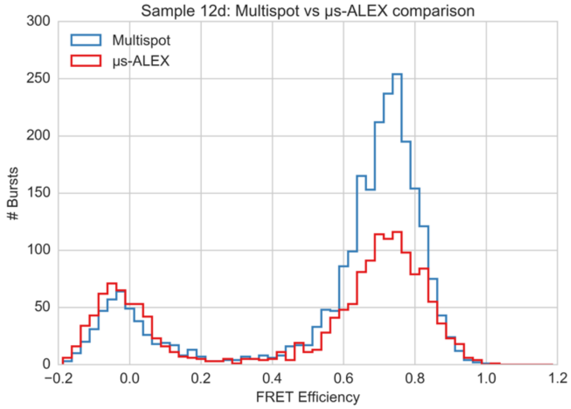

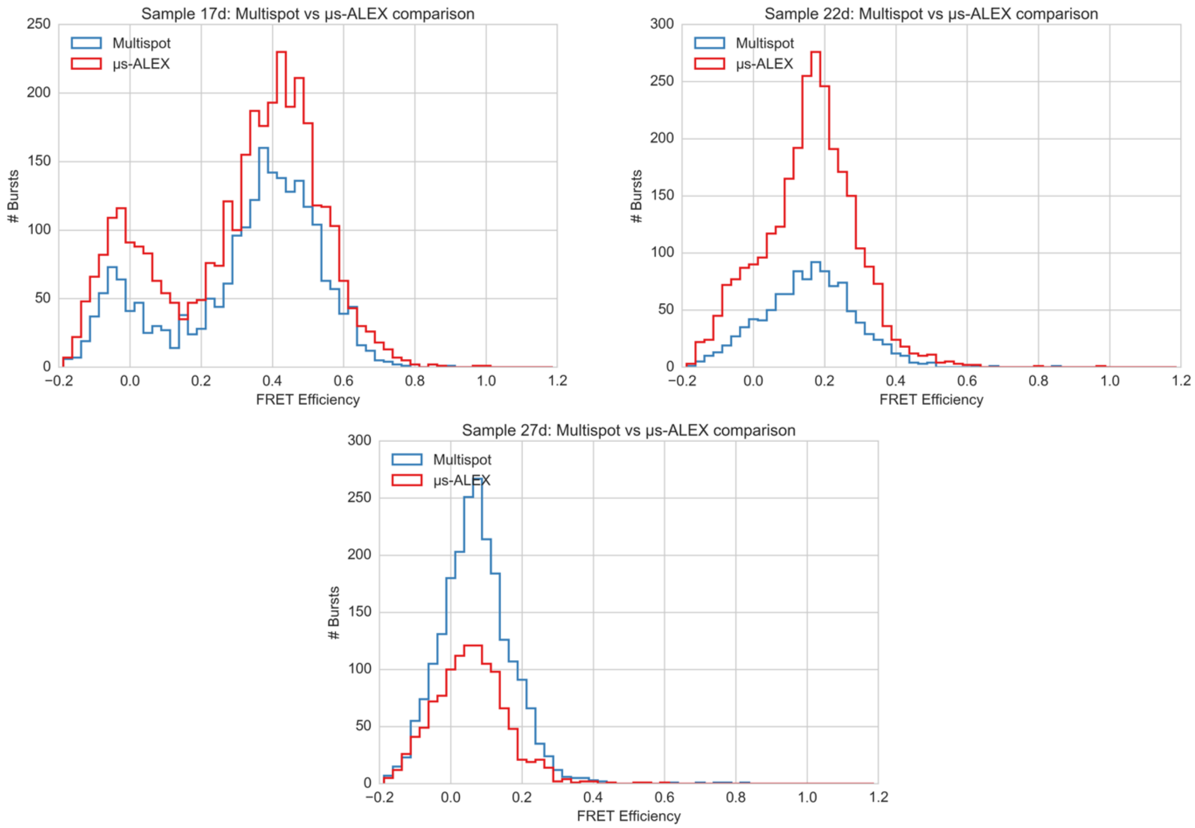

Fig. SI-17.1. Comparison of FRET histograms in the single-spot µs-ALEX and multispot setups. We report comparison for the same 5 dsDNA sample mentioned in the main text (Section 4). The multispot histogram is built by pooling bursts from all 8 channels with a measurement duration equal to 1/8 the duration of the µs-ALEX measurements (600/8 s). Since the burst rate strongly depends on concentration and background level, the number of bursts cannot be quantitatively compared between the two kinds of measurement. For details, see section *Multispot vs usALEX FRET histograms* (view online) of the accompanying Jupyter notebook.

Appendix 10 Determination of Proximity Ratio and FRET Efficiency

A common problem in single-molecule burst analysis consists in determining the "characteristic" value of an observable for a selected burst sub-population. An example is provided by the proximity ratio (or the FRET efficiency) of a doubly-labeled population.

The common way of approaching this question has long been to compute a population-specific histogram of the observable and fit this histogram with an ad-hoc function (oftentimes a normal distribution) and report the function's peak position.

The first problem with this approach is that it is not always obvious which histogram bin size to choose for an optimal representation [24]. Secondly, a normal (Gaussian) distribution is not necessarily the best model function to fit the resulting histogram. While a Gaussian distribution might provide a reasonable fit for FRET efficiencies in the range $0.2 \leq E \leq 0.8$, asymmetric distributions are often encountered for small or large values of E. Finally, for asymmetric functions, it might not be obvious which quantity (mean, mode or median) to report in order to characterize the distribution.

Moreover, the distribution of an observable is affected by shot noise, whose magnitude depends on burst size. Small bursts generally result in asymmetric distribution for ratiometric observables (e.g. the proximity ratio), while large bursts may result in large variance for some observables (e.g. the ratio of donor and acceptor signal in a FRET sample). Since an experiment is characterized by a distribution of burst sizes, the overall effect on the distribution of the observable of interest can be complex, rendering the choice of a model function problematic. In particular, even with a knowledge of the effect of various burst quantity distributions (size, duration, etc.), it is rarely feasible to obtain a satisfactory “fit” of an observed histogram with a few parameters only.

For all these reasons, it would seem preferable to use a model-free approach.

The simplest model-free approach consists in reporting simple statistical measures of the observable such as the mean, median, mode, standard deviation, etc. However, these statistical measures can be biased or have a large variance. While these characteristics can be determined theoretically for model functions, incorporating shot noise effects in the equation rapidly makes this determination a complex problem.

Another model-free and almost parameter-free approach is provided by kernel density estimation (KDE) [32]. In KDE, a normalized function is used as kernel (typically a normal distribution) and scaled by a bandwidth parameter to represent each observable data point in the sample. The sum of these “replicas” of the kernel density is then taken as an approximation of the underlying probability distribution function (PDF) of the observable. The result can be described as a smoothed version of a standard histogram representation of the observable with a particular bandwidth. Because it is a continuous function, the median of the distribution can be computed unambiguously. However, because the bandwidth parameter affects the shape of the distribution, there is no guarantee that a mode value exists (there could be several local maxima in the KDE distribution) or, if it exists, that it is not biased.

All these methods (fit by *ad hoc* model functions, calculation of statistical measures, KDE analysis) ignore that, in practice, the observable distribution is due to an underlying distribution of molecular properties, convolved with an “instrument response function” (IRF) involving the whole optical setup and its detectors, the main feature of which is that observable values are obtained from small count numbers and therefore are affected by shot noise. The way to account for shot noise effects on burst observable is well understood, and involves taking into account the joint distribution of burst size and burst duration [24, 33]. There is some debate as to which additional parameters (molecular, setup characteristics) need to be added to the convolution in order to account for the observed distributions. Appendix 11 discusses these issues.

Appendix 11 Shot Noise Analysis

Shot noise analysis (also sometimes referred to as probability distribution analysis or PDA in the literature) [24, 33], in its simplest form used in this work, consists in comparing the measured proximity ratio histogram (PRH) to a predicted one based on the knowledge of burst counts, background rates and correction factors, and some adjustable model parameters describing the sample’s properties. Ideally, a single parameter, the FRET efficiency, should be needed to account for the PRH of a single, static population of doubly-labeled molecules. In practice, multiple sources of imperfection render this assumption too simplistic. Among others, the presence of D-only molecules, bleaching or blinking of the donor or acceptor molecules, dependence of the excitation and detection efficiencies on each molecule’s trajectory, and finally, possible distance

distributions or dynamic fluctuations, are some of the possible effects to take in to account in practical situations.

Here, we extend the model described in ref. [24] to:

- include the acceptor-excitation signal provided by µs-ALEX alternation (for correction of the FRET signal for direct excitation of the acceptor by the donor excitation laser)

- take into account the effect of a γ factor significantly different from 1 in the multispot measurements

- introduce a variant of the Gaussian distribution of distances to account for additional PRH broadening.

11.1.1 Using the acceptor excitation information in µs-ALEX measurements in SNA

In the Monte-Carlo approach used in ref. [24], each burst was characterized by its size *S* and duration τ, where *S* designates the total signal detected during donor excitation. Taking into account that there is a donor excitation period as well as an acceptor excitation period in µs-ALEX measurements, each burst can be characterized by its total signal during each excitation period, F_D and F_A, with:

$$F_X = \sum_{Y=R,G} F_X^Y \, . \qquad \text{(SI.66)}$$

With these notations, and using the definitions introduced in the main text, the algorithm used to generate a shot noise limited PRH based on the selected bursts is as follows:

(i) Choose an oversampling factor *N* (integer number) and a FRET efficiency ε,

(ii) For each burst, compute the $b_X^Y \Delta t$'s, where Δt is the burst duration.

(iii) Draw one random number *d* from the Poisson distribution of mean $b_D^G \Delta t$,

(iv) Draw one random number *a* from the Poisson distribution of mean $b_D^R \Delta t$,

(v) Draw one random number *r* from the Poisson distribution of mean $b_A^R \Delta t$,

(vi) Draw one random number *g* from the Poisson distribution of mean $b_A^G \Delta t$,

(vii) Draw one random number *Dir* from the Poisson distribution of mean $d(F_A - r - g)$ [direct acceptor excitation],

(viii) Draw one random number $\overline{F}_D^D$ from the binomial distribution of size $F_D - d - a - Dir$ and probability $(1-\varepsilon)/((1-\varepsilon)(1+l)+\gamma\varepsilon)$ [donor signal not due to direct excitation],

(ix) Define $N_D = \overline{F}_D^D + d$ and $N_A = (F_D - d) - \overline{F}_D^D$ [7],

(x) Add the value $(N_A - b_D^R \Delta t)/(F_D - (b_D^G + b_D^R)\Delta t)$ to the PRH[8],

(xi) Repeat (iii)-(ix) *N* times,

(xii) Repeat (ii)-(ix) for each burst,

(xiii) Divide the final PRH by *N*,

---

[7] By construction, $N_D + N_A = F_D$.

[8] At this stage, it is possible to apply the donor leakage and acceptor direct excitation corrections to obtain an E_{PR}-histogram rather than a PRH

(xiv) Compare the result to the measured PRH (*e.g.* using the mean square error) and improve ε.

When the FRET efficiency is modeled not by a single parameter, but several (as is the case when it is assumed that there is an underlying distribution of distances accounting for the observed PRH), the search for the optimal set of parameters was performed by a systematic exploration of the parameter space depending on the model chosen, as described in later sub-sections. The reason for this brute force approach is twofold:

- The range of parameters explored in this manner is well-circumscribed.
- The mean square error (MSE) map resulting from this approach is useful to gauge the quality of the minimum found.

Note that for PRH with poor statistics (small number of bursts, say, <100), choosing a large oversampling factor N is counterproductive, because the final PRH obtained in step (xii) is then much smoother than the actual PRH. On the other hand, when the PRH contains a large number of bursts (say, > 10,000), using a small N is recommended, due to the linear increase of computation time with N. A value N = 1 to 10 appears to be a good compromise.

11.1.2 Algorithm for shot noise analysis in multispot measurements

In the absence of an acceptor excitation laser, and of a γ factor markedly different from 1, we used a modified algorithm as follows, based on the only available burst size F_D:

(i) Choose an oversampling factor N (integer number) and a FRET efficiency ε,

(ii) For each burst, compute the $b_X^Y \Delta t$'s, where Δt is the burst duration.

(iii) Draw one random number d from the Poisson distribution of mean $b_D^G \Delta t$,

(iv) Draw one random number a from the Poisson distribution of mean $b_D^R \Delta t$,

(v) Draw one random number Dir from the Poisson distribution of mean $d'\left(F_D - d - a\right) / \left(\gamma\varepsilon + (1+l)(1-\varepsilon) + d'\right)$ [direct acceptor excitation, Eq. (SI.56)],

(vi) Draw one random number $\overline{F}_D^D$ from the binomial distribution of size $F_D - d - a - Dir$ and probability $(1-\varepsilon) / \left((1-\varepsilon)(1+l) + \gamma\varepsilon\right)$ [donor signal not due to direct excitation]

(vii) Define $N_D = \overline{F}_D^D + d$ and $N_A = \left(F_D - d\right) - \overline{F}_D^D$,

(viii) Add the value $\left(N_A - b_D^R \Delta t\right) / \left(F_D - \left(b_D^G + b_D^R\right)\Delta t\right)$ to the PRH,

(ix) Repeat (iii)-(ix) N times,

(x) Repeat (ii)-(x) for each burst,

(xi) Divide the final PRH by N,

(xii) Compare the result to the measured PRH (*e.g.* using the mean square error) and improve ε.

11.1.3 Normal FRET efficiency distribution model

While broadening of the PRH beyond shot noise can be attributed to many underlying phenomena, the simplest way to quantify this broadening is by interpreting it as due to an underlying

distribution of FRET efficiencies. This is particularly useful for the analysis of the donor-only population, where the notion of a distance distribution is meaningless in the absence of an acceptor fluorophore. The simplest conceivable model is a normal distribution, centered on a value E_0 and characterized by a standard deviation σ_E. Since FRET efficiency values smaller than 0 or larger than 1 are meaningless, the actual distribution is truncated:

$$p(E) = \begin{cases} K \exp\left(-\dfrac{(E-E_0)^2}{2\sigma_E^2}\right), & E \in [0,\ 1] \\ 0 & \text{otherwise} \end{cases} \qquad \text{(SI.67)}$$

where K is a normalization constant ensuring that the integral of $p(E)$ over [0, 1] equals 1. With this definition, parameter σ_E is not strictly the standard deviation of the distribution (in particular if $E_0 \sim 0$) but can still be interpreted conveniently.

This model was only used to analyze the donor-only population and verify that the observed PRH was compatible with $E_0 \sim 0, \sigma_E \sim 0$. For other samples (or subpopulations), the distance model discussed in the next section was used.

11.1.4 Beta distribution of FRET efficiencies

A slightly more complex model of FRET efficiency distribution is provided by the family of beta distributions of parameters (a, b):

$$p(E) = \frac{E^{1-a}(1-E)^{1-b}}{B(a,b)}, \quad (a,b) > 0, \quad E \in [0,1]. \qquad \text{(SI.68)}$$

Parameters (a, b) can be expressed in terms of the mean E_0 and standard deviation σ_E of the distribution:

$$\begin{cases} a = \dfrac{E_0}{\sigma_E^2}\left(E_0(1-E_0) - \sigma_E^2\right) \\ b = \dfrac{1-E_0}{E_0} a \end{cases}. \qquad \text{(SI.69)}$$

It turns out that the beta distribution $p(E)$ with parameters (E_0, σ_E) differs very little from the third (geometric) model discussed in the next section.

11.1.5 Fuzzy dumbbell model of distance distribution

In ref. [24], a simple model of normal distribution of distances was used to quantify the small departure from the shot noise limited PRH. Calling $\bar{R}$ the mean distance between the two fluorophores and σ_R the standard deviation of the distribution, the PDF was defined as:

$$p(R) = \frac{1}{\sqrt{2\pi}\sigma_R} \exp\left(-\frac{(R-\bar{R})^2}{2\sigma_R^2}\right). \qquad \text{(SI.70)}$$

While this distribution is convenient, it is not based on any physical model, which should reflect the fact that two fluorophores attached to two flexible linkers are involved in the calculation

of *R*. In this respect, a slightly more natural model consists in assuming that each dye's location is characterized by an average position $\vec{r}_{i,0}$ (i = 1 or 2) and standard deviation σ_i. Noting $d = \left|\vec{r}_{2,0} - \vec{r}_{1,0}\right|$ the distance between the two average dye positions, a simple calculation yields the interdye distance PDF [34]:

$$p(R) = \sqrt{\frac{2}{\pi}} \frac{R}{\sigma d} \exp\left(-\frac{d^2 + R^2}{2\sigma^2}\right) \sinh\left(\frac{Rd}{\sigma^2}\right), \tag{SI.71}$$

where parameter σ is defined by:

$$\sigma^2 = \sigma_1^2 + \sigma_2^2. \tag{SI.72}$$

Because this model described two normally distributed objects separated by a fixed distance between their average locations, we refer to this model as the "fuzzy dumbbell" model.

This model has a few useful properties. First, it has effectively only 2 free parameters, *σ* and *d*, even though the underlying picture involves 3 parameters (d, σ_1 and σ_2). This means that very different situations could result in identical outcomes, as far as the PRH is concerned. In other words, once optimal model parameters *σ* and *d* have been obtained, there remain some flexibility (Eq. (SI.72)) to describe the system, without the need for additional model parameters. Second, contrary to the normal distribution of distance, the fuzzy dumbbell model (Eq. (SI.71)) can yield asymmetric distance distributions, which might be a desirable feature in some situations. Note that both models result in asymmetric FRET efficiency distributions for low and high FRET efficiencies, as is experimentally observed:

$$p(E) = \frac{1}{6\sqrt{2\pi}} \left(E^{-1} - 1\right)^{-2/3} E^{-2} \left\{ \exp\left[-\frac{\left(r - E^{-1} + 1\right)^2}{2s^2}\right] - \exp\left[-\frac{\left(r + E^{-1} - 1\right)^2}{2s^2}\right] \right\}, \tag{SI.73}$$

where $r = d/R_0$ and $s = \sigma/R_0$ are two reduced parameters expressed in terms of *d*, *σ* and the Förster radius R_0 (note that the Förster radius is not needed, unless the fit parameters *r* and *s* need to be converted to real units).

For all models, the search for the best fit parameter set was performed by an exhaustive grid search between min and max values for each parameters. For each set, a value *ε* from the corresponding *p(E)* PDF was used in each step (i) of the procedure described above.

Results of SNA for the single-spot measurements are represented on Fig. SI-18. Results of the 3 types of analysis (Gaussian fit, KDE and SNA) for the multispot measurements are provided in Table SI-7 to SI-11.

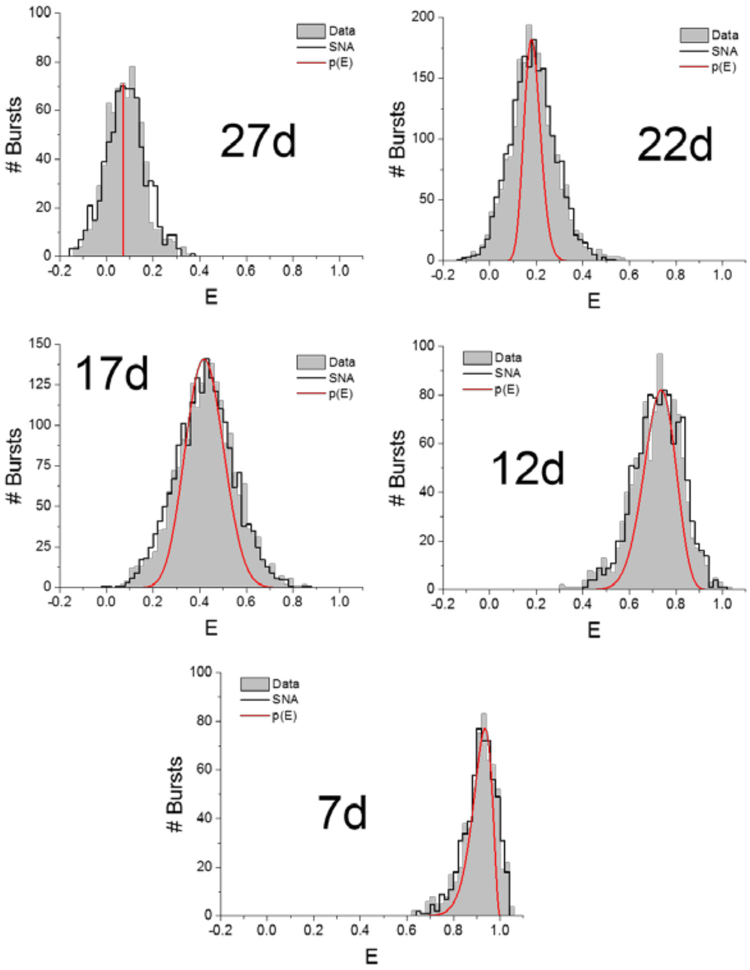

Fig. SI-18: SNA results for the μs-ALEX measurements. For each sample, the corrected FRET histogram is represented in dark gray, the fitted histogram is represented in black. Superimposed to these two histograms is the E distribution used to account for the histogram (Eq. (SI.73)). The corresponding parameters (E_0, σ_E) of the beta distribution model are:

Sample	7d	12d	17d	22d	27d
E_0	0.913	0.725	0.423	0.185	0.080
σ_E	0.045	0.068	0.080	0.035	0

The value $\sigma_E = 0$ obtained for the 27d sample simply means that there is practically very little difference between this choice of parameter and larger values (such as those obtained for other samples).

Appendix 12 FCS Analysis

This Appendix first presents details on the FCS analysis performed on both single-spot and multispot data (using ALiX), followed by results mentioned in the main text.

There are two main differences between the single-spot and multispot data as far as FCS analysis is concerned:

1. The single-spot data was obtained in the presence of laser alternation, which needs to be handled specifically in order to compare ACF and CCF curves to standard models.
2. The multispot data suffered in some cases from much larger afterpulsing, which made some of the standard ACF corrections inadequate.

This explains the presence of two distinct sections for the FCS analysis part.

12.1 Single-Spot μs-ALEX FCS

Fluorescence correlation spectroscopy (FCS) is a powerful tool to analyze molecular diffusion coefficients, brightness and stoichiometry and in some cases, short time scale dynamics [35]. In particular, the typical diffusion time through the observation volume, τ_D, can be extracted from a fit of the autocorrelation function (ACF) to the appropriate model function. The practical and quantitative implementation of this approach is usually perilous, due to a number of potential artifacts and necessary approximations [36, 37]. Since the molecules studied here are doubly-labeled, it is also possible to compute the cross-correlation function (CCF) of the signals detected in each channel corresponding to a spot.

The ACFs and CCF of the donor and acceptor channel signals upon donor excitation (photon streams $D_{ex}D_{em}$ and $D_{ex}A_{em}$) together with the ACF of the $A_{ex}A_{em}$ stream (acceptor excitation, acceptor channel detection) were calculated on a multitau time scale using the recorded arrival times using published algorithms [38]. Because of μs laser alternation, the raw ACFs, $ACF_{A-LEX}(\tau)$, exhibit a periodic modulation, which can be cancelled out by a simple renormalization by the alternation period histograms' ACFs ($ACF_{Period}(\tau)$):

$$\overline{ACF}(\tau) = \frac{ACF_{ALEX}(\tau)}{ACF_{Period}(\tau)}, \qquad \text{(SI.74)}$$

where the alternation period histograms obtained in Appendix Appendix 5 are extended over the whole duration of the experiment using their periodicity property.

As usual for SPADs, the short time scale part of the ACFs is contaminated with afterpulsing and can be corrected using the simple approach described in ref. [39], provided the time scales of interest are sufficiently well separated:

$$ACF(\tau) = \overline{ACF}(\tau) - \frac{DCR}{r}\left(ACF_{DCR}(\tau) - 1\right). \qquad \text{(SI.75)}$$

In this expression, DCR is the dark count rate of the detector (measured in a separate experiment), $ACF_{DCR}(\tau)$ its autocorrelation function and r is the actual count rate of the photon stream (computed over the period defining the photon stream under consideration, not the whole alternation period).

ACFs obtained after these corrections were fitted by a simple model of 2-dimensional diffusion through a Gaussian observation volume (beam waist: ω), with an additional blinking component (fraction λ, time scale τ_{bl}):

$$ACF(\tau) = 1 + A\left(1 + \frac{\tau}{\tau_D}\right)^{-1}\left[\frac{1 - \lambda + \lambda \exp(-\tau/\tau_{bl})}{1 - \lambda}\right]. \qquad \text{(SI.76)}$$

In this expression, the ACF amplitude A is related to:

(i) the mean occupancy N of the observation volume,
(ii) the signal-to-uncorrelated-background ratio for the photon stream under consideration,
(iii) the fraction as well as brightness of different populations,

in a complex manner requiring careful calibrations to be properly computed [40]. Since we are not interested in this information, no attempt was made to compute N.

The diffusion constant, D, common to all species in solution, is related to the beam waist parameter ω and diffusion time τ_D by the standard relation:

$$\tau_D = \frac{\omega^2}{4D}, \tag{SI.77}$$

where ω depends on the excitation and detection channel under consideration, as well as the experiment [41]. The blinking time scale τ_{bl}, generally due to single excited state to triplet state transitions, is a dye characteristic and is globally fit to all autocorrelation functions related to a given dye. To identify which stream an ACF corresponds to, we use the following indices for the fitted parameters:

- D excitation, D emission channel: DG (e.g. τ_{DG})
- D excitation, A emission channel: DR (e.g. A_{DR})
- A excitation, A emission channel: AR

Similarly to ACFs, the raw CCFs, $CCF_{ALEX}(\tau)$, exhibit a periodic modulation, which can be cancelled out by a simple renormalization by the alternation period histograms' CCFs ($CCF_{Period}(\tau)$):

$$\overline{CCF}(\tau) = \frac{CCF_{ALEX}(\tau)}{CCF_{Period}(\tau)}, \tag{SI.78}$$

where the alternation period histograms obtained in Appendix Appendix 5 are extended over the whole duration of the experiment using their periodicity property. Because afterpulses of different detectors are uncorrelated, there is no need for further corrections, and we will simply use the notation CCF for the demodulated cross-correlation function.

CCFs were fitted with a simple 2-dimensional diffusion model:

$$CCF_{GR}(\tau) = 1 + A_{GR}\left(1 + \frac{\tau}{\tau_{GR}}\right)^{-1}, \tag{SI.79}$$

where the diffusion time τ_{GR} (G: green, or donor, R: red, or acceptor) can in principle be obtained from the donor and acceptor channel ACF diffusion times, τ_{DG} and τ_{DR} by:

$$\tau_{GR} = \frac{1}{2}\left(\tau_{DG} + \tau_{DR}\right). \tag{SI.80}$$

In practice, diffusion time τ_{GR} was fitted and compared to its theoretical value, Eq. (SI.80). As for the ACF, the CCF amplitude depends in a complex manner on a variety of parameters [40], which were not computed in this study.

Fits were performed in Origin 9.1 (OriginLab Corp., Northampton, MA) using the built-in Levenberg-Marquard algorithm with statistical weights (ACF and CCF timelag range: 1 μs – 1 s).

12.2 Multispot FCS

The ACFs and CCF of the donor and acceptor channel signals upon donor excitation (photon streams $D_{ex}D_{em}$ and $D_{ex}A_{em}$) together with the ACF of the $A_{ex}A_{em}$ stream (acceptor excitation, acceptor channel detection) were calculated on a multitau time scale using the recorded arrival times as described in ref. [38].

To remove afterpulsing contamination from ACFs, the standard approach consisting in subtracting a component proportional to the ACF obtained with an uncorrelated, non-fluctuating

sample proved ineffective [39]. Instead, we used a model function comprised of a sum of 3 exponentials in addition to a 2-dimensional diffusion contribution [11], to fit each spot’s ACF, $ACF_{Y,i}(\tau)$:

$$ACF_{\mathrm{Y},i}(\tau)=\sum_{j=1}^{3}A_{\mathrm{Y},ij}\exp\left(-\frac{\tau}{\tau_{\mathrm{Y},ij}}\right)+1+\frac{1}{N_{\mathrm{Y},i}}\left(1+\frac{\tau}{\tau_{\mathrm{Y},i}}\right)^{-1}. \quad \text{(SI.81)}$$

Here i indicates which spot’s data is fitted, and Y the detection channel under consideration (Y = G or R), while $\{A_{\mathrm{Y},ij},\ \tau_{\mathrm{Y},ij}\}$ (j = 1…3) are the afterpulsing components for spot i, channel Y. Since there is no acceptor excitation laser, there is no need to specify which excitation period is considered in the notations (the excitation period is the donor excitation period).

Note that, because of the large number of exponential involved to account for afterpulsing, we did not attempt to fit a dye triplet state blinking component. In practice, the short time scale multi-exponential fitting parameters “fit out” afterpulsing as well as triplet state blinking in a global manner.

The parameters of interest are the molecular occupancy, $N_{\mathrm{Y},i}$ and the diffusion time, $\tau_{\mathrm{Y},i}$. Note that the true occupancy value would require a precise knowledge of both excitation and detection PSFs, which we do not possess [40]. Therefore, the term “occupancy” (and the corresponding parameters $N_{\mathrm{Y},i}$) should be understood as the product of a fixed concentration (common to all spots within a sample) and an “effective” volume, which might vary from spot to spot. Comparison of “occupancies” between spots will thus report on excitation/detection volume differences between spots, not on concentration.

Afterpulsing contamination is not a problem with the CCFs, since the afterpulsing of two separate channels are uncorrelated, simplifying the fit model:

$$CCF_{GR,i}(\tau)=1+\frac{1}{N_{GR,i}}\left(1+\frac{\tau}{\tau_{GR,i}}\right)^{-1}. \quad \text{(SI.82)}$$

To account for a possible lateral[9] shift d between donor and acceptor observation volumes, the expected functional form of the CCF is modified into [42]:

$$CCF_{GR,i}(\tau)=1+\frac{1}{N_{GR,i}}\left(1+\frac{\tau}{\tau_{GR,i}}\right)^{-1}\exp\left(-\frac{\left(\frac{d}{\omega_{GR,i}}\right)^{2}}{1+\frac{\tau}{\tau_{GR,i}}}\right), \quad \text{(SI.83)}$$

where $\omega_{GR,i}$ is the effective Gaussian parameter of the CCF observation volume:

$$\omega_{GR,i}^{2}=\frac{1}{2}\left(\omega_{G,i}^{2}+\omega_{R,i}^{2}\right). \quad \text{(SI.84)}$$

In the expression above, $\omega_{G,i}$ (resp. $\omega_{R,i}$) is the Gaussian parameter of the donor (resp. acceptor) observation volume, and:

[9] A similar term accounting for a potential vertical shift can be included, but, as for the diffusion component of the ACF which was not necessary in this study, the ACF is much less sensitive to this effect than to a lateral shift.

$$\tau_{GR,i} = \frac{\omega_{GR,i}^2}{4D} = \frac{1}{2}\left(\tau_{G,i} + \tau_{R,i}\right) \ . \qquad \text{(SI.85)}$$

ACF and CCF curves were generated in ALiX using a standard multitau binning scheme (8 bins per chunk, 16 bins of 12,5 ns duration for the first chunk). Fits were performed in Origin 9.1 (OriginLab Corp., Northampton, MA) using the built-in Levenberg-Marquard algorithm with statistical weights (ACF timelag range: 500 ns – 1 s, CCF: 50 µs – 1 s).

Raw occupancy parameters for ACFs were corrected for background attenuation using the standard formula:

$$\tilde{N}_{Y,i} = \frac{N_{Y,i}}{\left(1 + 1/SBR_{Y,i}\right)^2} \ , \qquad \text{(SI.86)}$$

where the signal-to-background ratio is defined as:

$$SBR_{Y,i} = \frac{r_{Y,i} - ub_{Y,i}}{ub_{Y,i}} \ . \qquad \text{(SI.87)}$$

In the formula above, $r_{Y,i}$ is the average count rate in channel *Y* for spot *i* and $ub_{Y,i}$ is the uncorrelated background rate obtained with a buffer-only sample.

Note that Eq. (SI.81) - (SI.86) do not take into account the presence of multiple species in solution (donor only, acceptor only and doubly labeled molecules, all characterized by the same diffusion coefficient), making the occupancy parameter only useful for comparison between spots within an experiment, but not between experiments.

12.3 Single-spot FCS Results

The single-spot setup detection path was designed in such a way that the size of the image PSF was smaller than the detector area (50 µm) [9]. In these conditions, the detector plays no role in defining the effective observation volume, and the emission path pinhole, common to both channels, is expected to be the only source of collection efficiency reduction. In this case, the measured diffusion times increases with wavelength: $\tau_{AR} > \tau_{DR} > \tau_{DG}$. This is confirmed by the fit parameters of individual ACFs (Fig. SI-19) reported in Table SI-12.

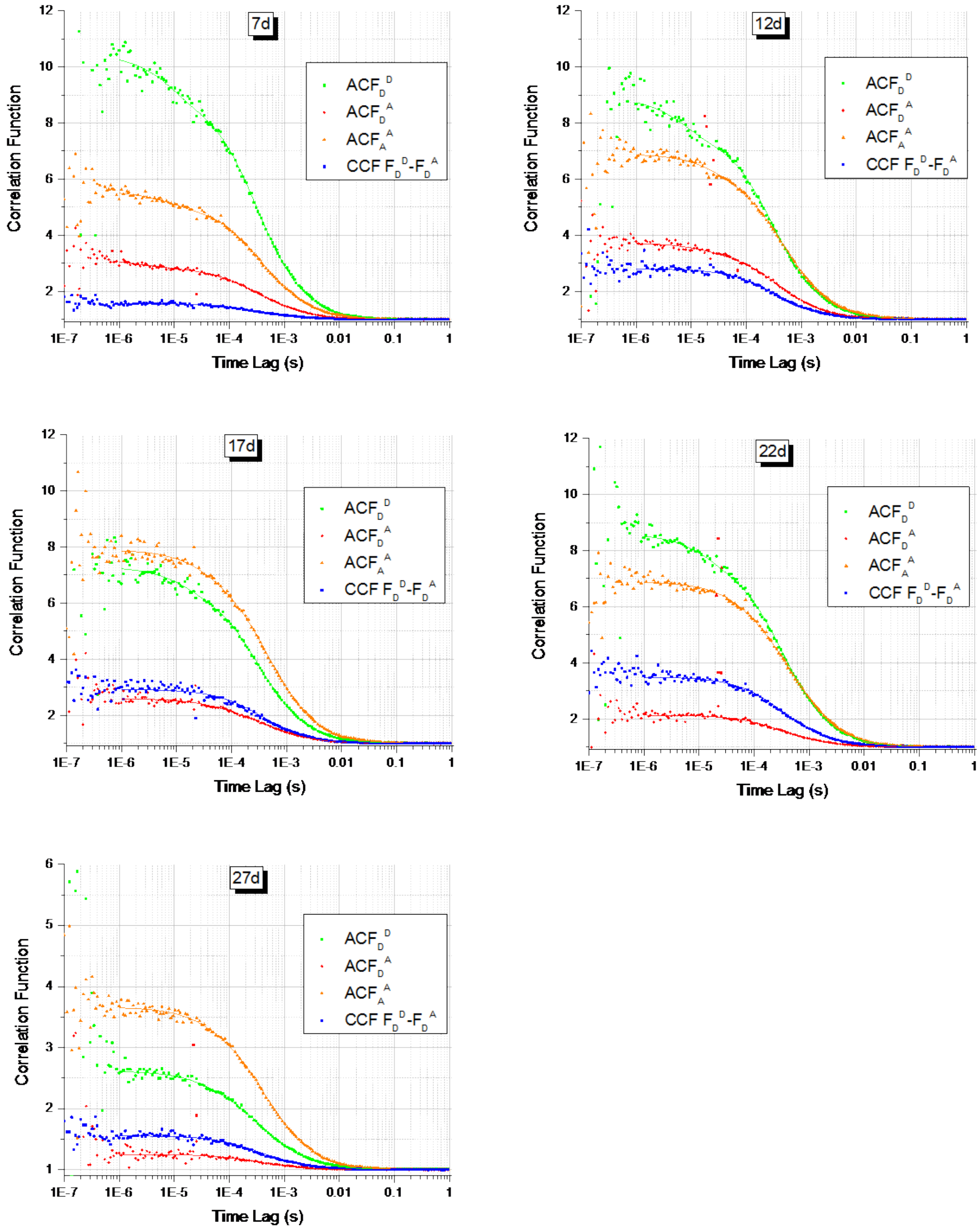

Fig. SI-19: Single spot µs-ALEX FCS analysis. ACFs and CCF of the different samples used in this study. For each sample, the ACF of the 3 streams DG, DR and AR were computed and fitted with a 2-dimensional diffusion plus triplet blinking model. The CCF of the DG and DR streams was computed and fitted with a 2-dimensional diffusion plus PSF lateral offset model (Eq. (SI.83)).

Sample	A_{DG}	τ_{DG}	A_{DR}	τ_{DR}	A_{AR}	τ_{AR}
7d	7.9	323	1.8	384	4.0	416
12d	6.6	316	2.5	382	5.3	470
17d*	5.6	306	1.5	353	6.3	447
22d	6.8	327	1.1	336	5.5	460
27d	1.5	355	0.25	358	2.6	396

Table SI-12: Amplitude A and diffusion times τ_D (in µs) (Eq.) extracted from a fit of the D-excitation, D-emission channel autocorrelation function (DG), D-excitation, A-emission channel ACF (DR), and D-excitation, A-emission channel ACF (AR). Triplet state blinking (λ ~ 10-15%, data not shown) is observed for both donor (τ_{bl} = 9 µs) and acceptor (τ_{bl} = 21 µs). (*) Only the first 400 s of dataset 17d were retained, due to the increasing background and signal level in the remainder of the trace.

Some variability in diffusion times is observed among samples, but not at a level that could lead to any suspicion of severe misalignment. However, a significant difference in all correlation amplitudes is observed for sample 27d, consistent with an increased uncorrelated background rate. Such increased background rate is most likely due to improper focus distance (lower than usual), resulting in larger scattering from the sample holder’s bottom coverslip.

12.4 Multispot FCS Analysis Results

Results are discussed in the main text. ACF and CCF curves can be found in Fig. SI-20.

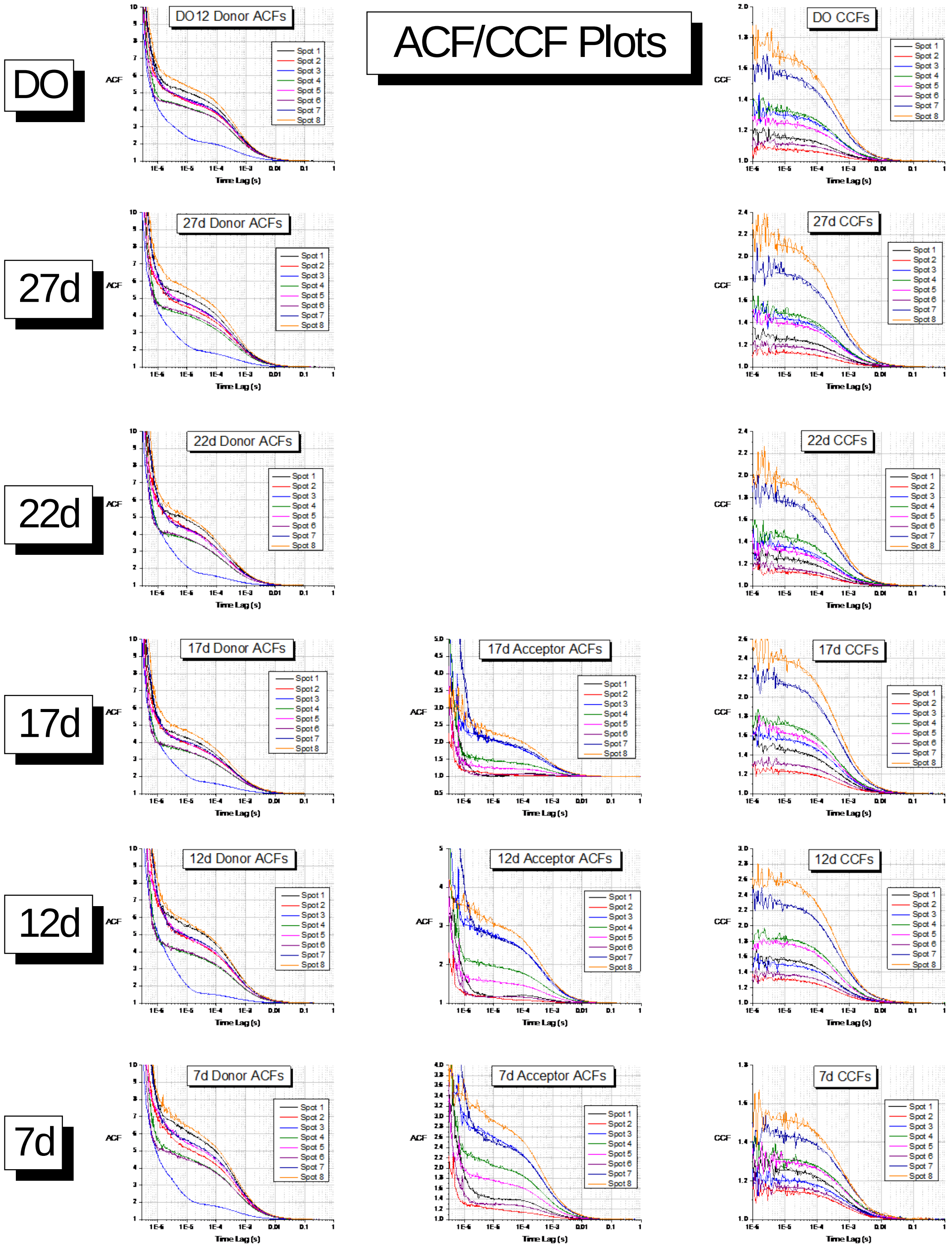

Fig. SI-20: Donor and acceptor ACF and CCF plots for multispot measurements. FCS analysis for the samples studied in this work (donor only: DO, doubly-labeled: 27d to 7d). Each row in the Figure shows data from a single sample measurement (whose name is indicated to the left). Each graph in the Figure shows the donor channel auto-correlation function (ACF, left column), acceptor ACF (center column) and donor-acceptor cross-correlation function (CCF, right column) of all 8 spots in the measurement, with their corresponding fits to the models described in the main text. The acceptor ACFs for sample DO, 27d and 22d are not shown due to the low to non-existent acceptor channel signal for these samples, which prevented any reliable fit.

Appendix 13 Samples Description

13.1 dsDNA FRET Samples

A set of 5 different FRET samples and their corresponding singly-labeled counterparts was used (Fig. SI-21). All samples consisted of a common 40 base-pair (bp) long doubly-labeled double-stranded DNA (dsDNA) with the donor (ATTO 550, ATTO-TEC GmbH, Heidelberg, Germany) on one strand and the acceptor (ATTO 647N, ATTO-TEC GmbH) on the other. The acceptor dye was bound to the 5' end of the top strand (Fig. SI-21), while the donor dye was attached to the bottom strand at different positions from the 3' end (7, 12, 17, 22 and 27 bp away from the acceptor, respectively). The sequence is identical to that used in previous work [30] and is designed in such a way that the environment of the donor dye is similar for all molecules, in order to minimize variations in donor quantum yield between samples.

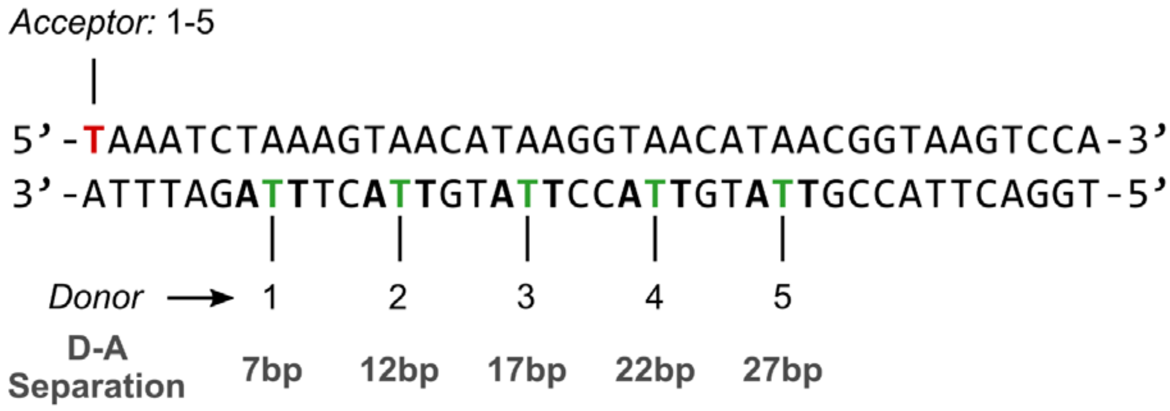

Fig. SI-21: DNA sequence used in this work, with location of the dyes indicated for each sample.

Dyes were attached to a dT residue through a C6 linker using NHS-ester chemistry. Dual HPLC purified singly-labeled ssDNA samples were purchased from IDT (Coralville, IO, USA) and used without further purification.

ssDNA molecules were hybridized to their complementary strand in a 1:1 stoichiometry to form doubly-labeled samples, and with two-fold excess of unlabeled complementary strand for singly-labeled samples.

dsDNA samples were prepared with filtered, freshly prepared TE50 buffer (Tris-EDTA 50) and kept on ice until observed. 5 µl of sample at single-molecule concentration (<100 pM) were deposited in a sealed chamber consisting of a polymer gasket sandwiched between two glass coverslips. 10 to 30 min measurements were performed at room temperature (~24 ºC).

13.2 RNAP Transcription Samples

RNA polymerase (RNAP)-promoter initiation complex (RP_O) solution was prepared as described[43]:

- 1 µl *E. coli* RNAP holoenzyme (NEB, Ipswich, MA, USA, M0551S; 1.6 µM)
- 10 µl 2X transcription buffer (80 mM HEPES KOH, 100 mM KCl, 20 mM $MgCl_2$, 2 mM dithiotreitol (DTT), 2 mM 2-mercaptoethylamine-HCl (MEA), 0.02% Tween 20, 2 mM 5 min UV-illuminated Trolox, 200 µg/ml Bovine Serum Albumin (BSA), pH 7)
- 8 µl of water

- 1 µl of 0.1 µM lacCONS promoter DNA [44] doubly-labeled with donor and acceptor dyes labeling bases in the transcription bubble in intiation: NT(-8)ATTO647N – T(-5)ATTO550 (purchased from IBA, Germany).

RP_O was then incubated in solution at 37 ºC for 30 min. To remove unreacted and nonspecifically-bound RNAP, 2 µl of 100 mg/ml Heparin-Sepharose CL-6B beads (GE Healthcare, Little Chalfont, Buckinghamshire, UK) was added to the RP_O solution together with 10 µl of pre-warmed 1X transcription buffer. The mixture was incubated for 5 min at 37 ºC and centrifuged for at least 45 s at 6,000 rpm. 20 µl of the supernatant containing RP_O wass transferred into a new tube containing 10 µl of pre-warmed 1X transcription buffer (heparin challenge[44, 45]).

The heparin challenged RP_O solution was then incubated with 1.5 µl of 10 mM Adenylyl(3′-5′) adenosine (A_pA; Ribomed, Carlsbad, CA, USA) at 37 ºC for 20 min to form a stable initially transcribed complex of up to two RNA bases ($RP_{ITC=2}$) solution. The result was a stock of $RP_{ITC=2}$ at promoter concentration of 2 nM.

smFRET experiments were performed at 100 pM promoter concentration.

Appendix 14 Single-Spot Setup Characterization

14.1 Mean Count Rates and Background Rates

Mean count rate and background rate provide a simple way to characterize a sample and its measurement. Table SI-13 reports these rates for all samples studied here (see also Fig. SI-8 for temporal variation of the total background rates).

	b_{All}	b_D^G	b_D^R	b_A^R	r_{All}	r_D^G	r_D^R	r_A^R
7d	2,773	489	1,156	871	3,713	773	1,347	1,219
12d	2,255	444	962	654	3,191	676	1,192	1,002
17d*	2,149	554	843	588	3,138	869	980	966
22d	2,612	636	889	809	4,095	1,176	1,039	1,457
27d	3,986	1,523	996	1,102	4,905	1,983	1,061	1,367

Table SI-13: Background (b_X^Y) and mean (r_X^Y) count rates (in Hz) recorded for different photon streams XY (X: excitation period, Y: emission channel) for the 5 samples. All: All counts; D (resp. A): donor (resp. acceptor) excitation; G (resp. R): donor/green (resp. acceptor/red) channel detection. (*) For sample 17d, only the first 400 s of the measurement were used, due to the subsequent increase discussed in Fig. SI-8.

From this data, sample 27d appears to have a significantly larger background than the other samples (b_{All} column), the increase being particularly noticeable in the donor-excitation donor-detection stream (b_D^G column). Sample 22d on the other hand, comes as a close second in terms of total mean count rate (r_{All} column), while its background rate is not significantly different than most other samples. This suggest that these two samples exhibit these larger rates for distinct reasons.

When all samples are characterized by the same molecular brightness (which is a good approximation in these samples, as will be discussed later), both mean and background count rates will increase proportionally to sample concentration or excitation intensity. To distinguish which of these two effects is responsible for the observed differences, an observable which does not depend on concentration is needed.

14.2 Peak Burst Count Rate

The maximum peak burst count rate would seem to provide such an information. Indeed, a larger excitation intensity will result in a proportionally larger peak burst count rate, as long as saturation is negligible. Increase in the observation volume without any change in the peak excitation intensity should not affect it either. However, it is an elusive quantity to measure. In particular, it is sensitive to the presence of rare multiple molecule events, which can pass as very large single-molecule bursts.

As discussed in the Appendix 14.3, the mean value of the peak burst count rate $< r_{max} >$ for bursts above a threshold value $r_{max,0}$, is a good alternative observable, in the sense that it does not, to a large extent, depend on the burst search and selection parameters, and scales proportionally to the peak excitation intensity. In order to obtain a similar value for all samples, the peak total count rate during donor excitation, $r_{D,max}$, was chosen. Table SI-14 shows the values of $< r_{D,max} >$, $r_{D,max} > 300$ kHz, for all 5 samples and for two different types of burst search (APBS, $m = 5$, $F = 6.8$ and APBS, $m = 5$, $r_{min} = 27$ kHz). The first search uses a common minimum SBR criterion (resulting effectively in different searches for samples characterized by different background rates), while the second uses a common burst count rate threshold criterion, equal to the threshold used for the noisiest sample in the first search.

	7d	12d	17d*	22d	27d
$F = 6.8$	391	405	418	411	418
$r_{min} = 27$ kHz	393	406	417	409	419

Table SI-14: Average peak burst count rate during donor excitation (in kHz), $r_{D,max}$, computed for bursts with $r_{D,max} >$ 300 kHz, in two different burst searches: 1) $F = 6.8$, constant SBR burst search, using $m = 5$; 2) $r_{min} = 27$ kHz, constant burst count rate threshold, $m = 5$. (*) Only the first 400 s of data set 17d were retained, due to the increased background rate in the rest of the trace.

We conclude that the peak burst count rate during donor excitation is similar for all samples. This suggests that, as originally intended, there are no significant difference in excitation intensity and detection efficiency between measurements, and that the reason for the variability in background count rate and mean count rate needs to be attributed to other causes. This leads to the investigation of another measurement observable, the detected burst rate (number of bursts per unit time).

14.3 Burst Rate *n(t)*

The number of detected bursts per unit time (burst rate) depends on many parameters, including burst search and burst selection parameters. However, if sample and measurement characteristics are similar, or burst search and selection parameters are chosen in such a way as to compensate for differences, we can expect to be able to reliably compare measurements using this observable.

Table SI-15 reports the number of bursts per second detected in each measurements, using two different burst search and selection parameters.

	7d	12d	17d*	22d	27d
$F = 6.8$	2.4 ± 2.0	2.3 ± 1.9	2.5 ± 2.0	4.1 ± 2.6	1.3 ± 1.4
$r_{min} = 27$ kHz	1.1 ± 1.2	0.9 ± 1.1	1.0 ± 1.2	2.1 ± 1.8	1.0 ± 1.2

Table SI-15: Number of bursts detected per second (± standard deviation) computed for bursts with total γ-corrected size $F_{SBC} \geq 30$ in two different burst searches: (i) $F = 6.8$, minimum SBR burst search, using $m = 5$; (ii) $r_{min} = 27$ kHz,

constant burst count rate threshold, $m = 5$. (*) Only the first 400 s of data set 17d were retained, due to the increased background rate in the rest of the time trace.

The results for sample 27d are almost identical in both cases, as expected, since both burst searches are essentially equivalent for that sample (27d is the noisiest sample of the series). For all other samples, once the effect of background rates is properly compensated (*i.e.* when a constant count rate threshold is used, as used for the second row of Table SI-15), it appears that approximately twice as much bursts are detected in sample 22d than in the other samples. From this information, combined with the peak burst count rate identity among samples, we can infer that the increased mean count rate for sample 22d is likely due to a larger concentration in that sample.

What could have caused the simultaneous increases of background rate and mean count rate in sample 27d? To address this question, we need to resort to yet another independent observable obtained by fluorescence correlation analysis.

14.4 FCS analysis

The single-spot FCS analysis results were discussed in Appendix 11.4. There, we concluded that the 27d sample was characterized by an increased uncorrelated background rate, most likely due to laser scatter off the sample's bottom coverslip.

Appendix 15 Burst Statistics Definitions

While bursts are defined by their start and end photons, and can be characterized by a few quantities such as raw photon counts F_D^G, F_D^R, F_A^R, etc., as well as burst duration ΔT, it is possible to compute various other characteristics using the arrival time and detection channel of each photon comprising the burst. Quantities such as the proximity ratio (*PR*) and stoichiometry ratio (*SR*) or their corrected equivalents (E_{PR}, S or E, S_γ) which have been introduced in Appendix 8 & Appendix 9, are used to identify and characterize FRET populations.

Other quantities are useful to characterize population concentrations, diffusivity, brightness or setup characteristics, such as relative excitation/detection efficiencies. The following quantities will be used in the remainder of this work.

15.1 Peak Burst Count Rate

The peak burst count rate in stream XY, $r_{X,\max}^{Y}$, where X represents the excitation period (D or A) and Y the detection channel (G or R), is defined by:

$$r_{X,\max}^{Y} = \max\left(r_m\left(t_i\right), t_i \in \text{burst}\right), \qquad \text{(SI.88)}$$

where $r_m(t_i)$ is defined by Eq. (SI.25) with $c = 1$. In this work, a value of $m = 10$ was used to define the local count rate. If Y is omitted, the peak burst count rate during excitation period X is computed, including counts from both R and G channels.

Note that Eq. (SI.25) for the count rate needs to be modified in the presence of µs-ALEX alternation, as discussed in the next section.

15.2 Count Rate in the Presence of Laser Alternation

The previous count rate formula (Eq. (SI.25)) does not take into account which excitation period the timestamps belong to. In other words, this formula is only valid in the absence of alternation. In the presence of alternation, computing a count rate based on this formula yields problematic results if the analysis is limited to a specific excitation period, as would be the case if we wanted to compute the count rate in stream DG, for instance. This situation is illustrated in Fig. SI-22A.

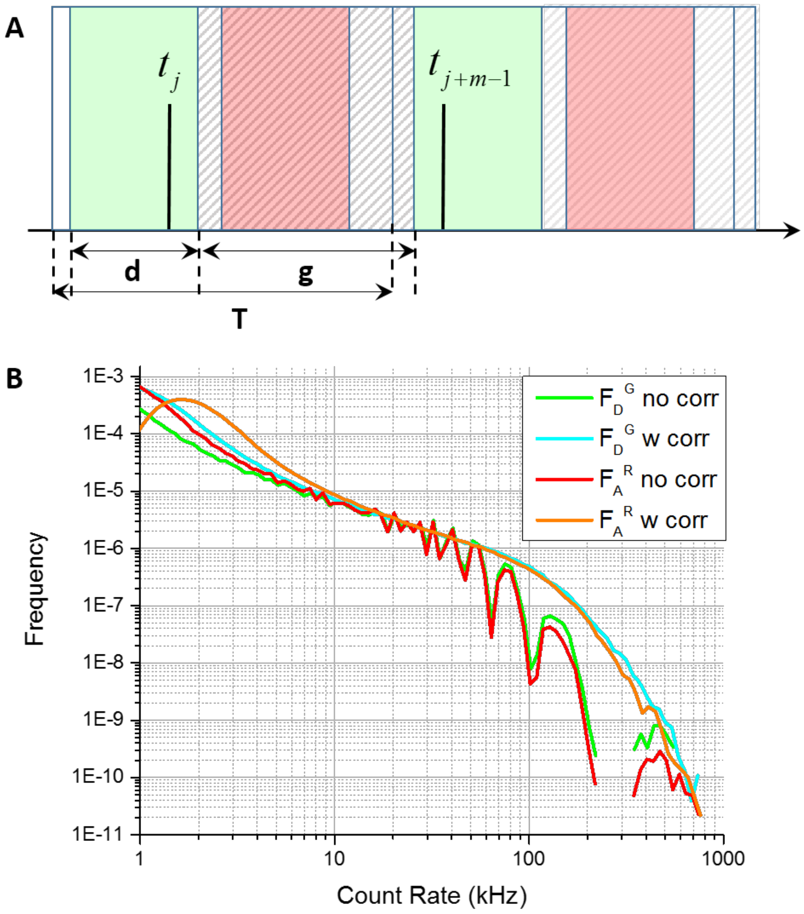

Fig. SI-22: **µs-ALEX corrections to the count rate.** (**A**) Illustration of the gap between successive D-excitation periods (green rectangles). The total duration of the alternation period, *T*, can be decomposed into that of the D-excitation period, *d*, and its separation (or gap), *g*, from the next D-excitation period. What happens during this gap is irrelevant to compute the count rate relative to D-excitation period photons, and needs to be subtracted from the final inter-photon delay, as described in Appendix 14.1. (**B**) Illustration of the effect of using the uncorrected formula (Eq. (SI.88), green and red curves) or the µs-ALEX-corrected formula taking into account emission gaps (Eq. (SI.92), blue and orange curves). Data from file 7d. *m* = 10 was used. Notice the dips corresponding to a gap of approximately 25 µs between consecutive D-excitation (resp. A-excitation) window in the uncorrected DG (resp. AR) count rate curve (green (resp. red)).

To simplify the discussion, we only represented the first and last photons of a particular bunch of *m* consecutive photons in the DG stream, with respective timestamps t_j and t_{j+m-1}. From the figure, it is apparent that the minimum separation between both timestamps is equal to the gap between two successive D-excitation periods, *g*. Similarly, if the two timestamps were two alternation periods apart, the minimum separation would be $2g + d = g + T$, where *d* is the D-excitation window duration, and $d + g = T$, the alternation period duration. More generally, if the

two timestamps were p alternation periods apart, their minimum separation would be $g + (p - 1) T$, creating artificial dips in the count rate distribution, as illustrated in Fig. SI-22B.

From Fig. SI-22A, it is clear that to ignore the gaps during which no DG photons is detected, the following *apparent* photon separation should be used:

$$\Delta t_j^{(m)\prime} = \Delta t_j^{(m)} - (p-1)g\,. \tag{SI.89}$$

Calling the D-excitation period offset t_0, it is easy to show that the number p of D-excitation periods separating the two timestamps is:

$$p = E\left(\frac{t_{j+m-1} - t_0}{T}\right) - E\left(\frac{t_j - t_0}{T}\right), \tag{SI.90}$$

where $E(x)$ designate the integer part of x. The final µs-ALEX-corrected formula for the count rate is thus:

$$r_m'(t_j) = \frac{m-2}{\Delta t_j^{(m)\prime}}, \tag{SI.91}$$

where $\Delta t_j^{(m)\prime}$ is defined by Eq. (SI.89).

The µs-ALEX-corrected formula for the peak count rate is therefore modified into:

$$r_{X,\max}'^{Y} = \max\left(r_m'(t_i), t_i \in \text{burst}\right). \tag{SI.92}$$

Note that in the previous derivation, the fact that donor photons were considered did not play any role, therefore the previous formula applies irrespective of which emission channel is considered. If A-excitation period photons had been considered instead, the same formulas would apply, with the simple replacement of g by the gap between successive A-excitation periods and t_0 by the A-excitation period offset. The result of these substitutions is illustrated in Fig. SI-22B, which shows continuous count rate distributions after correction, and a concomitant increase in the average count rate, as expected from the removal of gaps.

15.3 Proxy for the Maximum Peak Count Rate

In this section, we discuss how a *typical* peak burst count rate of a sample can be defined in a way which does not depend on the number of bursts or on the sample concentration and reflects the excitation intensity used during a measurement.

Because of the finite number of detected bursts and the stochastic nature of diffusion, the maximum peak count rate observed in the sample is not a reliable measure. Moreover, the maximum will most likely correspond to a multiple-molecule burst, and therefore artificially bias the result when using concentrated samples.

Computing the average peak count rate of *all bursts* is not a good option either, because the low peak count rate bursts (gray boxed region in Fig. SI-23A) are usually those which barely made it during the burst search or burst selection steps of the analysis, and are therefore very sensitive to search and selection parameters, including background rates.

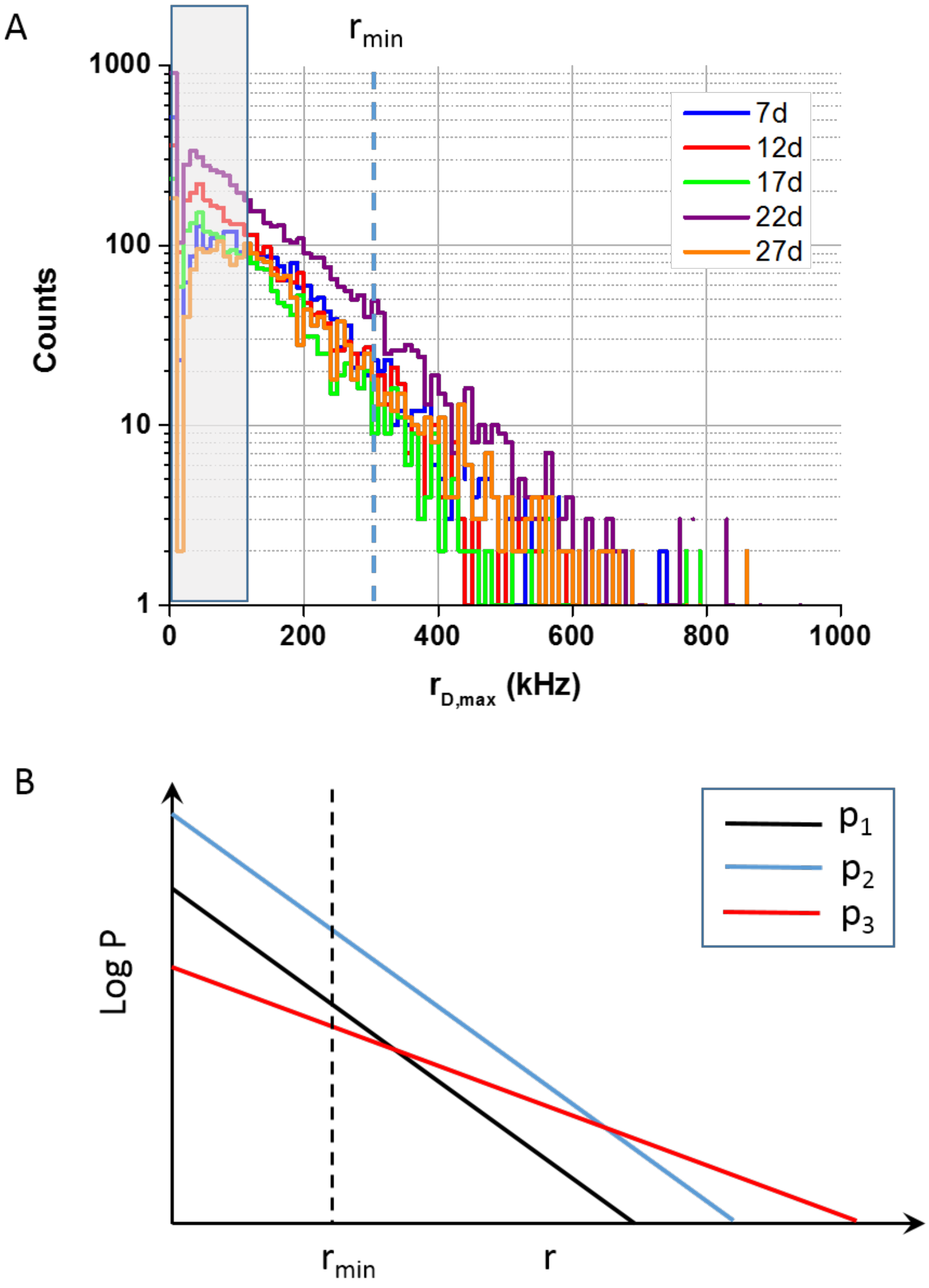

Fig. SI-23: Single-spot µs-ALEX peak burst count rate histograms. Histograms of peak burst count rate (total signal during donor-excitation), whose shape should be independent of which species is observed, due to the similar brightness of all species, provided the excitation intensity and detection efficiencies are similar in all measurements. r_{min} = 300 kHz is the minimum value used to compute the mean peak burst count rate discussed in the text. Notice that while the 22d sample measurement collected more bursts (due to a higher concentration), its average peak burst count rate was similar to that of the other samples.

To stay away from this region, a peak count rate threshold r_{min} needs to be chosen, which is neither too close to the minimum peak count rate, nor too close to the maximum peak count rate, in order to have enough statistics to compute a reliable average.

We will now show that this bounded, mean peak count rate has the required properties.

Let’s define $p_1(r)$ as the peak rate histogram in a given measurement (we do not specify which stream is considered, because this is irrelevant, as long as the same choice is kept for all other measurements, as assumed here). Another measurement of the same sample resulting in more (resp. less) bursts will have a peak rate histogram $p_2(r) = A\, p_1(r)$, where $A > 1$ (resp. < 1), while a measurement of the same sample performed with a different excitation intensity (or detection efficiency), will be characterized by a peak rate histogram $p_3(r) = B\, p_1(\theta r)$ (Fig. SI-23B).

The mean peak rate computed above r_{min} is, for each case i = 1, 2, 3:

$$\begin{aligned} \langle r \rangle_i &= I_i / N_i \\ N_i &= \int_{r_{\min}}^{\infty} dr\ \ p_i(r) \\ I_i &= \int_{r_{\min}}^{\infty} dr\ \ r\, p_i(r) \end{aligned} \qquad \text{(SI.93)}$$

From these definitions, it follows immediately that $\langle r \rangle_1 = \langle r \rangle_2$, no matter what functional form $p_i(r)$ has. This demonstrates that, as long as r_{min} is chosen in such a way that N_1 and N_2 are not too small (large enough sampling), samples characterized by the same excitation/detection properties will yield a comparable mean peak rate $\langle r \rangle$.

On the other hand, for the measurement characterized by either a different excitation intensity or detection efficiency, we have:

$$\langle r \rangle_3 = \frac{1}{\theta} \frac{\int_{\theta r_{\min}}^{\infty} dr\, r\, p_3(r)}{\int_{\theta r_{\min}}^{\infty} dr\, p_3(r)} \ . \qquad \text{(SI.94)}$$

To compare this quantity to the other two, we need to assume a particular functional form. As shown in Fig. SI-23A, peak count rates distribution tails are generally well approximated by an exponential distribution:

$$p_1(r) \simeq K \exp\left(-\frac{r}{r_0}\right), \quad r \geq r_{\min} \ . \qquad \text{(SI.95)}$$

Using this approximation, we obtain:

$$\begin{aligned} \langle r \rangle_1 &= \langle r \rangle_2 \simeq r_0 + r_{\min} \\ \langle r \rangle_3 &\simeq \frac{r_0}{\theta} + r_{\min} \end{aligned} \qquad \text{(SI.96)}$$

where r_0/θ replaces r_0 in the exponential argument of Eq. (SI.95) for $p_3(r)$. For $\theta < 1$ (larger excitation rate, or detection efficiency), the bounded, mean peak count rate $\langle r \rangle_3$ is larger than that computed for the other two cases, and the ratio of excitation intensity times detection efficiency can be obtained as:

$$\theta = \frac{\langle r \rangle_1 - r_{\min}}{\langle r \rangle_3 - r_{\min}} \ . \qquad \text{(SI.97)}$$

15.4 γ-Corrected Burst Size

Optimal burst selection criteria depend on the analysis purpose. If the goal of the analysis is to quantify the relative molecular concentrations of different species, using a uniform size selection criterion will in general provide a biased estimate of the relative concentrations. To understand this, we consider the experiments performed in this study, where identical molecules differing only in the number and location of dyes diffuse in an identical manner through the same excitation spot. The case of multiple spots with different characteristics is discussed later in the appendix.

In order to compare the expected burst sizes of different species, we need to go back to their theoretical expressions [30].

For *D-only molecules*, the signal (or burst size) depends on the absorption cross-section σ_D^D of donor (species D) at the D-excitation wavelength, the mean excitation intensity I_D over the molecule's trajectory through the excitation volume, the fluorescence quantum yield ϕ_D of the molecule and the detection efficiency η_D^G of the setup for species D in the donor channel (Eq. (5) of Lee *et al.* [30] with $E = 0$):

$$\tilde{F}_D^G = \sigma_D^D \, I_D \, \phi_D \, \eta_D^G \ . \qquad \text{(SI.98)}$$

For *doubly-labeled species* (e.g. the FRET sub-population), 3 photon streams are available in µs-ALEX experiments: $\tilde{F}_D^G$, $\tilde{F}_D^R$ and $\tilde{F}_A^R$ and 2 in single laser excitation experiments: $\tilde{F}_D^G$ and $\tilde{F}_D^R$.

We will first discuss the latter case and come back to the µs-ALEX situation later on.

It is easy to verify from the theoretical expressions of $\tilde{F}_D^G$, $\tilde{F}_D^R$ and γ [30]:

$$\begin{aligned} \tilde{F}_D^G &= \sigma_D^D \, I_D \, \phi_D \left(1 - E\right) \eta_D^G \\ \tilde{F}_D^R &= \sigma_D^D \, I_D \, \phi_A \, E \, \eta_A^R \\ \gamma &= \frac{\phi_A \, \eta_A^R}{\phi_D \, \eta_D^G} \end{aligned} \qquad \text{(SI.99)}$$

that the quantity $\tilde{F}_D^G + \gamma^{-1} \tilde{F}_D^R$ is identical to that obtained with a D-only molecule (Eq. (SI.98)) following the same trajectory. We call the quantity:

$$\tilde{F}_\gamma = \tilde{F}_D^G + \gamma^{-1} \tilde{F}_D^R \qquad \text{(SI.100)}$$

the γ-corrected burst size.

Note that this definition arbitrarily uses D-only as a reference, and a perfectly valid alternative definition of a corrected burst size could be chosen such that it equals that of an A-only molecule following the same trajectory:

$$\tilde{F}'_\gamma = \gamma \tilde{F}_D^G + \tilde{F}_D^R = \gamma \tilde{F}_\gamma \ . \qquad \text{(SI.101)}$$

In µs-ALEX experiments, a 3rd photon stream is available ($\tilde{F}_A^R$) and the uncorrected total burst size, $\tilde{F}_D^G + \tilde{F}_D^R + \tilde{F}_A^R$, will in general depend on which species follows a given trajectory. From the definition of $\tilde{F}_A^R$:

$$\tilde{F}_A^R = \sigma_A^A I_A \phi_A \eta_A^R \ , \tag{SI.102}$$

we obtain:

$$\frac{\tilde{F}_A^R}{\tilde{F}_D^G} = \frac{\sigma_A^A I_A}{\sigma_D^D I_D}\frac{\phi_A \eta_A^R}{\phi_D \eta_D^G} = \beta\gamma \ , \tag{SI.103}$$

where β characterizing the *cross-section and excitation intensity ratio* between the two species (excitation properties, Eq. (14) in [30]):

$$\beta = \frac{\sigma_A^A I_A}{\sigma_D^D I_D} . \tag{SI.104}$$

The relation between β and the γ-corrected stoichiometry ratio S_γ (Eq. (13) derived in Lee *et al.*)[30]:

$$\beta = S_\gamma^{-1} - 1, \tag{SI.105}$$

shows that, when excitation intensities are chosen such that S_γ = ½ for a doubly-labeled species, β = 1 and Eq. (SI.103) reduces to:

$$\frac{\tilde{F}_A^R}{\tilde{F}_D^G} = \gamma \ . \tag{SI.106}$$

Equation (SI.103) simply states that, for the same hypothetical trajectory, the signal detected from an A-only (AO) molecule will be equal to $\beta\gamma$ times that of a D-only (DO) molecule.

If $\beta\gamma$ is different from 1, setting an identical signal threshold in an APBS for both species will reject more bursts of one species than the other. If the purpose of the analysis is to quantify the respective amount of D-only and A-only species, it is therefore necessary to perform a selection in which the size criterion for A-only species is $\beta\gamma$ times that for the D-only species.
To select all bursts with a single threshold (DO, AO and doubly-labeled species - DA), several definitions of a “corrected burst size” are possible. All involve first determining whether a burst is due to a DO, AO or DA molecule. Two examples are indicated in Table SI-16.

In Method 1, a different formula is used for different species, while in Method 2, the same formula is used to compute a “stoichiometry- and γ-corrected” burst size $\tilde{F}_{SBC}$:

$$\begin{aligned}\tilde{F}_{SBC} &= \frac{1}{n}\left(\tilde{F}_\gamma + (\gamma\beta)^{-1}\tilde{F}_A^R\right) \\ &= \frac{1}{n}\left(\tilde{F}_D^G + \gamma^{-1}\tilde{F}_D^R + (\gamma\beta)^{-1}\tilde{F}_A^R\right)\end{aligned} \tag{SI.107}$$

where n is the number of fluorophores in the molecule. Method 1 corresponds to the definition introduced for non-ALEX measurements (Eq. (SI.100)), for which β is undefined, and does not involve the AR stream for doubly-labeled species. Method 2 involves the AR stream, and is appropriate for studies in which it is critical to compare the relative amount of all 3 populations.

A simple way to determine which value of n to use for a particular burst consists in computing its stoichiometry ratio, SR. If $SR_{min} \leq SR \leq SR_{max}$, where $SR_{min} = 0.2$ and $SR_{max} = 0.85$, then $n = 2$, otherwise, $n = 1$.

Method	D-only	A-only	D & A
1	$\tilde{F}_D^G$	$(\gamma\beta)^{-1}\tilde{F}_A^R$	$\tilde{F}_D^G + \gamma^{-1}\tilde{F}_D^R$
2	$\tilde{F}_{SBC}, n=1 \left(=\tilde{F}_D^G\right)$	$\tilde{F}_{SBC}, n=1 \left(=(\gamma\beta)^{-1}\tilde{F}_A^R\right)$	$\tilde{F}_{SBC}, n=2$

Table SI-16: Two possible ways to compute a stoichiometry- and γ-corrected burst size. In Method 1, a different formula is used for each species, while in Method 2, the same formula is used, with an additional parameter n specifying the number of fluorophores in the molecule. For Method 2, the equivalent theoretical expression for $\tilde{F}_{SBC}$ is indicated.

15.5 SBR & SNR

- *Signal-to-background ratio (SBR)* during excitation period X, defined as:

$$SBR_X = \frac{\bar{F}_X^G + \bar{F}_X^R}{\left(b_X^G + b_X^R\right)\Delta t} \quad . \tag{SI.108}$$

- *Signal-to-noise ratio (SNR)* during excitation period X, defined as:

$$SNR_X = \frac{\bar{F}_X^G + \bar{F}_X^R}{\sqrt{F_X^G + F_X^R}} \quad . \tag{SI.109}$$

One of the main difference between the single-spot and multispot measurements is the observed background levels, due in part to the larger observation volumes of the multispot experiment, but most importantly, to the larger intrinsic (dark count) noise of some the SPADs in the arrays used in this study. While background correction takes this effect into account when computing burst statistics (such as the proximity ratio or the FRET efficiency), it is important to remember that background affects burst search results in many ways.

Here, we explore this question (also discussed in Appendix 13.2), by looking at the burst signal-to-background ratio (SBR, Eq. (SI.108)), and compare it to a related quantity, the burst signal-to-noise ratio (SNR, Eq. (SI.109)). We will focus on the donor-excitation version of these definition, since it is the only quantity common to both types of measurements.

SBR plays an important and obvious role in a minimum SBR search, where the burst count rate threshold is defined as a multiple (F) of the local background count rate. As discussed in Appendix 7.2.1, F - 1 is the minimum burst SBR, independent of the local background rate (Eq. (SI.26)). However, as illustrated in Fig. SI-24A, for a given measurement, a 15 kHz change in the burst count rate threshold can more than double (or halve) the final number of bursts after size selection. Assuming that the single-molecule signal itself is unchanged in both situations, a significant reduction of the duration of the bursts detected with higher threshold is expected, with a concomitant reduction in total burst size. This is the reason why a constant burst count rate threshold was used instead, when comparing burst duration among spots in Section 4.2.3 of the

main text, since a search using a fixed burst count rate threshold, by definition, eliminates differences in threshold between experiments characterized by very different background rates.

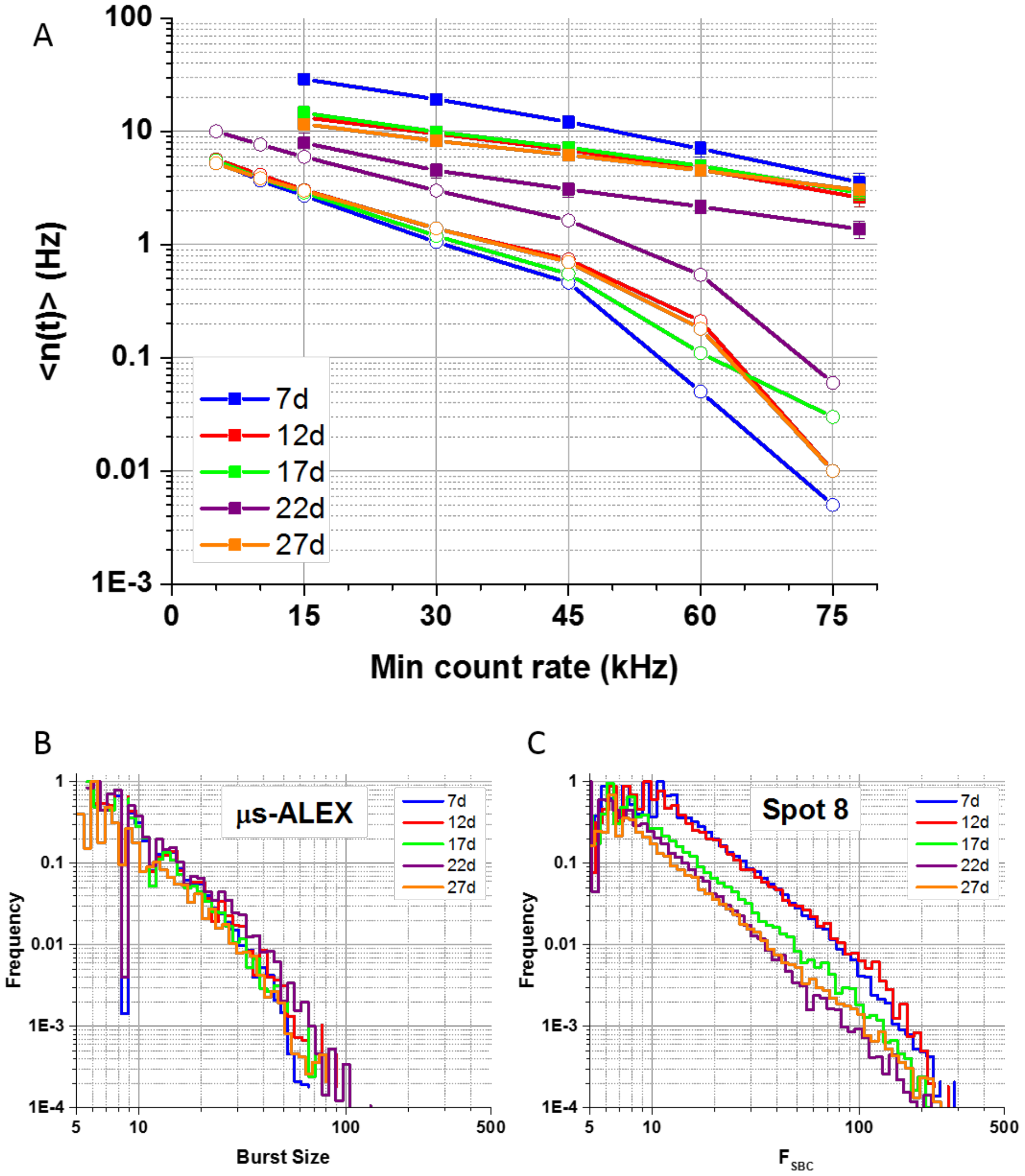

Fig SI-24. (A) Average burst rate measured for the 5 dsDNA samples, as a function of burst search count rate threshold r_{min}. A constant $F_{SBC} \geq 30$ selection criterion was used throughout (using $\gamma = 1$ for the single-spot μs-ALEX measurements and $\gamma = 0.4$ for the multispot measurements). The multispot points represent the average rate of all spots, while the error bar represents the standard deviation over all spots. Multispot analysis was limited to $r_{min} \geq 15$ kHz due to a maximum observed background rate of 11.5 kHz. Single-spot μs-ALEX analysis was limited to $r_{min} \geq 5$ kHz due to a maximum observed background rate of 4.9 kHz (Table SI-13). (B, C) Normalized F_{SBC} (γ-corrected burst size) distributions observed with a donor-excitation burst search ($m = 5$, $r_{min} = 30$ kHz) in the single-spot μs-ALEX measurements

(B) and one of the spots (spot 8) in the multispot measurements (C). Similar distributions were observed for the others spots.

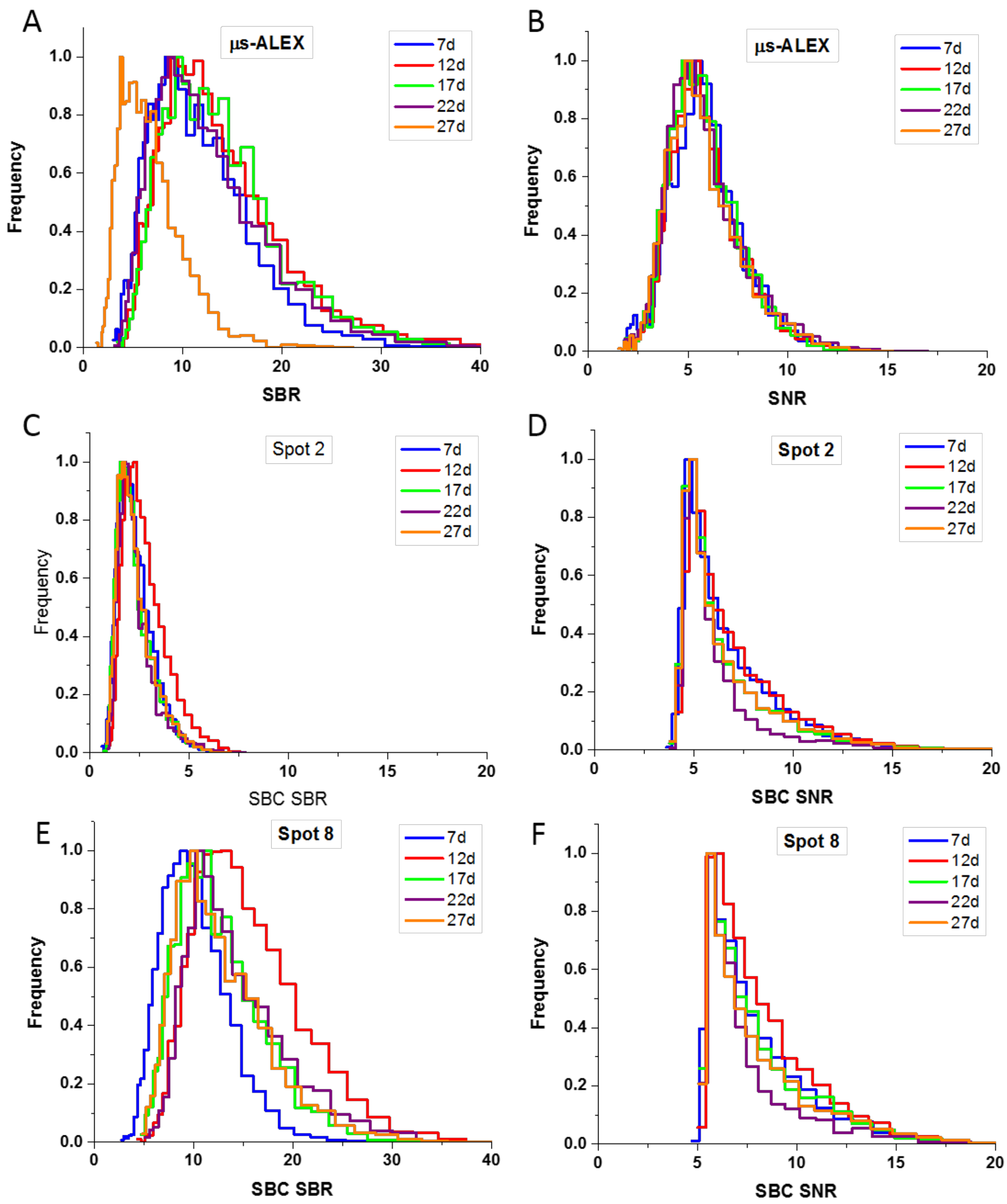

Fig SI-25. Signal-to-background-ratio (SBR) and signal-to-noise-ratio (SNR) distributions for single-spot µs-ALEX (A, B) and multispot (C-F) measurements. The single-spot µs-ALEX analysis was performed using a fixed burst search count rate threshold of 5 kHz, while the multispot analysis was performed using a fixed burst search count rate threshold of 15 kHz. 5 kHz is close to the background rate observed in sample 27d (Table SI-13), which results in a lower SBR than the for other samples (A), but similar SNR (B). Similarly, 15 kHz is close to the background observed in Spot 2, which results in a low SBR for all samples (C), while the SNR is relatively independent on the sample (D). By comparison, spot 8, which has a background rate close to 7 kHz lower than Spot 2 (Table 1, main text), exhibits

much larger SBR (E), but comparable SNR (F). The single-spot µs-ALEX signal used in this study is the donor-excitation signal. For the multispot calculation, the γ-corrected signal $\tilde{F}_{SBC}$ and γ-corrected background was used.

However, using a search with fixed burst count rate threshold does not guarantee a minimum value for the burst SBR anymore. This is illustrated in Fig. SI-25A (and SI-25B), where the SBR (and SNR) distributions are shown for the different samples studied with the single-spot µs-ALEX setup, using a common count rate threshold of 5 kHz (corresponding to the largest background rate among all samples, or leftmost point in Fig. SI-24A). The sample with the largest background rate (27d) is now characterized by the smallest SBR, while the signal itself (and hence the SNR) is almost identical for all samples.

A comparable conclusion can be drawn from the comparison of the noisiest (spot 2) and quietest spot (spot 8) of the multispot experiments. Using a common count rate threshold of 15 kHz (corresponding to the largest background rate among all spots and measurements, or leftmost point in Fig. SI-24A), the observed SBR in spot 2 (Fig. SI-25C) is systematically and markedly lower than that observed in spot 8 (Fig. SI-25E), while both exhibit similar SNR (Fig. SI-25D & SI-25F).

In summary, a constant SBR search, while convenient to compare samples characterized by similar background levels, can result in biased statistics in situations where background levels are quite different. The use of a constant rate threshold appears preferable in most situations, the optimal value depending on the peak count rate of the sample, the desired number of bursts and minimal SNR.

15.6 Burst rate *n(t)*

The number of single-molecule bursts detected per unit time during a measurement is a simple observable allowing to compare measurements or setups, and specifically to demonstrate the increased throughput of multispot data acquisition. However, its definition depends on parameters used for burst search and burst selection (*e.g.* Appendix 13.3), as well as sample and setup characteristics, such as concentration, excitation power, background rate or detection efficiency. For instance, comparing the number of bursts larger than a certain size in two different measurements characterized by different observation volumes (but otherwise similar characteristics), will obviously result in a larger number of bursts for the experiment characterized by the largest observation volume. As discussed previously, the single-spot µs-ALEX measurements and the multispot measurements were characterized by similar peak excitation power, but larger observation volumes and lower acceptor detection efficiency in the multispot experiments. Since the absolute concentration of the different samples was not determined, the only comparison which can be performed is between burst rates computed with similar burst selection criteria (γ-corrected burst size $\tilde{F}_{SBC} \geq 30$), and for the multispot experiments, using searches with identical burst thresholds across spots. The results obtained for various burst search count rate thresholds r_{min} are plotted in Fig. SI-24A, together with the corresponding rates measured in the single-spot µs-ALEX experiments (open symbols). The measured burst rates are significantly lower in the single-spot µs-ALEX experiment (up to one order of magnitude in some cases), in part because of the overall larger bursts observed in the multispot experiments (Fig. SI-24B & C), but also possibly due to concentration differences.

Fig. SI-24A also demonstrates the exponential dependence of the detected number of bursts on the burst search count rate threshold r_{min}. While this suggests a simple way to increase the

number of detected bursts (by reducing the count rate threshold), decreasing the count rate threshold has negative consequences on other metrics, as was discussed in the previous section.

Appendix 16 Simple model of the DNA double-helix with two labels

16.1 Model description

To compare the FRET efficiencies measured for each sample in the µs-ALEX experiments to those measured in the multispot experiments, no model is necessary. However, since the dsDNA samples used in this work are simple, and a similar comparison was performed in previous studies, the corrected FRET efficiencies were compared to a simple model of double stranded helix, with fluorophores attached to base pairs located at different positions along its sequence.

The model used in this work is slightly different from the popular "Clegg model"[46] and offers some additional degrees of freedom, as illustrated in Fig. SI-26 & SI-27. The double helix is modeled by the two helices passing through the phosphorus atoms (P) of each nucleic acid base (helix 1: green, helix 2: red), supported by a 1 nm radius cylinder. The angle $\widehat{H_1 \Omega H_2} = \Delta\varphi$ between the two helices and the cylinder axis (Fig. SI-26A) in any plane perpendicular to the axis is set to 2.31 radians (132.4 º), based on a distance of 18.3 Å between phosphorus atoms of opposite strands and a 10 Å helix radius. We define a P-P rung as the segment connecting the phosphorus atoms of opposite stands. The rise between successive base pairs/rung is 3.34 Å along the direction of the cylinder axis, and its rotation, 360/10.5 º (10.5 base pairs per turn). So far, this model does not have any free parameters (although the previous values can be adjusted to alternative values if needed).

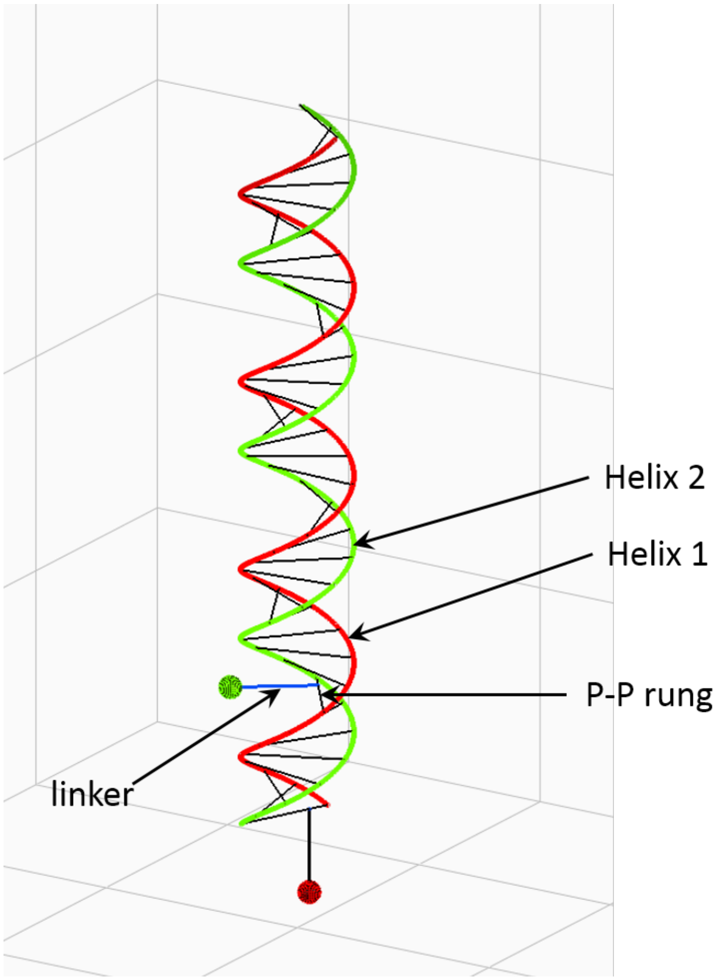

Fig. SI-26: **DNA double-helix model schematic.** perspective view of a double stranded DNA molecule labeled with an acceptor dye (red, bottom) at position 1 and a donor dye (green) at position 7. The molecule is represented by the two phosphate backbone helices and rungs connecting opposite phosphorus atoms, and one linker per dye, each attached to its corresponding rung at a variable location.

To model a dye attached to a base, we introduce three parameters per dye (in practice we used the same set for both dyes): the distance *d* from helix 1 of the "attachment" point *S* (Fig. SI-27B), the inclination ψ of the vector connecting the dye D to S, and

the distance *L* of the dye from this attachment point (Fig. SI-26B). Note that in reality, the dye's linker is attached to a nucleic acid base, which is itself located somewhere in the base pair plane (in other words, the linker attachment point is different from S). However, the exact interpretation of the 3 parameters *d*, *L* and ψ does not really matter as long as the bases to which the dyes in different samples are attached are always the same, as is the case in our study (T = thymine). While we chose the "linker" to be located in the base pair plane (or more precisely, the phosphorus plane) for internal labels, an additional off-plane degree of freedom could be conceivably added to account for subtle conformation details, in the specific case of the terminal acceptor dye (red ball in Fig. SI-26), the linker was rotated 90º off-plane, as this is the most symmetrical (or neutral) conformation of all.

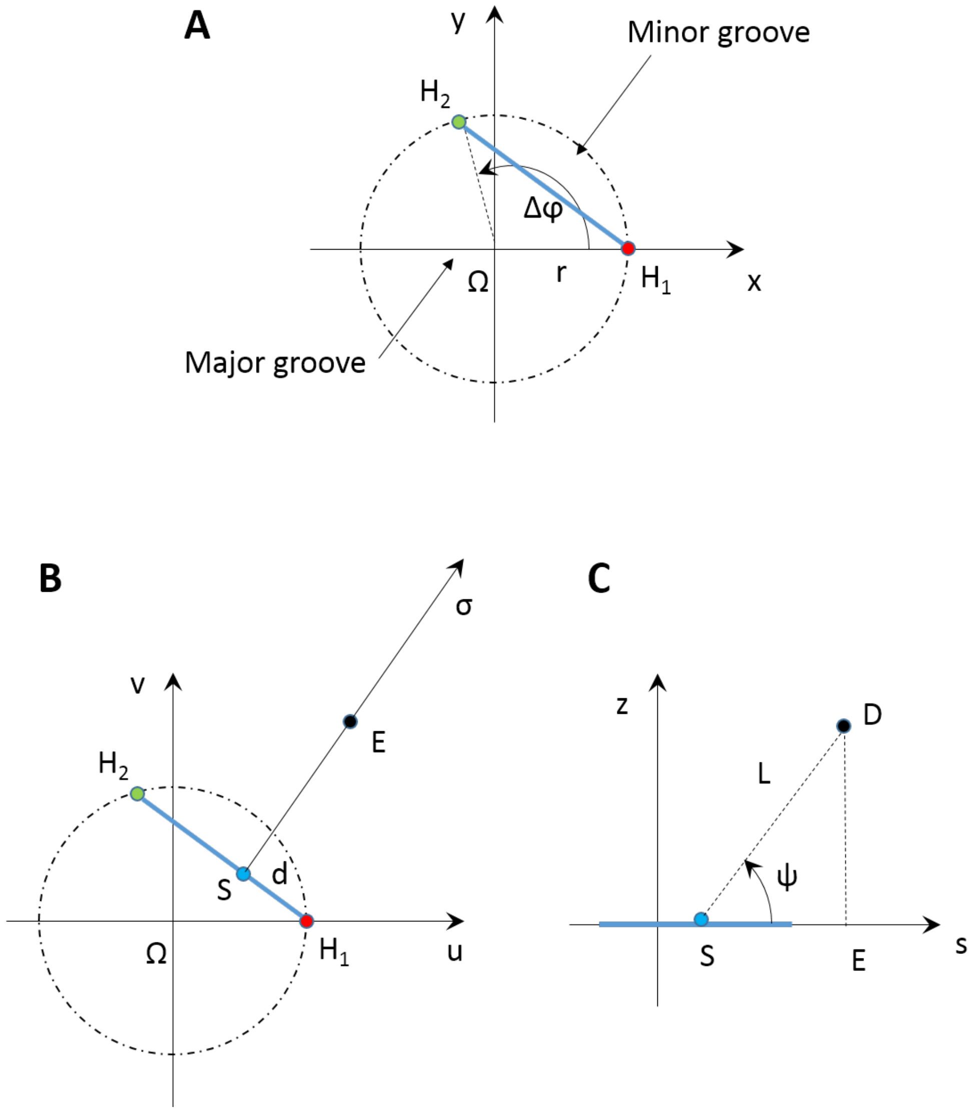

Fig. SI-27: DNA double-helix model details. (**A**) Top view of a base pair plane, showing the two phosphorus atoms (H1 and H2), the DNA molecule axis (Ω). (**B**) Definition of parameter *d* (location of the linker attachment point on the rung) and projection E of the dye center in the rung plane. (**C**) Lateral view and definition of thinclination angle ψ and linker length *L*.

With these definitions in hand, it is simple to derive equations for the position of dyes attached along the DNA stands and their respective distances.

16.2 Model parameterization

Let’s assume (Figure SI-27A) that the first phosphorus of helix 1 is at position (*r*, 0, 0) and the first phosphorus of helix 2 is at position ($r\sin(\Delta\varphi)$, $\mathbf{r}\cos(\Delta\varphi)$, 0). The phosphorus atoms of base pair *i* will have coordinates:

$$
\begin{aligned}
H_{1i} &= \begin{cases} x_{1i} = r\cos\left(\varphi_i\right) \\ y_{1i} = r\sin\left(\varphi_i\right) \\ z_{1i} = i\delta \end{cases} \\
H_{2i} &= \begin{cases} x_{2i} = r\cos\left(\varphi_i + \Delta\varphi\right) \\ y_{2i} = r\sin\left(\varphi_i + \Delta\varphi\right) \\ z_{2i} = i\delta \end{cases}
\end{aligned} \tag{SI.110}
$$

where $\varphi_i = \frac{2\pi}{n} i$ and *n* is the number of base pairs per turn ($n = 10.5$, $\delta = 0.334$ nm, $r = 1$ nm in B-DNA).

The segment connecting the two opposite phosphorus atoms of a given base pair is a convenient reference to localize the average dye position *D* with respect to the double helix. We will project this point on the plane perpendicular to the double helix and defined by the two phosphorus atoms, calling this projection *E* (Figure SI-27B). The orthogonal projection *S* of that point on the H_1H_2 segment will be our reference point for the dye localization and can be defined by:

$$
\overrightarrow{H_1S} = \lambda \overrightarrow{H_1H_2} \ . \tag{SI.111}
$$

In general, $0 < \lambda < ½$, but depending on the circumstances, a negative value of λ or $\lambda > ½$ is conceivable.

The dye’s average position will be specified by its distance *L* to that “anchor” point (this is not a linker length, but is loosely related to it) and its angle ψ with respect to the projection plane (in general, symmetry will impose $\psi = 0$, but some situations may require other values).

Noting $\vec{\sigma}$ the unit vector along segment SE defined as:

$$
\vec{\sigma} = \left(\cos\left(\varphi_i + \frac{1}{2}\Delta\varphi \right), \sin\left(\varphi_i + \frac{1}{2}\Delta\varphi \right), 0 \right) \tag{SI.112}
$$

we have:

$$
\begin{aligned}
\overrightarrow{OD} &= \overrightarrow{OS} + \overrightarrow{SE} + \overrightarrow{ED} \\
&= \overrightarrow{OH_1} + \lambda \overrightarrow{H_1H_2} + L\cos\psi\vec{\sigma} + L\sin\psi\vec{z}
\end{aligned} \tag{SI.113}
$$

And finally:

$$\begin{cases} D_x = r\cos\varphi_i + \lambda r\left(\cos\left(\varphi_i + \Delta\varphi\right) - \cos\varphi_i\right) + L\cos\psi\cos\left(\varphi_i + \frac{1}{2}\Delta\varphi\right) \\ D_y = r\sin\varphi_i + \lambda r\left(\sin\left(\varphi_i + \Delta\varphi\right) - \sin\varphi_i\right) + L\cos\psi\sin\left(\varphi_i + \frac{1}{2}\Delta\varphi\right) \\ D_z = L\sin\psi + i\delta \end{cases} \quad \text{(SI.114)}$$

Note that in this expression L can be positive or negative, depending on whether the dye is located on the minor groove or the major groove side of the double helix.

So far, we have not clarified which strand the dye is attached to. This is because the above definition is dependent on which strand is considered as reference. However, if one dye (A) is attached to one strand and the other (B) to the opposite strand (and in a similar fashion), then for symmetry reasons, we need to impose:

$$\begin{cases} \lambda_A = 1 - \lambda_B \\ \psi_A = -\psi_B \end{cases} \quad \text{(SI.115)}$$

The distance between two dyes (and the corresponding FRET efficiency) is easily obtained from their individual coordinates (Eq. (SI.114)).

The following parameters (A: acceptor dye, D: donor dye) were used in Fig. 7 of the main text: $\lambda_A = 0.256$, $\lambda_D = 0.744$), $L_A = L_B = L = 12.46$ Å, $\psi_A = -90°$, $\psi_D = 0°$.

Appendix 17 RNAP Escape Kinetic Experiments

For comparison, we report in Fig. SI-28 results of the real-time kinetic experiment described in the main text, performed on the single-spot μs-ALEX setup or the multispot setup. The smFRET-μsALEX system has better sensitivity than the current 8-spot setup, due to the higher PDE of the single-pixel SPADs. Nonetheless, due to the increased throughput, the multispot setup shows a drastic improvement in its ability to resolve the kinetics compared to the single-spot μs-ALEX setup.

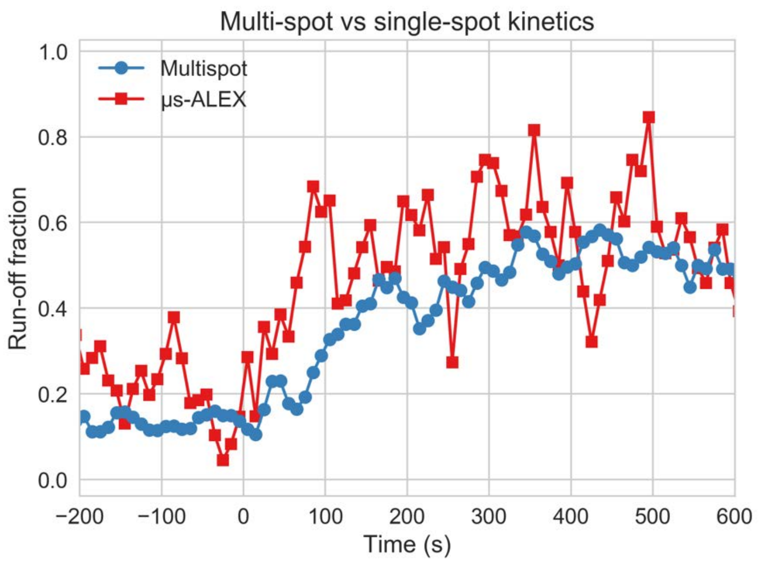

Fig. SI-28. Real-time kinetics measured with the single- and multispot setups. Each time point represents 30 s of measurement. The 30 s integration window is shifted by 10 s steps. For details, refer to the *Realtime Kinetics Analysis* Jupyter notebook (view online).

Appendix 18 References

1. Ingargiola A, Laurence T, Boutelle R, Weiss S, Michalet X. Photon-HDF5: An Open File Format for Timestamp-Based Single-Molecule Fluorescence Experiments. Biophysical Journal. 2016;110(1):26-33. doi: 10.1016/j.bpj.2015.11.013.

2. Ingargiola A, Lerner E, Chung S, Panzeri F, Gulinatti A, Rech I, Ghioni M, Weiss S, Michalet X. smFRET µs-ALEX: 5 dsDNA samples. 2016. doi: 10.6084/m9.figshare.1098961

3. Ingargiola A, Lerner E, Chung S, Panzeri F, Gulinatti A, Rech I, Ghioni M, Weiss S, Michalet X. 8-spot smFRET measurements on 6 dsDNA samples. 2016. doi: 10.6084/m9.figshare.1098962

4. Ingargiola A, Lerner E, Chung S, Panzeri F, Gulinatti A, Rech I, Ghioni M, Weiss S, Michalet X. RNAP Promoter Escape Kinetics Data Files. 2016. doi: 10.6084/m9.figshare.3810930

5. Ingargiola A, Lerner E, Chung S, Panzeri F, Gulinatti A, Rech I, Ghioni M, Weiss S, Michalet X. Afterpulsing ACF Correction Files. 2016. doi: 10.6084/m9.figshare.3817062

6. Ingargiola A, Lerner E, Chung S, Panzeri F, Gulinatti A, Rech I, Ghioni M, Weiss S, Michalet X. Constant Illumination Files Used to Compute Afterpulsing Probabilities for SPAD Arrays. 2016. doi: 10.6084/m9.figshare.4784572

7. Ingargiola A, Lerner E, Chung S, Panzeri F, Gulinatti A, Rech I, Ghioni M, Weiss S, Michalet X. Jupyter Notebooks for Multispot single-molecule FRET: high-throughput analysis of freely diffusing molecules. 2016. doi: not yet attributed

8. Ingargiola A, Lerner E, Chung S, Panzeri F, Gulinatti A, Rech I, Ghioni M, Weiss S, Michalet X. ALiX Scripts. 2016. doi: 10.6084/m9.figshare.3839427

9. Panzeri F, Ingargiola A, Lin RR, Sarkhosh N, Gulinatti A, Rech I, Ghioni M, Cova S, Weiss S, Michalet X. Single-molecule FRET experiments with a red-enhanced custom technology SPAD. Proceedings of SPIE. 2013;8590. doi: 10.1117/12.2003187.

10. Zhang ZC, You Z, Chu DP. Fundamentals of phase-only liquid crystal on silicon (LCOS) devices. Light-Science & Applications. 2014;3. doi: 10.1038/lsa.2014.94.

11. Colyer RA, Scalia G, Rech I, Gulinatti A, Ghioni M, Cova S, Weiss S, Michalet X. High-throughput FCS using an LCOS spatial light modulator and an 8x1 SPAD array. Biomedical Optics Express. 2010;1(5):1408-31. doi: 10.1364/BOE.1.001408.

12. Ingargiola A, Panzeri F, Sarkosh N, Gulinatti A, Rech I, Ghioni M, Weiss S, Michalet X. 8-spot smFRET analysis using two 8-pixel SPAD arrays. Proceeding of SPIE. 2013;8590:85900E. doi: 10.1117/12.2003704.

13. Rech I, Marangoni S, Resnati D, Ghioni M, Cova S. Multipixel single-photon avalanche diode array for parallel photon counting applications. Journal of Modern Optics. 2009;56(2):326 - 33. doi: 10.1080/09500340802318309.

14. Guerrieri F, Tisa S, Tosi A, Zappa F. Two-Dimensional SPAD Imaging Camera for Photon Counting. IEEE Photonics Journal. 2010;2(5):759-74. doi: 10.1109/jphot.2010.2066554.

15. Colyer RA, Scalia G, Villa FA, Guerrieri F, Tisa S, Zappa F, Cova S, Weiss S, Michalet X. Ultrahigh-throughput single-molecule spectroscopy with a 1024 SPAD. Proceedings of SPIE. 2011;7905:790503. doi: 10.1364/BOE.1.001408.

16. Michalet X, Ingargiola A, Colyer RA, Scalia G, Weiss S, Maccagnani P, Gulinatti A, Rech I, Ghioni M. Silicon photon counting avalanche diodes for single-molecule fluorescence spectroscopy. Journal of Selected Topics in Quantum Electronics. 2014;20(6):3804420. doi: 0.1109/JSTQE.2014.2341568.

17. Ingargiola A, Colyer RA, Kim D, Panzeri F, Lin R, Gulinatti A, Rech I, Ghioni M, Weiss S, Michalet X. Parallel multispot smFRET analysis using an 8-pixel SPAD array. Proceedings of SPIE. 2012;8228:82280B. doi: 10.1117/12.909470.

18. Rech I, Ingargiola A, Spinelli R, Labanca I, Marangoni S, Ghioni M, Cova S. Optical crosstalk in single photon avalanche diode arrays: a new complete model. Optics Express. 2008;16(12):8381-94. doi: 10.1364/oe.16.008381.

19. Restelli A, Rech I, Maccagnani P, Ghioni M, Cova S. Monolithic silicon matrix detector with 50 mu m photon counting pixels. Journal of Modern Optics. 2007;54(2-3):213-23. doi: 10.1080/09500340600790121.

20. Gopich IV, Szabo A. Theory of the statistics of kinetic transitions with application to single-molecule enzyme catalysis. Journal of Chemical Physics. 2006;124(15). doi: 10.1063/1.2180770.

21. Gopich IV, Szabo A. Theory of Photon Counting in Single-Molecule Spectroscopy. In: Barkai E, Brown F, Orrit M, Yang H, editors. Theory and Evaluation of Single-Molecule Signals: World Scientific; 2008. p. 181-244.

22. Ingargiola A, Lerner E, Chung S, Weiss S, Michalet X. FRETBursts: An Open Source Toolkit for Analysis of Freely-Diffusing Single-Molecule FRET. PLoS ONE. 2016;11(8). doi: 10.1371/journal.pone.0160716.

23. Press WH, Teukolsky SA, Vetterling WT, Flannery BP. Numerical Recipes in C. The Art of Scientific Computing. 2 ed. Cambridge: Cambridge University Press; 1992.

24. Nir E, Michalet X, Hamadani KM, Laurence TA, Neuhauser D, Kovchegov Y, Weiss S. Shot-noise limited single-molecule FRET histograms: Comparison between theory and experiments. Journal of Physical Chemistry B. 2006;110(44):22103-24. doi: 10.1021/jp063483n.

25. Fries JR, Brand L, Eggeling C, Kollner M, Seidel CAM. Quantitative identification of different single molecules by selective time-resolved confocal fluorescence spectroscopy. Journal of Physical Chemistry A. 1998;102(33):6601-13. doi: 10.1021/jp980965t.

26. Michalet X, Colyer RA, Scalia G, Ingargiola A, Lin R, Millaud JE, Weiss S, Siegmund OHW, Tremsin AS, Vallerga JV, Cheng A, Levi M, Aharoni D, Arisaka K, Villa F, Guerrieri F, Panzeri F, Rech I, Gulinatti A, Zappa F, Ghioni M, Cova S. Development of new photon-counting detectors for single-molecule fluorescence microscopy. Philosophical Transactions of the Royal Society B. 2013;368(1611): 20120035. doi: 10.1098/rstb.2012.0035.

27. Hoffmann A, Nettels D, Clark J, Borgia A, Radford SE, Clarke J, Schuler B. Quantifying heterogeneity and conformational dynamics from single molecule FRET of diffusing molecules: recurrence analysis of single particles (RASP). Physical Chemistry Chemical Physics. 2011;13(5):1857-71. doi: 10.1039/c0cp01911a.

28. Sigworth FJ, Sine SM. Data Transformations for Improved Display and Fitting of Single-Channel Dwell Time Histograms. Biophysical Journal. 1987;52(6):1047-54. doi: 10.1016/S0006-3495(87)83298-8.

29. Carr DB, Olsen AR, White D. Hexagon Mosaic Maps for Display of Univariate and Bivariate Geographical Data. Cartography and Geographic Information Systems. 1992;19(4):228-&. doi: 10.1559/152304092783721231.

30. Lee NK, Kapanidis AN, Wang Y, Michalet X, Mukhopadhyay J, Ebright RH, Weiss S. Accurate FRET Measurements within Single Diffusing Biomolecules Using Alternating-Laser Excitation. Biophysical Journal. 2005;88(4):2939-53. doi: 10.1529/biophysj.104.054114.

31. Ingargiola A. Applying Corrections in Single-Molecule FRET. Biorxiv. 2016:preprint. doi: 10.1101/083287.

32. Jones MC, Marron JS, Sheather SJ. A brief survey of bandwidth selection for density estimation. Journal of the American Statistical Association. 1996;91(433):401-7. doi: 10.2307/2291420.

33. Antonik M, Felekyan S, Gaiduk A, Seidel CAM. Separating structural heterogeneities from stochastic variations in fluorescence resonance energy transfer distributions via photon distribution analysis. Journal of Physical Chemistry B. 2006;110(13):6970-8. doi: 10.1021/jp057257.

34. Churchman LS, Flyvbjerg H, Spudich JA. A non-Gaussian distribution quantifies distances measured with fluorescence localization techniques. Biophysical Journal. 2006;90(2):668-71. doi: 10.1529/biophysj.105.065599.

35. Krichevsky O, Bonnet G. Fluorescence correlation spectroscopy: the technique and its applications. Reports on Progress in Physics. 2002;65(2):251-97. doi: 10.1088/0034-4885/65/2/203.

36. Hess ST, Webb WW. Focal volume optics and experimental artifacts in confocal fluorescence correlation spectroscopy. Biophysical Journal. 2002;83(4):2300-17. doi: 10.1016/S0006-3495(02)73990-8.

37. Enderlein J, I G, Patra D, Fitter J. Art and artefacts of fluorescence correlation spectroscopy. Current Pharmaceutical Biotechnology. 2004;5(2):155-61. doi: 10.2174/1389201043377020.

38. Laurence TA, Fore S, Huser T. Fast, flexible algorithm for calculating photon correlations. Optics Letters. 2006;31(6):829-31. doi: 10.1364/OL.31.000829.

39. Zhao M, Jin L, Chen B, Ding Y, Ma H, Chen DY. Afterpulsing and its correction in fluorescence correlation spectroscopy experiments. Applied Optics. 2003;42(19):4031-6. doi: 10.1364/Ao.42.004031.

40. Schwille P, Meyer-Almes FJ, Rigler R. Dual-color fluorescence cross-correlation spectroscopy for multicomponent diffusional analysis in solution. Biophysical Journal. 1997;72(4):1878-86. doi: 10.1016/S0006-3495(97)78833-7.

41. Thompson NL. Fluorescence Correlation Spectroscopy. In: Lakowicz JR, editor. Topics in Fluorescence Spectroscopy, Volume 1: Techniques. 1. New York: Plenum Press; 1991. p. 337-78.

42. Weidemann T, Wachsmuth M, Tewes M, Rippe K, Langowski J. Analysis of ligand binding by two-colour fluorescence cross-correlation spectroscopy. Single Molecules. 2002;3(1):49-61. doi: 10.1002/1438-5171(200204)3:1<49::Aid-Simo49>3.3.Co;2-K.

43. Lerner E, Chung S, Allen BL, Wang S, Lee J, Lu WS, Grimaud LW, Ingargiola A, Michalet X, Alhadid Y, Borukhov S, Strick TR, Taatjes DJ, Weiss S. A backtracked and paused

transcription initiation intermediate of Escherichia Coli RNA polymerase. Proceedings of the National Academy of Sciences USA. 2016;113(43):E6562-E71. Epub 2016/10/11. doi: 10.1073/pnas.1605038113.

44. Kim S, Streets AM, Lin RR, Quake SR, Weiss S, Majumdar DS. High-throughput single-molecule optofluidic analysis. Nature Methods. 2011;8:242-5. doi: 10.1038/NMETH.1569.

45. Kapanidis AN, Margeat E, Ho SO, Kortkhonjia E, Weiss S, Ebright RH. Initial transcription by RNA polymerase proceeds through a DNA-scrunching mechanism. Science. 2006;314(5802):1144-7. Epub 2006/11/18. doi: 10.1126/science.1131399.

46. Clegg RM. Fluorescence resonance energy transfer and nucleic acids. Methods in Enzymology. 1992;211:353-88. doi: 10.1016/0076-6879(92)11020-J.
